# Toward a detailed understanding of search trajectories in fragment assembly approaches to protein structure prediction

**DOI:** 10.1002/prot.24987

**Published:** 2016-02-23

**Authors:** Shaun M. Kandathil, Julia Handl, Simon C. Lovell

**Affiliations:** ^1^ Faculty of Life Sciencesthe University of ManchesterManchesterM13 9PLUnited Kingdom; ^2^ Alliance Manchester Business School, Faculty of Humanities, the University of ManchesterManchesterM13 9PLUnited Kingdom

**Keywords:** conformational sampling, Rosetta, EdaFold, search heuristic, exploration, multidimensional scaling, entropy

## Abstract

Energy functions, fragment libraries, and search methods constitute three key components of fragment‐assembly methods for protein structure prediction, which are all crucial for their ability to generate high‐accuracy predictions. All of these components are tightly coupled; efficient searching becomes more important as the quality of fragment libraries decreases. Given these relationships, there is currently a poor understanding of the strengths and weaknesses of the sampling approaches currently used in fragment‐assembly techniques. Here, we determine how the performance of search techniques can be assessed in a meaningful manner, given the above problems. We describe a set of techniques that aim to reduce the impact of the energy function, and assess exploration in view of the search space defined by a given fragment library. We illustrate our approach using Rosetta and EdaFold, and show how certain features of these methods encourage or limit conformational exploration. We demonstrate that individual trajectories of Rosetta are susceptible to local minima in the energy landscape, and that this can be linked to non‐uniform sampling across the protein chain. We show that EdaFold's novel approach can help balance broad exploration with locating good low‐energy conformations. This occurs through two mechanisms which cannot be readily differentiated using standard performance measures: exclusion of false minima, followed by an increasingly focused search in low‐energy regions of conformational space. Measures such as ours can be helpful in characterizing new fragment‐based methods in terms of the quality of conformational exploration realized. Proteins 2016; 84:411–426. © 2016 The Authors Proteins: Structure, Function, and Bioinformatics Published by Wiley Periodicals, Inc.

## INTRODUCTION

Predicting protein tertiary structure from sequence information remains an important unsolved problem. Techniques based on the principle of fragment assembly^1^ have emerged as the leading class of methods to tackle this problem, as evidenced by their performance in the CASP experiments.^2^, ^3^ However, their accuracy is known to decrease for larger, more complex proteins.^3^ Typical fragment‐based prediction pipelines employ many independent runs of a prediction technique (the random‐restart strategy) to arrive at a pool of structures, from which a subset of promising predictions are chosen. Recent work has seen the development of advanced sampling protocols that move away from such a “brute‐force” approach. However, these methods seem unable to reliably match or exceed the performance of pipelines based on a large number of restarts. This is a disappointing situation, and it is unclear whether this is due to inaccuracies in scoring functions, poor‐quality fragment libraries or ineffective search methods. Traditional measures of search performance cannot readily disentangle the contributions of these three components, and a detailed understanding of conformational sampling performance remains elusive.

In general, fragment assembly techniques rely on the fact that secondary and tertiary structure can be strongly influenced by local amino acid sequence.^1^ These local propensities are taken into account and exploited during model construction, by deriving fragments from known protein structures and using them as building blocks during the search. The search techniques employed are heuristic optimization algorithms that start from an initial structure (e.g., a fully extended chain), and which iteratively apply randomly selected fragment insertions to generate novel candidate structures. An energy or scoring function is used to determine whether a particular candidate structure should be accepted. A key assumption behind the use of an optimization procedure is that near‐native structures correspond to at least a local optimum in the energy landscape defined by this function.

The accuracy of fragment‐based prediction typically decreases for larger proteins and particularly those with high contact order.^3^ Efforts to overcome this problem have centered around the scoring functions employed, and some deficiencies of knowledge‐based scoring functions have been highlighted.^4^, ^5^, ^6^ As a result, there has been significant progress in improving scoring functions for fragment assembly.^7^, ^8^, ^9^ In addition to scoring functions, methods for generating high‐quality fragment libraries have also been developed,^10^, ^11^, ^12^, ^13^ and the significance of conformational sampling during the prediction process has been studied.^14^, ^15^, ^16^

These key components of fragment‐based methods (scoring functions, fragment libraries, and sampling methods) are highly interdependent. Effective conformational search strategies that are capable of traversing multiple local optima become increasingly important when scoring functions are highly multimodal. Similarly, as fragment quality decreases, a smaller portion of the available search space will correspond to near‐native solutions, and the explorative effectiveness of the search protocol becomes more crucial. These interactions can make it difficult to disentangle the contribution made by the sampling method alone. This problem is exacerbated by the way in which sampling protocols have commonly been assessed. Evaluations based only on the accuracy of the final structures returned by a prediction method are inadequate, since they can confound search performance with the accuracy of the scoring functions and the quality of the fragment libraries used. In other words, an assessment of final structure quality does not provide information about whether a good number of alternative topologies were considered during a search trajectory.

We suggest that more transparent measures of search performance need to monitor the extent to which different methods explore a range of possible “protein‐like” conformations, by considering information about the structures encountered during a search trajectory. In the case of fragment‐based prediction methods, these analyses need to take into account the available search space, as defined by the fragment library. We suggest that this kind of analysis will lead to more concrete evidence regarding the strengths and limitations of different conformational search protocols, and the reasons underlying their continued lack of scalability to large proteins. A more accurate understanding of current search dynamics in fragment assembly is fundamental in order to enable the development of improved sampling protocols.

In this article, we present: (i) a discussion of the limitation of standard measures of performance in monitoring the search performance of fragment assembly; (ii) the description of a set of measures designed to provide a more informative assessment of the performance of search heuristics for this problem; and (iii) the use of these techniques to compare Rosetta, a protocol based on random restarts, and EdaFold, an example of a more advanced sampling strategy; this comparison serves to illustrate the use of our techniques, to shed light on performance differences between these conceptually different approaches, and to identify common limitations that may point the way toward future developments.

### Assessing conformational sampling

As we have discussed above, in protein structure prediction, it is limiting to focus assessment on the quality of the final structure alone. This is because typical energy or scoring functions are not always aligned with the “true” objective of finding near‐native structures. Assessments of search performance should therefore consider the extent to which a range of alternative plausible structures are explored, by considering the trajectories of individual prediction runs as they navigate conformational space. Although one could employ measures such as the root mean squared deviation (RMSD) or energy scores to do this, these measures have limitations when used to monitor search performance.

**Figure 1:**
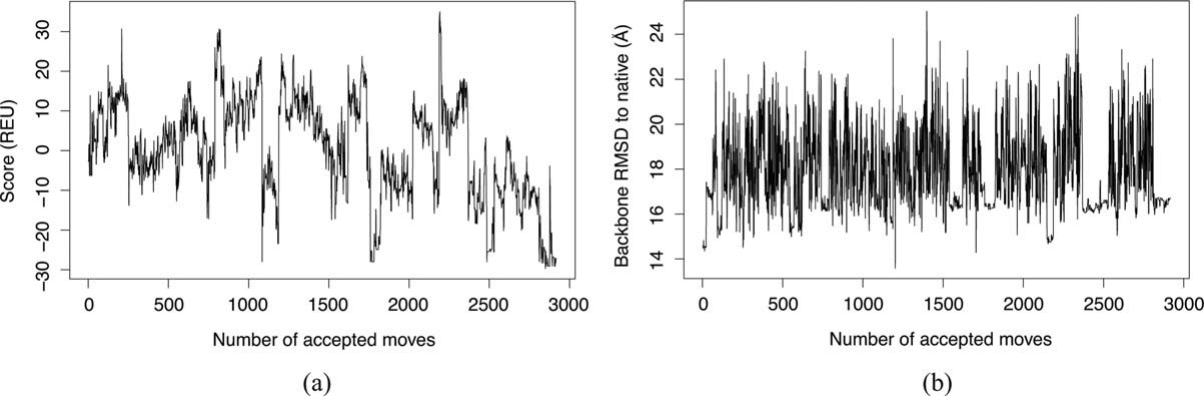
Changes in (**a**) the score (energy), and (**b**) RMSD values of the structure during stage 3 of the low‐resolution search phase of Rosetta, for one run with fibronectin domain (PDB ID 1fna). Score values are in Rosetta Energy Units (REU). The values of these two measures vary considerably during the search, which may be misinterpreted as resulting from good conformational exploration.

First, both measures are sensitive to relatively small changes in protein structure, which makes it difficult to interpret whether distinct conformational states are being explored. Figure 1 illustrates the difficulty of assessing search performance using RMSD from the native and score values. The values of these measures can change frequently as the search progresses, suggesting that the sampling protocol is exploring diverse new conformations. This impression is deceptive, as the search has actually stagnated: most moves in this run correspond to flipping of a single terminal tail, and a meaningful exploration of the search space is no longer taking place.

A second limitation of such measures (in a fragment assembly context) is that they do not consider the available search space (as defined by the fragment library) as a part of the analysis, although this places important constraints on predictive accuracy: in scenarios where high‐quality fragment libraries cannot be reliably generated, it is essential that a good proportion of the compact conformational space is explored. This increases confidence in the prediction, and can more clearly point to potential problems in fragment library generation.

In this work, we propose a set of additional measures that are designed to complement traditional measures of prediction performance, with the aim of obtaining a more complete picture of sampling. The proposed measures directly assess the quality of conformational exploration, at a global (fold) and local (residue) level. The design of three of these measures allows for their definition relative to the available search space, which makes them particularly suited in the context of fragment‐assembly techniques, where the search space is constrained through the fragment library.

Our “local” measures monitor sampling on a per‐residue basis. We determine (i) the frequency with which insertions are accepted in different sections of the protein chain during the search, and (ii) the extent to which the set of available structural parameters (as defined by the fragment library) is utilized for each position in the protein chain. These analyses provide a residue‐specific picture of sampling performance, and allow us to identify sampling biases, e.g. related to the over‐ or under‐sampling of different parts of the chain or different types of secondary structures.

We also define “global” measures which monitor the extent to which structurally diverse conformations are sampled in single trajectories. We quantify differences between structures sampled in a trajectory, based on differences in contact patterns. We use clustering and Markov state model construction, together with a measure based on Shannon entropy^17^ to estimate the extent to which the trajectory is exploring the search space. This quantitative measure of search performance is complemented by a visual representation of a search trajectory moving through the available conformational space. Because the contact‐based dissimilarities are high‐dimensional, classical multidimensional scaling (MDS) is employed for the purpose of visualizing the movement of a search trajectory through the conformational space defined by the set of reference structures.

In the following, we illustrate the use of our techniques on a set of representative fragment‐assembly methods. While many such techniques have been described (e.g., Refs. 18, 19, 20), we select the popular prediction method Rosetta,^1^, ^21^ as well as the recently developed EdaFold program.^22^, ^23^ These methods make use of identical fragment libraries and scoring functions, enabling us to make useful comparisons between their sampling strategies. These methods were also chosen to contrast an example of a restart‐based strategy (Rosetta) with an approach that employs a more advanced optimization strategy (EdaFold). In the case of Rosetta, we also examine two different running modes: the more commonly employed strategy of running many short runs, and single, longer runs. We compare these strategies since it has been observed empirically that a large set of relatively short runs is more likely to produce high‐accuracy predictions than a single, longer run that uses the same total number of insertion attempts.

## METHODS

### Local measures of sampling

As mentioned previously, we define local measures as those that operate on a per‐residue basis. We consider two such measures:

#### Number of successful angle changes per residue

For each residue in the polypeptide chain, we count the number of times that a move changing the corresponding backbone torsion angle triplet (*φ*, *ψ,* and *ω*) is accepted. This allows us to explore differences in fragment insertion acceptance rates in different areas of the chain, for example, different secondary structural elements. Changes are counted following each accepted move.

#### Proportion of available parameter space used

Using the fragment libraries for a given protein, we determine the number of unique backbone torsion angle triplets that are available for each position in the chain, by collecting information about every fragment insertion that can modify that position. We then assess the proportion of these unique triplets that are used at least once during the search, by considering every accepted fragment insertion. This analysis provides a first indication of an algorithm's ability to explore the parameter space available, although it does not consider combinations of angles in different positions.

### Global measures of sampling

We additionally derive global measures of sampling, to study fold‐level exploration in the search trajectories. These measures operate on a search trajectory (or a subset of structures sampled during a trajectory), and evaluates search ability relative to a sample of a the available search space, as estimated by a large decoy data set. We complement this analysis using a low‐dimensional visualization of the search trajectory, with the aim of providing an intuitive understanding of this measure, and more detailed insight regarding the dynamics of the search. Note that, by design, this analysis uses a trajectory as well as a reference set of low‐resolution decoys. For this reason, inferences about sampling quality can be made relative to the conformational space represented by the “background” of decoys. Thus, good search performance of a given sampling protocol may be detected independent of fragment quality, even in cases where the entire conformational space is non‐native (i.e., when there exists no combination of fragments in the library compatible with the native topology).

#### Visualization of trajectory progress using multidimensional scaling (MDS)

Data from folding trajectories is high‐dimensional, as proteins have variable numbers of residues, and each residue has structural parameters associated with it. Using dimensionality reduction techniques, it is possible to represent dissimilarities in high‐dimensional data on a 2D or 3D scatterplot. Such a technique would enable us to visually compare the relationship between a set of structures sampled during a single run of a prediction method, against a larger set of decoy structures. This allows us to investigate the degree to which different structural states are visited during a single run. In other words, we can track the movement of a single prediction run through the space of accessible conformations. Ideally, individual trajectories would visit a good proportion of the available space before “settling” in a localized region. These methods require the assessment of differences between alternative structures for a target, and we require measures that capture fold‐level differences between structures, while reducing the importance given to more fine‐grained differences. We express global fold information using binary contact maps, generated using a distance cutoff of 8 Å between C_*α*_ atoms in any structure. The dissimilarity between such contact maps can be expressed as the Hamming distance,^24^ which counts the number of corresponding contacts in two maps that are in different states. This provides an intuitive and descriptive measure of conformational difference, which focuses on genuine variation in residue–residue contacts, and allows us to evaluate the extent to which different folds and contact patterns are explored. Measures such as RMSD cannot capture this information in the same way (see Supporting Information for an example).

The choice of dimensionality reduction technique is primarily motivated by our distance measure. For example, principal component analysis (PCA) does not allow us to use a non‐Euclidean distance metric between data points. In our study, we employed classical Multidimensional scaling (MDS),^25^ which allows us to start from a pairwise distance matrix generated using any metric measure. This approach has been previously used by Sims *et al*. 26 to construct a global map of the conformational space available for short peptides. We opted to use this method over nonlinear or nonmetric methods for multidimensional scaling (e.g., Sammon mapping), due to the reduced computational requirements of classical scaling for datasets of the size we needed to handle, and due to the fact that the Hamming distance is a metric (i.e., satisfies the triangle inequality).

We now briefly describe the procedure for generating an MDS plot. Given a set of *N* conformations of interest, an *N* × *N* dissimilarity matrix is computed using the Hamming distance measure. Classical MDS (as implemented in R version 3.1.0^27^) is then used to obtain a mapping of this dissimilarity matrix onto two dimensions. The points on the MDS plot correspond to the individual conformations, with more similar conformations plotted closer together. Individual search trajectories are visualized on a “background” of low‐resolution decoys. These low‐resolution decoys are the results of a large number of runs of the Rosetta low‐resolution protocol and will span a portion of the available conformational space. The MDS plot then provides a visual representation of the extent to which a single search trajectory explores this available space. Furthermore, assuming a native structure is available, MDS allows us to track the progress of individual runs or sets of runs to evaluate how well native‐like conformations are sampled, by including the native structure in the pool of structures used to generate the plot.

The MDS plots give us a way of visualizing the movement of search trajectories through conformational space. However, in our plots, the first two principal coordinates capture no more than 36.17% of the total variation in the data (the median is 12.46%). Values for each individual MDS plot are given in the Data Supplement accompanying this manuscript. The low percentage of variance captured appears to be primarily due to the way that structures in each pool are distributed in the high‐dimensional space of contact patterns. Because the amount of variance captured in the MDS plots is fairly small, we recommend that these plots should be used for indicative purposes only. Other methods of dimensionality reduction for protein folding trajectories have been described (e.g., Ref. 28), and we plan to explore alternative ways of achieving good low‐dimensional projections in a more scalable manner in the future. For now, our method has the advantages of being relatively easy to understand, and can be easily replicated using existing “off‐the‐shelf” methods.

#### Quantifying explorative diversity using Markov state models and weighted Shannon entropy

Next, we develop a complementary method that allows us to quantify the exploration of a trajectory in terms of the original high‐dimensional dissimilarity data (thus avoiding the limitations of the MDS plot, discussed above). To do this, we employ Partitioning Around Medoids (PAM) clustering^29^ followed by the construction of a Markov State Model (MSM) to describe the progress of individual trajectories through conformational space. MSM‐based methods have been used to study molecular dynamics trajectories,^30^, ^31^, ^32^ typically using *k*‐means clustering with RMSD as the distance measure. In our study, we used PAM clustering to represent the idea that each cluster center (medoid) should correspond to one of the supplied data points, since calculating an “average” structure could lead to physically impossible configurations (e.g., with a large number of atomic clashes). For the distance measure, we used the Hamming distance between binary contact maps, consistent with the approach taken for the MDS‐based visualization.

**Figure 2:**
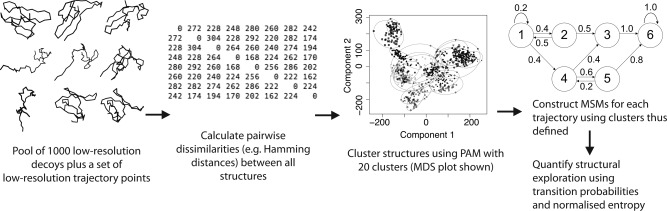
Steps in the construction of Markov State Models (MSMs) for multiple trajectories. The pairwise distances in step 2 can be calculated using different metrics.

The procedure for this analysis is illustrated in Figure 2. Briefly, data from a small set of trajectories and large number of low‐resolution decoys are clustered using PAM. This sets up a system of clusters that should correspond to different structural features. Because the temporal ordering of the structures in individual trajectories is known, the cluster assignments for individual structures from each trajectory gives us the order in which the various clusters have been visited during the run. In this way, we can construct a “route map” of the trajectories moving through different regions of conformational space, sampling different structural features as they proceed.

Following this, we construct a Markov state model of the trajectories moving through the defined clusters. A state model shows us which clusters are most frequently visited, and it also allows for quantitative analysis of transition frequencies between different clusters, through the use of measures such as Shannon entropy.^17^ Shannon entropy quantifies the amount of disorder in a system that can exist in one of many states. In our case, this corresponds to the polypeptide chain undergoing a particular transition between two states (clusters) that correspond to distinct contact patterns. The entropic contribution from a given transition is weighted by the structural difference introduced by that transition,^47^ and normalization is performed using the theoretical maximum possible entropy for the given number of clusters and set of weights (see Supporting Information for details).

The resulting entropy values take a maximum value of 1 (high uncertainty) and a minimum value of 0 (low uncertainty). A trajectory that effectively samples conformational space will undergo many different transitions, resulting in a high value of normalized entropy, while low entropy indicates that the trajectory is following deterministic paths through conformational space (“going round in circles”), or that the trajectory is confined to a localized area of conformational space.

### Prediction methods

**Figure 3:**
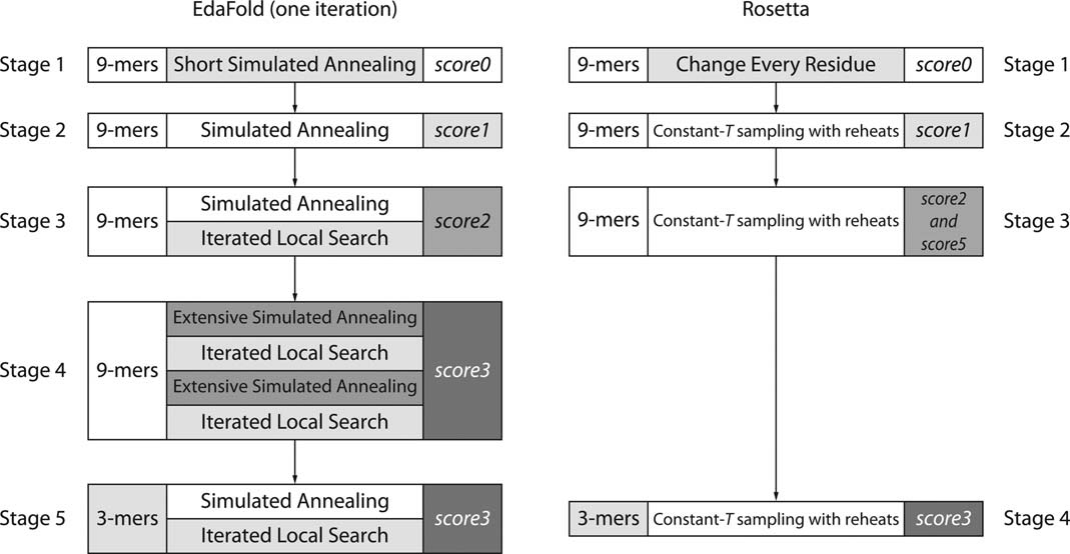
Stages in one iteration of the EdaFold *sample_and_minimize* procedure, with the comparable stages of Rosetta's low‐resolution protocol. Note that while EdaFold uses multiple rounds of the whole 5‐stage procedure in each run, Rosetta uses only a single pass through its stages per run. Each stage consists of a fragment library, a conformational sampling procedure, and one or more scoring functions. In some stages of EdaFold, the conformational sampling procedure involves multiple, sequential steps. The “short” simulated annealing procedure uses half the number of attempts as the regular simulated annealing procedure, while the “extensive” version involves two rounds of the regular simulated annealing procedure. Following stage 5 of the *sample_and_minimize* procedure, a *FastRelax* all‐atom refinement step is used to derive energy values.

We briefly summarize key aspects of the fragment‐based prediction methods studied in this work. Both Rosetta and EdaFold consist of low‐resolution and all‐atom phases. The former phase is designed to enable rapid conformational exploration to derive a plausible topology for the given target protein, while the latter is designed to perform detailed local refinement of the structure by detailed optimization of backbone and side‐chain geometry. For our analyses, we focus on the low‐resolution phase only, as this is where fragment‐based conformational searching takes place. The low‐resolution phase of each method is divided into a number stages (Fig. 3), employing different fragment libraries, scoring functions, and in the case of EdaFold, different search algorithms.

#### Rosetta

In Rosetta's low‐resolution search, bond lengths and angles are initially set to ideal values and amino acid side chains (except glycine) are approximated by centroid atoms connected to the *C*_β_ atom. Starting from a linear configuration, progressive folding of the protein is achieved through successive fragment insertions, or “moves.” A Monte Carlo sampling scheme is used, with a fixed value of the Metropolis temperature parameter *kT =* 2. If the search appears to stagnate (specifically, if no move has been accepted for 150 iterations), the temperature parameter is temporarily increased, and reset to its initial value once a move has been accepted. The rationale for using such a scheme is that it should allow the search to identify and escape shallow local minima in the energy landscape, by temporarily allowing the energy value to increase. These default parameter settings are chosen as a result of extensive benchmarking. Evaluation of the Metropolis criterion requires the calculation of energy differences between successive conformations, and the energy of a given conformation is evaluated using a knowledge‐based energy function.^1^, ^33^

The low‐resolution protocol of Rosetta comprises four stages (Fig. 3), which differ in the length of the fragments and the energy functions used. The first three stages employ 9‐residue fragments (9‐mers), while the fourth stage uses 3‐residue fragments (3‐mers). The search uses the best 25 and 200 fragments per insertion window for the two fragment libraries, respectively.^21^ Fragments are ranked and chosen based primarily on local PSI‐BLAST^34^ profile similarity to proteins of known structure, together with other similarity metrics such as agreement with predicted secondary structure.^10^, ^21^

The low‐resolution Rosetta scoring function consists of a linear weighted sum of ten scoring terms.^21^ The terms include descriptions of steric repulsion and compactness, as well as statistical potentials for interactions between specific elements of secondary structure. The weights of the terms in the energy function are gradually increased to their final values as Rosetta proceeds through the low‐resolution stages, and these different weight settings are denoted *score0* through *score5* (Fig. 3). Through the use of the different weight settings, the scoring functions become increasingly selective as to the acceptance of various fragment insertions as the search proceeds. In stage 3, two scoring functions are used in alternation with a view to reward certain structural features. A more detailed description of Rosetta is available in Ref. 21.

#### EdaFold

EdaFold^22^, ^23^ makes use of the Rosetta framework for fragment libraries, scoring functions and low‐resolution representation of the protein chain. In contrast to typical Rosetta pipelines however, EdaFold uses multiple barrier‐synchronized trajectories running in parallel, sharing information about which fragment insertions lead to lower energy values. In this way, the algorithm seeks to make an informed choice of a conformational subspace within which to focus its search. Because this subspace is defined using low‐energy structures, the expectation is that this will correspond to native‐like conformations, and thus that the method will sample native‐like structures with greater frequency.

This narrowing down of the conformational space to search is carried out using an estimation of distribution algorithm (EDA)^35^ that defines a probability distribution over the fragment library. The algorithm starts by defining a uniform probability distribution over all fragments, and conformational sampling is implemented through multiple simulated annealing steps that employ the Rosetta low‐resolution energy functions, together with iterated hill‐climbing steps that successively perturb and refine the structure in a coarse manner. By default, each simulated annealing step starts from a temperature parameter value of *kT* = 3.5 and ends at *kT* = 0.5. The iterated hill‐climbing steps encourage broader exploration by allowing the search to accept a few fragment insertions without considering the change in energy introduced. Following this, a regime of greedy minimization is used, whereby only moves that reduce the energy of the system are accepted. This combination of perturbation and greedy minimization is repeated a set number of times. The simulated annealing and iterated local search steps are carried out by EdaFold's *sample_and_minimize* function, which can be divided into 5 stages. The first 4 stages employ 9‐mer fragments, whereas the final stage uses 3‐mer fragments. Figure 3 summarizes the steps that the algorithm follows.

Following each call to the *sample_and_minimize* function, fragments found to be associated with energetically favorable structures are given a higher probability of getting inserted relative to those that produce less favorable conformations. In the more recent version of EdaFold, called EdaFoldAA,^23^ energy values are obtained following an all‐atom refinement step using Rosetta's *FastRelax* method.^36^ In both versions of EdaFold, the program collects information about which fragments are present in the structure (fragment keys) following stage 4 (9‐mer fragment insertions). The subsequent steps of the *sample_and_minimize* procedure, including the *FastRelax* step, are used to refine the structure and reach a local minimum and get the final energy value. Once the fragment keys and energy values have been collected for a set of structures, a probability distribution is derived over the fragments present in a subset of decoys with the most favorable energy values. Further rounds of the entire *sample_and_minimize* procedure can be carried out using the updated probability distribution, and this process can be repeated as many times as desired, updating the probability distribution each time. We will refer to one repetition of the *sample_and_minimize* procedure, together with one round of the EDA procedure, as one “iteration” of EdaFold.

### Experimental setup

We used the AbinitioRelax application from Rosetta version 3.4, and EdaFoldAA obtained from the webpage of the Structural Bioinformatics team at RIKEN (http://www.riken.jp/zhangiru/software.html). Running parameters are summarized in Table 1. We modified the source code of Rosetta and EdaFoldAA to output information during the prediction process, including torsion state along the chain, the current stage of the low‐resolution protocol and the number of torsion changes for each position, as well as a PDB structure file every 100 accepted moves. In the case of EdaFold, the iterated local search steps were made to output a PDB structure each time a local minimum was reached at the end of a greedy minimization step. The structure files were used in our MDS and clustering procedures, together with a decoy set of 1000 low‐resolution decoys. These decoys are intended to give an indication of the available compact structural states for each target.

**Table 1:** Parameters for Rosetta and EdaFoldAA

Method	Parameter name	Purpose	Value
Rosetta	‐nstruct	Number of output structures	1
	‐abinitio:increase_cycles	Multiplier for default number of move attempts in each stage	10 or 100
	‐out:pdb	Output pdb files	
EdaFoldAA	–nbThreads	Total number of structures to generate across all iterations of *sample_and_minimize*	1000
	–nbTotIter	Total iterations of *sample_and_minimize* procedure per trajectory	4
	–ILSmaxIter	Maximum number of iterations for iterated local search	3
	–frag3MaxIter	Maximum number of iterations for iterated local search with 3‐mer fragments	2
	–TopPopRate	Fraction of decoys in each iteration used when calculating probability distributions	0.3

All other parameters were left set to their default values.

**Table 2:** The Three Reference Targets Used, With Secondary Structure Types and Length

PDB ID	SS class	Length (residues)	Brief description and source organism
1enh	All‐*α*	54	Engrailed homeodomain (*D. melanogaster*)
1acf	*α* + *β*	125	Profilin IB (*A. castelanii*)
1fna	All‐*β*	91	Cell adhesion module of Fibronectin (*H. sapiens*)

To demonstrate the use of our measures to evaluate the effect of parameter settings on search quality, we considered two values of the increase_cycles parameter in Rosetta (Table 1). This parameter is a multiplier for the default number of fragment insertion attempts in each low‐resolution stage, and so controls the length of the optimization procedure.

For the longer Rosetta runs, the increase_cycles parameter was set to 100. The trajectory from each such run was then compared with a set of 10 shorter runs (using increase_cycles 10), to ensure an approximately similar number of insertion attempts. We ran 20 replicates of each running mode. In the case of EdaFold, an equivalent number of runs in each replicate would result in too few structures being used to determine the probability distribution at each iteration. Therefore, we used an increased value of 0.3 for the TopPopRate parameter (Table 1) and a pool of 250 structures to determine probability distributions in each iteration. We then used data from only a subset of the parallel runs, corresponding to a similar number of insertion attempts as used with Rosetta. This gave us a set of 10 trajectories from each run of EdaFold. While these running parameters are significantly lower than those used by Simoncini *et al*. in their papers, the purpose of our study is fundamentally different. Rather than attempting high‐accuracy prediction, we wish to study the explorative behavior of the sampling algorithms used. In addition, we were prevented from using a large number of replicate runs for our analyses due to computational costs introduced by large additional data files and the use of large pairwise distance matrices for the clustering and MDS procedures. Thus, we used a set of 8 replicate runs for the global analyses, and all 20 replicates for all other analyses.

### Targets and fragment libraries

Analysis was conducted on a set of 59 proteins from the PDB, which were used in a previous study.^37^ We illustrate key results on three targets (Table II) in this manuscript. Information and results for all 59 targets can be found in the supplementary materials accompanying this article. We excluded homologues when generating fragment libraries for these targets, using a local installation of the Rosetta fragment picker. Secondary structure predictions were generated using PSIPRED^38^ version 3.3. We used information from these secondary structure predictions when plotting results for our local measures (as opposed to DSSP^39^, ^40^ assignments of the native structure), since the PSIPRED prediction is used during the fragment generation process. Identical fragment libraries were supplied to all running protocols.

### Statistical analysis for entropy calculations

We analyzed the statistical significance of the differences in median entropy values between the three running protocols considered here, as well as when comparing successive iterations of EdaFold. Our data forms a complete block design with eight replicates per treatment‐block combination. In our case, the blocks correspond to different target proteins, and the “treatment” factor levels correspond to the different running protocols (or iterations of EdaFold). This allows us to compare different protocols or iterations of EdaFold while controlling for variation between different proteins. We used the Mack‐Skillings test (described in Ref. 41, and sections 7.9 and 7.10 in Ref. 42). This provides a non‐parametric test of general alternatives for a replicated block design, and provides a post‐hoc multiple comparisons method to determine which differences between treatments are significant. More details on the statistical procedures are given in the Supporting Information.

## RESULTS

We apply our measures of sampling performance to the low‐resolution phases of three fragment‐based protocols: (1) sets of short Rosetta runs, corresponding to the commonly used random‐restart strategy; (2) single long Rosetta runs, each of which uses the same computational budget as a set of short runs; and (3) EdaFold, an example of a more advanced sampling strategy. We are interested in whether these strategies show differences in the way they navigate the given search space (defined by the fragment libraries). Exact running parameters for the three protocols are given in the Methods and Table I. For brevity, we will illustrate some of our results using just three targets, with PDB identifiers 1acf, 1enh and 1fna (Table II). We chose these targets based on difficulty in prediction when using 1000 unbiased short Rosetta runs: 1enh is an all‐*α* target for which prediction is easy, whereas structures for the *α* + *β* target 1acf and the all‐*β* target 1fna are more difficult to predict. This allows us to illustrate the differences in our measures when studying easy‐ and hard‐to‐predict targets. Complete results for all 59 targets in our dataset can be found in the supplementary materials accompanying this article, including information about typical predictive accuracy obtained when using Rosetta and EdaFold.

### Consistency of sampling across different protein regions

#### General findings

**Figure 4:**
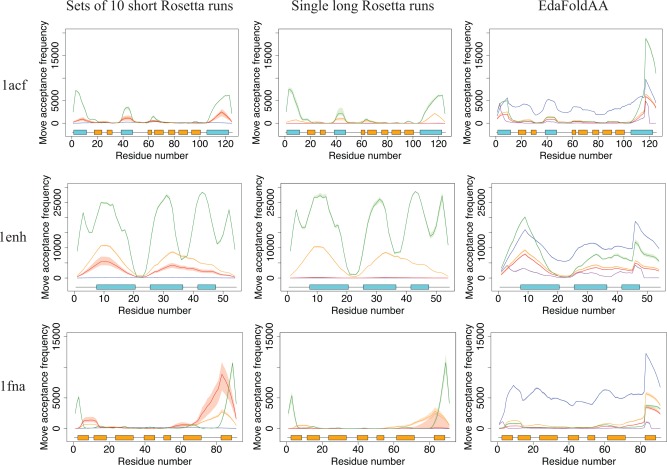
Plots of move acceptance frequency per residue for three targets, for each of the three sampling protocols considered. Median values are plotted for the different stages of the low‐resolution protocol for Rosetta and EdaFold: stage 1 (blue), stage 2 (orange), stage 3 (red), stage 4 (green) and stage 5 (purple, EdaFold only). The shaded regions represent the interquartile range (*n* = 20). At the bottom of each plot is a representation of the secondary structure elements along the target sequence. Helical regions are in blue, and *β*‐strands are in orange. In general, moves tend to be accepted with greater frequency in helical regions and disordered termini in Rosetta, though the former is less pronounced in the case of EdaFold. [Color figure can be viewed in the online issue, which is available at wileyonlinelibrary.com.]

**Figure 5:**
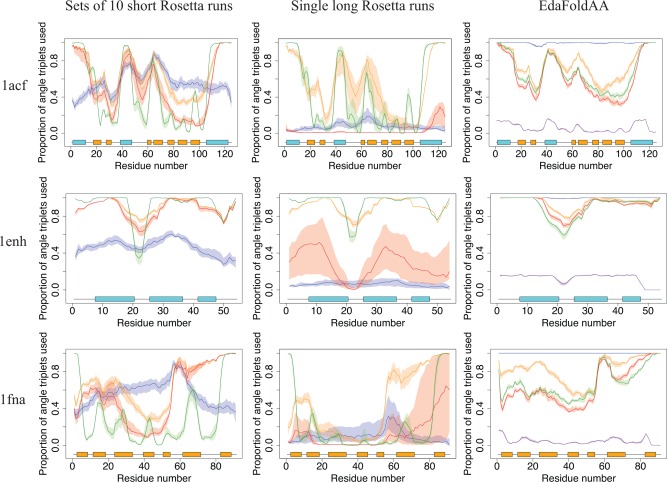
Plots of torsion space usage for three targets. For each residue, the proportion of unique backbone torsion angle triplets used is evaluated, and median values are plotted for each stage, using the same representations as in Figure 4. The single long runs of Rosetta explore a reduced fraction of the available torsion space per residue, as compared with sets of short Rosetta runs, or EdaFold. Stage 1 of EdaFold explores a very high fraction of the available torsion space per residue, but this may not correspond to selective evaluation of protein‐like features (see main text). [Color figure can be viewed in the online issue, which is available at wileyonlinelibrary.com.]

Figures 4 and 5 illustrate the results obtained when using our local measures of sampling on a small set of runs of Rosetta and EdaFold, for three targets. We find that, for all three sampling protocols, the relative frequency of accepted moves is higher in *α*‐helical and terminal regions, though this effect is less pronounced in the case of EdaFold. This is perhaps unsurprising: since fragments for *α*‐helical stretches will likely derive from helices in the templates (which will mostly be similar), it is energetically much more favorable to accept moves in these regions than others. Similarly, disordered termini tend to have much greater freedom of movement, and thus moves in these regions are easy to accept without incurring large energy penalties.

The effect is particularly striking for the all‐*β* domain of fibronectin (1fna), for which the large majority of moves accepted in stages 2, 3, and 4 correspond to fragment insertions affecting approximately the last fifteen residues of the protein only. In general, the results (also data supplement for results across all 59 targets) indicate that the discrepancies observed between the acceptance of moves in different protein regions increase for large proteins and those containing *β*‐sheets. This clearly points to deficiencies of the sampling protocols considered: most fragment insertions in *α*‐helical regions and termini contribute little to a genuine exploration of the conformational search space for a target. Other features, such as disordered loop regions, are arguably more important in determining the spatial arrangement of secondary structural elements and thus the overall fold. Additionally, such replacements are still considered accepted moves within the Monte Carlo sampling framework, and may thus prevent the search protocol (or a human decision‐maker monitoring energy or RMSD scores) from detecting when the search is stagnating.

Limitations in the sampling of loop regions are further confirmed through the analysis of the proportion of the available torsion space explored per residue (Fig. 5). This, again, highlights discrepancies between the utilization of the available parameter space, with significantly better coverage across *α*‐helical regions.

In addition to these common trends, we also identify pronounced differences between the three search protocols.

#### *Short* versus *long rosetta runs*

Anecdotally, we expect an improved exploration behavior in sets of short Rosetta restarts (compared with long Rosetta runs), and some of the reasons behind this are highlighted by our analysis: if we consider the proportion of available torsion space explored per residue (Fig. 5), we see that, throughout all stages, sets of short Rosetta runs are able to explore a higher fraction of the available backbone torsion space, compared to individual long Rosetta runs. Particularly interesting is the increased proportion of torsion space explored in loop regions and *β*‐strands, suggesting that individual short runs in a set are able to explore different arrangements of secondary structure elements, and probably distinct folds. The fact that long runs are unable to achieve the same amount of exploration in loop regions points to one of the persisting limitations of fragment assembly techniques: current methods quickly converge to a compact structure and are then unable to escape this local optimum. It would appear that this underlies the current dominance of restart‐based approaches, as short runs benefit from the structural “randomization,” notably in loop regions, that arises from restarts from a fully extended configuration. Further, once a structure has collapsed into a compact conformation, moves or sequences of moves that cause large structural change, for example, a rearrangement of *β*‐strands, would face a very high energy penalty and would not be accepted in the Monte Carlo framework. However, such moves are necessary to realize good fold‐level exploration, and they are indirectly provided by using a set of restarts.

For stage 3, a comparison of acceptance frequencies highlights another facet to this finding: when comparing the two running modes in Rosetta, we see that single long runs accept far fewer moves in stage 3 (red lines). Rosetta employs a convergence checking mechanism which terminates stage 3 if the structure does not change by 3 Å RMSD relative to a chosen point in the trajectory, every 100 accepted moves. The convergence checker was activated in all our long runs, suggesting that the structures converged to local optima. This is also why the long runs display decreased move acceptance in stage 3. This use of the convergence checker in stage 3 (described above) can be seen as a way of improving efficiency when using a set of restarts, as it avoids redundant local exploration within a trajectory (even in long runs) that does not alter the structure significantly. However, the convergence check uses RMSD to make its decision and it may thus miss convergence: as discussed previously, the use of RMSD alone can be misleading if only certain features of the structure are being altered, with terminal regions being particularly prone to this effect. Furthermore, Rosetta does not incorporate any direct mechanisms to react to the early convergence and restart the search in a different local optimum.

#### *Rosetta* versus *EdaFold*

In comparison to Rosetta, EdaFold trajectories explore a higher fraction of the available torsion space, in all of its stages. In general, the fraction of torsion space explored is comparable or higher than that explored by sets of short Rosetta runs. An improved performance is particularly noticeable in loop regions, thus pointing to an increased diversity in terms of the structures sampled. This is in line with the fact that a single EdaFold “run” is actually a set of trajectories running in parallel, sharing information among themselves at certain points in the search. Furthermore, the iterated local search procedures embedded in each of EdaFold's *sample_and_minimize* steps allow the search to forcibly accept moves without attention to their impact on score values, and this has the potential to allow the search to escape local minima in the energy landscape.

The data in Figures 4 and 5 show pronounced differences between the protocols in stage 1, where EdaFold tends to accept a much larger number of moves and explore a higher fraction of the available torsion space, compared with Rosetta in either of its running modes. This is probably due to differences in the convergence criteria as Rosetta performs fragment insertions until a move has been accepted at least once in every residue,^21^ while EdaFold uses a set number of attempts. Because the scoring function in stage 1 (*score0*) consists of only the van der Waals (*vdw*) term of the Rosetta low‐resolution scoring function,^21^, ^37^ this stage does not make use of any of the statistical potential terms of the energy function and mostly accepts moves that avoid self‐intersecting conformations. Hence, while EdaFold's increased exploration in this stage may act as an advantageous “randomization” step for the initial phases of separate trajectories, the overall impact of this exploration may be limited, as there is no selection for protein‐like features at this stage (and genuine local minima are therefore unlikely to be accessed).

### Exploration of structurally diverse conformations

#### General findings

**Figure 6:**
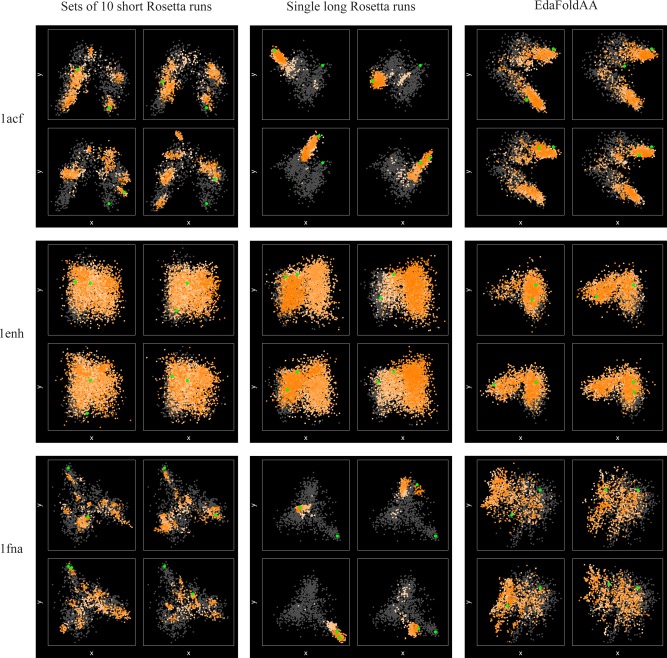
MDS plots for targets 1acf, 1enh and 1fna using the contact map representation, for trajectories generated by sets of short Rosetta runs, single long Rosetta runs, and EdaFold. Data are plotted for four trajectories per target and sampling protocol, and points are generated every 100 accepted moves. The trajectories are plotted against a “background” set of 1000 low‐resolution Rosetta decoys. Structures from stage 1 of each iteration are excluded while plotting points for EdaFold runs. Dispersed sets of points for 1enh indicate that for all three methods, broad conformational sampling is achieved. In the case of 1acf and 1fna, single long runs of Rosetta quickly descend into local minima and are unable to escape, whereas the other two sampling protocols realize greater conformational diversity, clearly indicating the benefits of these running strategies.

Our global analysis of sampling indicates that the reduced degree of acceptance in loops (see above) leads to limitations in fold‐level exploration. Figure 6 compares the MDS visualizations obtained for 3 targets using sets of short Rosetta runs, single long Rosetta runs and EdaFold trajectories. For all three protocols, it can be seen that for 1enh, an easy target, individual runs sample a good proportion of the conformational space corresponding to low‐resolution decoys. Each MDS plot in this work consists of four subplots in principal coordinate space, each containing one trajectory plotted as colored points, with deeper colors toward the end of the run. In the case of sets of short Rosetta runs and EdaFold, each individual trajectory in a replicate is colored individually. Thus, deeper colors correspond to the final stages of each individual trajectory in a replicate; this allows us to identify whether individual trajectories are following similar or dissimilar paths through conformational space. The native structure is highlighted as a single point whose position does not vary between plots for a given protein and prediction method. A second highlighted point represents the last structure in a trajectory or set of trajectories; the location of this point varies between plots generated for each target and protocol. Trajectories are plotted against a “background” of gray points representing low‐resolution decoys, as discussed in the Methods. In the case of EdaFold, we excluded data points from stage 1 of each iteration and run, as this part of the trajectory was thought to be far away from any local optima (see above).

**Figure 7:**
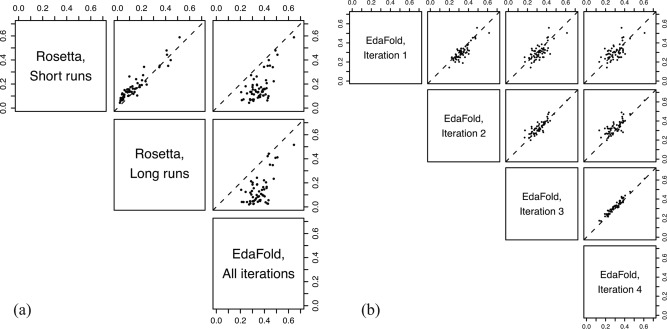
Pairwise scatterplots comparing median entropy values for (**a**) all three sampling protocols considered, over complete runs, and (**b**) individual iterations of EdaFold. In each plot, a dashed line of unit slope is drawn to aid interpretation. Higher entropy values correspond to an improved degree of conformational exploration. Sets of short Rosetta runs exhibit higher median entropy values as compared with single long runs. EdaFold exhibits higher entropy overall, as compared with both running modes of Rosetta. Within EdaFold runs, entropy values increase from iteration 1–2, and then gradually decrease in successive iterations. This suggests that the EDA procedure has two distinct effects on sampling behavior (see Detailed Analysis of EdaFold Across Iterations section).

Figure 7(a) compares median entropy values obtained for each running protocol, for 58 targets (entropy values for one target, 1ail, could not be calculated for short Rosetta runs due to the very large size of that dataset). Additionally, we calculated entropy values for every iteration of each EdaFold run [Fig. 7(b)], to compare the change in the degree of exploration as the search proceeds. All trends of entropy discussed here are statistically significant at the *α* = 0.05 level, after corrections for multiple testing (see Supporting information).

#### *Short* versus *long rosetta runs*

For the hard targets (1acf and 1fna), the MDS plots show that individual long runs remain tightly confined to localized areas of conformational space, which correspond to local energy minima (Fig. 6). This is consistent with the results from the previous sub‐section, also confirming the rapid convergence to local optima seen for these targets. Sets of short runs, on the other hand, are able to explore a greater variety of structure types, even though individual runs are confined to localized areas. These results are further confirmed by entropy calculations on the associated trajectories [Fig. 7(a)]. Sets of short Rosetta runs are typically associated with higher median entropy values, as compared to single long runs.

Overall, this highlights why running sets of short runs continues to be a preferred way of running Rosetta: between them, short runs are able to provide a good degree of fold‐level exploration, while longer runs tend to get stuck in a single local minimum.

#### Detailed analysis of EdaFold across iterations

Given the iterative design of EdaFold, it is interesting to consider the progress of the search through its iterations. Using our entropy measure, we are able to detect two distinct effects of the use of probability distributions during EdaFold runs, which are not obvious from looking at RMSD or energy values alone. We used four iterations of the *sample_and_minimize* procedure.

Within EdaFold runs [Fig. 7(b)], we see that in most cases, entropy increases going from iteration 1 to 2, followed by a gradual decrease in entropy going through the remaining iterations. The gradual decrease in entropy may be expected, as it is in line with the basic aims of an EDA: it indicates that the EDA is successively refining and narrowing down the extent of the search space explored, by identifying fragments that lead to lower all‐atom energy values.

In contrast, the initial increase in entropy (from iteration 1 to iteration 2) is unexpected: for iteration 2 to exhibit higher median entropy values than iteration 1, the search must explore more widely as compared with iteration 1, which uses a uniform probability distribution over the fragments. This finding may point to redundancy in the fragment library, which could explain this counter‐intuitive effect: for example, consider a case where 20% of the fragment set for a region corresponds to an *α*‐helix, and the remaining 80% corresponds to a *β*‐strand. During iteration 1, the chances of inserting fragments of each secondary structure type in the relevant region will be close to these initial percentages (due to the use of a uniform distribution). If the probability distribution determined after iteration 1 then favors the under‐represented secondary structure type (in this case an *α*‐helix), this would lead to a relative increase in the frequency of its insertion in iteration 2. This would translate to more diverse sampling at a global level, as the folding process would move between these structural states more frequently.

#### *Rosetta* versus *EdaFold*

In the case of EdaFold, the MDS plots reveal a pronounced difference in the sampling of harder targets: here, individual runs within a replicate exhibit much greater diversity in their sampling, compared with Rosetta runs. This difference is likely due to the restarts EdaFold performs after each iteration, as well as the iterated local search steps in its *sample_and_minimize* procedure. This finding is further confirmed by the results from the entropy measure: comparing Rosetta and EdaFold over complete runs, we see that EdaFold typically exhibits a higher median entropy as compared with sets of short Rosetta runs. Even though the two methods employ a similar number of scoring function evaluations, EdaFold is clearly able to realize a greater degree of conformational exploration. Importantly, EdaFold already achieves this improved sampling performance in its first iteration, when the core EDA mechanism (the guidance of fragment selection through a probability distribution) has not yet kicked in.

Overall, the EDA appears to provide some effective mechanisms toward controlling the tradeoff between exploration and exploitation in the low‐resolution search, but some of these have been given little emphasis in its original description. Beyond the basic mechanism of the EDA, it appears that key design aspects that contribute to improved conformational exploration may be the use of simulated annealing steps starting at an elevated temperature (compared to Rosetta), and, most importantly, the use of iterated hill‐climbing steps that introduce forced structural perturbation during the search (Quantifying Explorative Diversity Using Markov State Models and Weighted Shannon Entropy section). This allows the search in EdaFold to perform more detailed exploration of the available conformational space, and in doing so, enables EdaFold to make more informed choices of local subspaces within which to conduct more detailed sampling. A good balance between exploration and exploitation may be a key reason why EdaFold is able to achieve improved distributions of predictive accuracy for some targets (see Data Supplement), though this improvement is not seen consistently.

## CONCLUSIONS

In recent years, many new protocols seeking to improve the quality of conformational exploration have emerged, with a view to move beyond the use of many short runs of structure prediction methods. By making use of the structural diversity encountered during the search, these methods guide conformational exploration toward the native state; EdaFold is just one example of such a technique. Other recent innovations include tree‐based search structures^43^ that take both the energy values as well as structural diversity into account when deciding on new search directions, and population‐based evolutionary algorithms^44^ employing structure‐based crossover operators between promising structures encountered during the search. There has also been work describing the use of fragment‐derived structural information to bias folding trajectories employing molecular dynamics (MD),^45^, ^46^ with the authors reporting comparable or improved results as compared with using MD simulation alone. However, whether the use of such protocols enables better conformational exploration is not always clear from the empirical evaluation presented in these and other studies.

Here, we suggest that, using typical measures of predictive performance alone, it can be difficult to assess the extent to which the available conformational space in a fragment‐based prediction run is explored. A better understanding of conformational search is important, since good conformational sampling remains a key limiting factor in the ability to predict the structures of larger targets.^14^ We have therefore developed a set of dedicated techniques that monitor sampling in more detail and are intended to complement traditional assessment techniques. We have illustrated their use in the context of analyzing search trajectories from Rosetta and EdaFold.

Our local measures demonstrate that for both methods, certain structural features in the target proteins are easier to sample, and moves in these regions tend to be accepted with greater frequency. This reflects the relative ease of movement of some parts of the protein chain (such as termini), whereas other parts such as *α*‐helices are easy to sample because of their relative structural invariability. This effect means that other parts of the chain (loop regions and *β*‐strands) are less likely to experience structural variation during the search. Our global measures indicate that this translates into individual search trajectories rapidly getting stuck in local minima in the energy landscape; no more than a few distinct structural states are explored during any run. This behavior is very likely to contribute to the poor performance of fragment‐based techniques in *de novo* prediction of larger targets.

In the case of Rosetta, we find that the commonly‐employed strategy of a larger number of short prediction runs is beneficial because of the improved degree of conformational exploration it effectively affords, using a given amount of computational effort. Our methods can be used in a similar manner to evaluate the effects of different settings of other parameters on the quality of conformational sampling.

In the case of EdaFold, we find that the use of probability distributions during the search has two distinct but related effects: realizing more balanced sampling between structurally distinct states, followed by an increasingly focused search in low‐energy conformational subspaces. Drawing a distinction between these effects would not be possible using conventional measures of prediction performance.

Diagnosing the weaknesses of current methods of conformational sampling in fragment assembly is, of course, only a first step toward addressing the problem. Our analysis highlights possible avenues toward the improvement of low‐resolution search, in particular the need to develop protocols that focus conformational search on key degrees of freedom such as loop regions of the proteins. Designed correctly, such methods should be able to more systematically explore a diverse set of “protein‐like” conformations in a single search trajectory. Sampling approaches that are capable of improved conformational exploration are necessary if fragment‐based methods are to scale to larger targets, and our methods can be used to support their design and assessment, as they support the identification of deficiencies, the quantification of exploration, and an objective analysis of the impact of parameter settings and algorithm redesigns.

## Supporting information

Supporting Information

Supporting Information

Supporting Information
