# Supplementary material for: Toward a detailed understanding of search trajectories in fragment assembly approaches to protein structure prediction
Source: Proteins. 2016 Feb 23;84(4):411–26. doi: 10.1002/prot.24987 (PMC4982100; doi:10.1002/prot.24987)
Supplement: Supplementary file 1 — Supporting Information [file PROT-84-411-s001.pdf]

List of Supplementary material.

Supplementary table S1. First-percentile conditions on the JmjC-proteins.

Supplementary table S2. Family classification of JmjC domains using the first-percentile conditions.

Supplementary table S3. Family classification of JmjC domains when using the second-percentile conditions.

Supplementary figure S4. Active-site organization in a hypothetical PHF2 protein.

Supplementary figure S5. Proposed docking of HIS3-Arg2Me_2-Lys4Me_2 to JmjD6.

Supplementary table S1. List of first-percentile conditions on the JmjC domains. Conditions are ordered by their corresponding sequence position.

4:F	76:D
4:M	82:Q
4:Q	84:F
6:I	97:Q
8:K	123:W
8:S	172:W
10:C	200:N
10:W	217:H
11:K	228:Y
13:R	230:C
13:Y	230:G
17:D	230:T
21:L	231:I
23:F	233:W
23:N	241:T
23:P	242:P
23:V	250:V
24:F	251:F
24:G	252:G
24:M	252:N
24:Q	252:T
27:L	253:G
44:S	253:H
48:N	253:Q
51:N	253:W
59:G	255:W
70:P	

'First-percentile conditions' are all alignment position:residue pairs which had at least one relative entropy value among the first percent of ranked coupling values when considering all the conditions with at least 25 occurencies and their coupling (Equation 1) to all observation positions (see METHODS for details).

Supplementary Table S2. Family classification of JmjC domains obtained using the first-percentile conditions.

I	II	III	IV	V
A1C5Y8_ASPC	A0NF16_ANOG	A0BZ39_PARTE	A0C6J8_PARTE	A0BYP4_PARTE
A1UW08_BURM	A4HFP0_LEIB	A0C6E9_PARTE	A0CT09_PARTE	A0CNN6_PARTE
A3GH35_PICST	A4I2R6_LEII	A1C4Z5_ASPC	A0DHI7_PARTE	A0DIL9_PART
A4S6R8_OSTL	A7SG66_NEMV	A1CPM9_ASPC	A0DV21_PARTE	A1CJT6_ASPC
A5D9U8_PICG	A7SQQ9_NEMV	A2Y2Y1_ORYS	A1D6P5_NEOF	A2XHI8_ORYSI
A5DT38_LODE	A8J9A8_CHLR	A3GGH3_PICST	A2QHK3_ASPN	A2ZFJ5_ORYSI
A5PL83_DANRE	A8P5K4_BRUM	A4R154_MAGG	A2XCL2_ORYSI	A4HFQ9_LEIB
A7S527_NEMV	A8WL35_CAEBR	A4RUM1_OSTL	A3LST7_PICST	A4QVP1_MAGG
A7S9X9_NEMV	A9VCA5_MONB	A5DIU8_PICG	A4RD83_MAGG	A4RUR8_OSTL
A7ZAN0_BACA	B0WCR3_CULQ	A5E3H8_LODE	A4S3A2_OSTL	A4SA00_OSTL
A8KBF7_XENT	B3MLU9_DROA	A5WUR6_DANRE	A5DDN2_PICG	A5BXV5_VITVI
A8Q9E4_MALG	B3NMC6_DROE	A6QWG4_AJEC	A5E3W2_LODE	A5E127_LODE
A8UNR7_9FLA	B3PGK9_CELJ	A6RQG3_BOTF	A6ZQ75_YEAS	A6RJ46_BOTF
A8WQZ7_CAEBR	B4G990_DROP	A6ZRC5_YEAS	A7PBA6_VITVI	A7NUV3_VITVI
A8XW64_CAEBR	B4JDK2_DROG	A6ZY60_YEAS	A7PC31_VITVI	A7S5J7_NEMV
A9USV7_MONB	B4MWV2_DROW	A7QC14_VITV	A7PCK1_VITVI	A8IL16_CHLR
B0D233_LACB	B6K2H3_SCHJ	A7QMV3_VITV	A7PI24_VITVI	A8J7U9_CHLR
B0WMD3_CULQ	B7FSM7_PHAT	A7SN37_NEMV	A7SA95_NEMV	A8Q987_MALG
B2VTX3_PYRT	B7G4A7_PHAT	A8IAF5_CHLR	A8P7E1_COPC	A9SHT7_PHYP
B3RIC2_TRIA	B8A9Z6_ORYS	A8P2R1_COPC	A8PBD3_BRUM	A9V448_MONB
B3RJX0_TRIA	B8JMH1_DANRE	A8PQK2_BRUM	A8WNJ7_CAEBR	B0XKB9_CULQ
B6D5Q0_PERE	B9RAY2_RICC	A8Q2Y9_MALG	A8WZL5_CAEBR	B2AYA5_PODA
B6K4V0_SCHJ	C1E8E7_9CHL	A8Q5U8_BRUM	A8X4Q8_CAEBR	B2WCP0_PYRT
B7FNV3_PHAT	C1MS37_9CHL	A8XYB2_CAEBR	A8XNY3_CAEBR	B3LVN8_DROA
B7P7A8_IXOS	C4QA79_SCHM	A9SW94_PHYP	A8XQN7_CAEBR	B3RKJ2_TRIA
B7PLW8_IXOS	JMJ1_SCHPO	A9V5U7_MONB	A8Y2X0_CAEBR	B6HH81_PENC
B9EKK1_M	JMJD4_CHICK	B0D916_LACB	A8Y2X1_CAEBR	B6KBT5_TOXG
B9JNX0_AGRR	JMJD4	B0WPN8_CULQ	A8Y4T7_CAEBR	B6KMR4_TOXG
B9WKR9_CAND	JMJD4_M	B0WVT3_CULQ	A9SYV0_PHYP	B7G028_PHAT
C1E2V0_9CHL	Q14V35_CAEEL	B0XJL2_CULQ	A9U241_PHYP	B7G0J1_PHAT
C1GR66_PARB	Q16JP5_AEDA	B2W2M6_PYRT	B0CSV7_LACB	B7G2F5_PHAT
C1MMS0_9CHL	Q386X8_9TRY	B3MDS1_DROA	B0DK67_LACB	B7Q2P5_IXOS
C1N979_9CHL	Q4CXU5_TRYC	B3MGA5_DROA	B0XFK2_CULQ	B8C8F0_THAP
C3RZA6_PIG	Q4PHR0_USTM	B3N5A4_DROE	B0XXA6_ASPF	B9SDN5_RICC
C3YVE1_BRAF	Q5DAY2_SCHJ	B3RV05_TRIA	B2B0D8_PODA	C0S1S0_PARB
C4PZM5_SCHM	Q5KEK3_CRYN	B3S1P1_TRIA	B2W0Z1_PYRT	C1E307_9CHL
C4QWP8_PICPA	Q67ZB6_ARATH	B4FIH5_MAIZE	B3MPP9_DROA	C1N9F4_9CHL
C4Y832_CLAL		B6K3U5_SCHJ	B3RKJ9_TRIA	C3ZFA3_BRAF
C5M4L4_CANT		B7G607_PHAT	B3RNT4_TRIA	C4JZT9_9EUR
JHD1_CRYNE		B7QJG8_IXOS	B3RUC5_TRIA	C4Q4S0_SCHM
JHD1_DEBHA		B8AYX5_ORYS	B3RZL7_TRIA	C4R3C6_PICPA
JHD1_SCHPO		B8BTH4_THAP	B6HF34_PENC	C4Y3Y3_CLAL
JHD1_YARLI		B8C156_THAP	B6KVK1_TOXG	C5E3D8_LACT
JMJCA_DICDI		B8M2H8_TALS	B6Q2P2_PENM	C5FTX0_MICC
KDM7_CAEEL		B9I0V1_POPT	B7G887_PHAT	C5KIQ4_9ALV

KDM7A
PHF2
PHF8_M
Q00WT0_OSTT
Q1RQ05_CIOI
Q20367_CAEEL
Q2SGN5_HAHC
Q4S3J1_TETN
Q4SAD2_TETN
Q4SJB3_TETN
Q4T9B3_TETN

B9I5N6_POPT
B9WFV6_CAND
C0SUT9_ARAT
C1DZP9_9CHL
C1EC12_9CHL
C4Q200_SCHM
C4QZI9_PICPA
C4Y9F3_CLAL
C5DN62_LACT
C5DRW0_ZYGR
C5FEP0_MICC
C5K916_9ALV
C5M365_CANT
JMJ2_SCHPO
KDM4C_DANRE
KDM4D
KDM4D_RAT
O64752_ARATH
Q1RLC8_CIOI
Q2V3G7_ARAT
Q4P364_USTM
Q4SBP1_TETN
Q4SFG0_TETN
Q4STB9_TETN
Q55IR5_CRYN
Q6BP72_DEBH
Q6C1P7_YARL
Q6CSU8_KLUL
Q6FKG0_CANG
Q74ZT1_ASHG
Q8W4M0_ARATH

B7QEK5_IXOS
B8A7U6_ORYS
B8AHB4_ORYS
B8BIE7_ORYS
B8BP43_ORYS
B8BUQ0_THAP
B9H203_POPT
B9HYN6_POPT
B9IDQ7_POPT
B9S226_RICC
B9SFD1_RICC
C0NID7_AJEC
C0S5X0_PARB
C0YJF1_9FLA
C1E8J6_9CHL
C1FDF8_9CHL
C1MLP7_9CHL
C3ZDP8_BRAF
C4JF64_9EUR
C4Q2A1_SCHM
C4Q6I3_SCHM
C4XWF4_CLAL
C5FJ14_MICC
C5M237_CANT
KDM6A_DANRE
KDM6A_M
KDM6B_RAT
O17619_CAEEL
O57435_FUGR
Q09519_CAEEL
Q0CHL0_ASPT
Q19565_CAEEL
Q19760_CAEEL
Q22T48_TETT
Q4UG30_THEA
Q55ER4_DICD
Q55WZ6_CRYN
Q5B574_EMEN
Q6BDA0_ARATH
Q6BME1_DEBH
Q9FJS0_ARATH

FB92_ARATH
JMJD6_MOUSE
PTDSR_CAEBR
PTDSR_HYDAT
Q386V9_9TRY
Q4E398_TRYC
Q4PGG4_USTM
Q55CL5_DICD
Q6CK82_KLUL
Q75ES6_ASHG

Proteins are represented by their Uniprot names. Uniprot names were replaced by their equivalent usual names for common enzymes. M is for MOUSE, and no species indicates a human protein.

Supplementary table S3. Family classification of JmjC domains obtained using the second-percentile conditions.

I	II	III	IV	V	VI
A0BYP4_PART	A0DHI7_PART	A1C5Y8_ASPCL	A0BZ39_PART	A0NF16_ANOGA	A0JM02_XTr
A0C6J8_PART	A0DV21_PART	A2QHK3_ASPNC	A0C6E9_PART	A4HFP0_LEIBR	A1C4Z5_ASPCL
A0CNN6_PART	A4S3A2_OSTL	A3GH35_PICST	A1CPM9_ASPCL	A4I2R6_LEIIN	A2XCL2_ORYS
A0CT09_PART	A7AV25_BABBO	A4S6R8_OSTL	A3GGH3_PICST	A7QF33_VITV	A2Y2Y1_ORYS
A0DIL9_PART	A7SA95_NEMV	A5D9U8_PICGU	A4R154_MAGG	A7SG66_NEMV	A3LST7_PICST
A1CJT6_ASPCL	A8P7E1_COPC7	A5DT38_LODE	A5DIU8_PICGU	A8J9A8_CHLRE	A4QU39_MAGG
A1UW08_BURM	A8PBD3_BRUM	A7RJK6_NEMV	A5E3H8_LODE	A8P5K4_BRUM	A4S152_OSTL
A2XHI8_ORYS	A8WNJ7_CAEB	A7S527_NEMV	A6QWG4_AJECN	A8WL35_CAEB	A5DDN2_PICGU
A2ZFJ5_ORYS	A8WZL5_CAEB	A7S9X9_NEMV	A6RQG3_BOTFB	A9V5R2_MONB	A5E3W2_LODE
A4HFQ9_LEIB	A8X4Q8_CAEB	A8KBF7_XTr	A6ZRC5_YEAS7	A9VCA5_MONB	A5WUR6_DANR
A4QNS2_XL	A8XNY3_CAEB	A8Q9E4_MALG	A6ZY60_YEAS7	B0WCR3_CULQU	A6ZQ75_YEAS
A4QVP1_MAGG	A8XQN7_CAEB	A8UNR7_9FLAO	A8IAF5_CHLRE	B2ABN8_PODAN	A7PBA6_VITV
A4RUR8_OSTL	A8Y2X0_CAEB	A8WQZ7_CAEB	A8IR59_CHLRE	B3MLU9_DROA	A7PC31_VITV
A4SA00_OSTL	A8Y2X1_CAEB	A8XW64_CAEB	A8P2R1_COPC7	B3NMC6_DROE	A7PCK1_VITV
A5BXV5_VITV	A8Y4T7_CAEB	A9USV7_MONB	A8Q2Y9_MALGO	B4G990_DROPE	A7PI24_VITV
A5E127_LODE	B0XFK2_CULQU	A9V1D0_MONB	A8Q5U8_BRUM	B4JDK2_DROGR	A7QC14_VITV
A6RJ46_BOTFB	B0XXA6_ASPFC	B0CSV7_LACBS	A8XYB2_CAEB	B4MWV2_DROWI	A7QMV3_VITV
A7NUV3_VITV	B2BL36_RAT	B0D233_LACBS	A9V5U7_MONB	B6K2H3_SCHJY	A7SN37_NEMV
A7S5J7_NEMV	B3MPP9_DROA	B0WMD3_CULQ	B0D916_LACBS	B7FSM7_PHATR	A8NYS5_COPC7
A7ZAN0_BACA	B3RUC5_TRIAD	B2VTX3_PYRTR	B0WVT3_CULQU	B8A9Z6_ORYS	A8PQK2_BRUM
A8IL16_CHLR	B6KVK1_TOXGO	B3RIC2_TRIAD	B0XJL2_CULQU	B8JMH1_DANRE	A9SW94_PHYP
A8J7U9_CHLRE	B7QEK5_IXOSC	B3RJX0_TRIAD	B2W2M6_PYRTR	B9RAY2_RICCO	A9SYV0_PHYP
A8Q987_MALGO	B9H203_POPTR	B6D5Q0_PERER	B2W5K4_PYRTR	C1E8E7_9CHLO	A9TI08_PHYP
A9SHT7_PHYP	B9HYN6_POPTR	B6HF34_PENCW	B3MDS1_DROA	C1MS37_9CHLO	A9UXW9_MONB
A9V448_MONB	B9SFD1_RICCO	B6K4V0_SCHJY	B3MGA5_DROA	C4QA79_SCHMA	B0WPN8_CULQ
B0XKB9_CULQU	C1FDF8_9CHLO	B6Q2P2_PENM	B3S1P1_TRIAD	C4QGS9_SCHMA	B2WM33_PYRT
B2AYA5_PODA	C1MLP7_9CHLO	B7FNV3_PHATR	B7G607_PHATR	JMJ1_SCHPO	B3N5A4_DROE
B2WCP0_PYRTR	C3ZDP8_BRAFL	B7P7A8_IXOSC	B8C156_THAPS	JMJD4_CHICK	B3RV05_TRIAD
B3LVN8_DROA	C4JF64_9EURO	B7PLW8_IXOS	B8M2H8_TALSN	JMJD4	B4FIH5_MAIZE
B3RKJ2_TRIAD	C4Q6I3_SCHMA	B9EKK1_M	B9WFV6_CANDC	JMJD4_M	B6K3U5_SCHJY
B3RKJ9_TRIA	C5FJ14_MICCA	B9WKR9_CANDC	C1EC12_9CHLO	O97101_DICDI	B7QJG8_IXOSC
B6HH81_PENC	KDM6A_DANRE	C0NID7_AJECG	C4Q200_SCHMA	Q14V35_CAEEL	B8A7U6_ORYS
B6KBT5_TOXGO	KDM6A_M	C1GR66_PARBR	C4QZI9_PICPA	Q16JP5_AEDAE	B8AHB4_ORYS
B6KMR4_TOXG	O17619_CAEEL	C1N979_9CHLO	C4Y9F3_CLALS	Q386X8_9TRYP	B8AYX5_ORYS
B7G028_PHAT	O57435_FUGRU	C3RZA6_PIG	C5DN62_LACTH	Q4CXU5_TRYCR	B8BIE7_ORYS
B7G0J1_PHAT	Q09519_CAEEL	C3YVE1_BRAFL	C5DRW0_ZYGRO	Q4PHR0_USTMA	B8BP43_ORYS
B7G2F5_PHATR	Q19565_CAEEL	C3ZGY9_BRAFL	C5FEP0_MICCA	Q5DAY2_SCHJA	B8PMU6_POSP
B7G4A7_PHAT	Q19760_CAEEL	C4PZM5_SCHMA	C5K916_9ALVE	Q5KEK3_CRYNE	B9FR15_ORYSJ
B7Q2P5_IXOSC	Q22T48_TETTH	C4QWP8_PICPA	C5M365_CANTR	Q67ZB6_AT	B9GWH9_POPTR
B8C8F0_THAPS	Q4UG30_THEAN	C4Y832_CLALS	KDM4C_DANR		B9I0V1_POPTR
B9SDN5_RICCO		C5M4L4_CANTR	KDM4D		B9I5N6_POPTR
C0H2H9_THINE		JHD1_CRYNE	KDM4D_RAT		B9RCC0_RICCO
C0S1S0_PARB		JHD1_DEBHA	Q4P364_USTMA		B9S226_RICCO
C1E307_9CHLO		JHD1_SCHPO	Q4SBP1_TETNG		C0SUT9_AT
C1N9F4_9CHLO		JHD1_YARLI	Q4SFG0_TETNG		C1E1H6_9CHLO
C3ZFA3_BRAF		KDM7_CAEEL	Q55IR5_CRYNE		C1MK28_9CHLO

C4JZT9_9EURO		KDM7A_DANR	Q6BP72_DEBH		C4Q2A1_SCHMA
C4Q4S0_SCHMA		KDM7A	Q6C1P7_YARLI		C4QEF1_SCHMA
C4R3C6_PICPA		PHF2	Q6CR98_KLUL		C4R2N4_PICPA
C4Y3Y3_CLALS		PHF8_M	Q6CSU8_KLUL		C4XWF4_CLALS
C5E3D8_LACTH		Q00WT0_OSTTA	Q6FKG0_CANGA		C5M237_CANTR
C5FTX0_MICCA		Q010Q7_OSTTA	Q74ZT1_ASHGO		JMJ2_SCHPO
C5KIQ4_9ALVE		Q1RQ05_CIOIN	Q75D56_ASHGO		KDM5_CAEB
FB92_AT		Q20367_CAEEL	Q7SE87_NEUCR		O64752_AT
JMJD6_M		Q2SGN5_HAHCH			Q1RLC8_CIOIN
PTDSR_CAEB		Q4S3J1_TETN			Q2GWJ9_CHAGB
PTDSR_HYDAT		Q4SAD2_TETN			Q2V3G7_AT
Q386V9_9TRYP		Q4SJB3_TETN			Q4PBD1_USTMA
Q4E398_TRYCR		Q4T9B3_TETN			Q4STB9_TETN
Q4PGG4_USTMA					Q55ER4_DICDI
Q55CL5_DICDI					Q5KHN4_CRYNE
Q6CK82_KLULA					Q6BDA0_AT
Q75ES6_ASHGO					Q6BME1_DEBH
Q7N1F0_PHOL					Q8W4M0_AT
					Q94BQ7_AT
					Q9FJS0_AT

Clustering of the conditions and family assignment was performed according to the protocol described in METHODS for the first-percentile conditions, except that the second-percentile conditions were used. Proteins are represented by their Uniprot names. Species where indicated as four letters for the sake of space when not ambiguous, M stands for MOUSE and default is HUMAN.

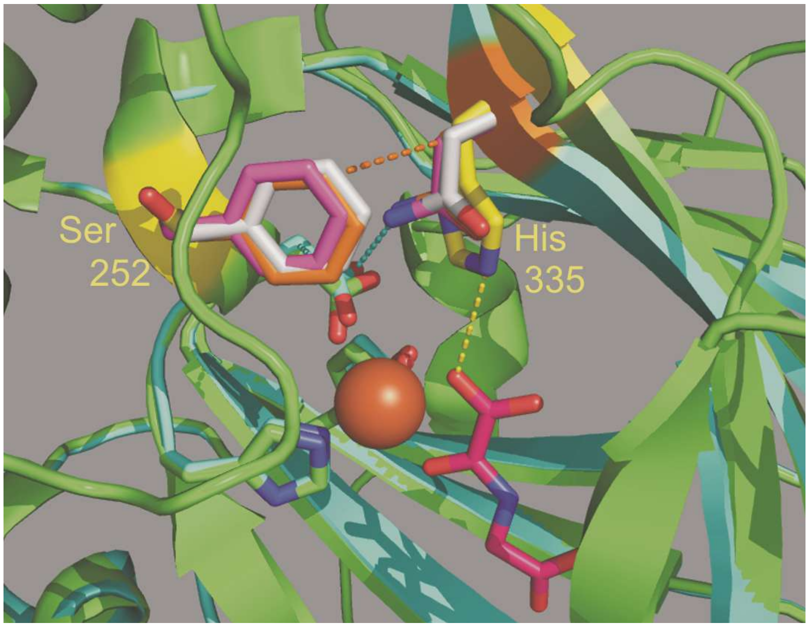

Supplementary figure S4. Active-site organization in a hypothetical PHF2 protein. The original PHF2 structure is shown in green, with iron as an orange sphere and mutated residues (Ser252, position 23 and His335, position 254) in yellow. The first predicted mutated structure is shown in cyan, and the mutated residues are shown for the three predicted structures as orange, pink and gray sticks, respectively. 2-Oxoglutarate was inserted according to the crystallized structure for PHF8 for the iron-docking part and by superimposition to crystallized acetate for its distal carboxylate. Possible interactions of His335 to oxoglutarate (3.58 Å, yellow dashes) is shown for the native structure, as well as those of Asn* to Asp251 (4.03 Å, cyan dashes) and of C_β of Asn* to Phe* (4.11 Å, orange dashes) in the first predicted mutant structure.

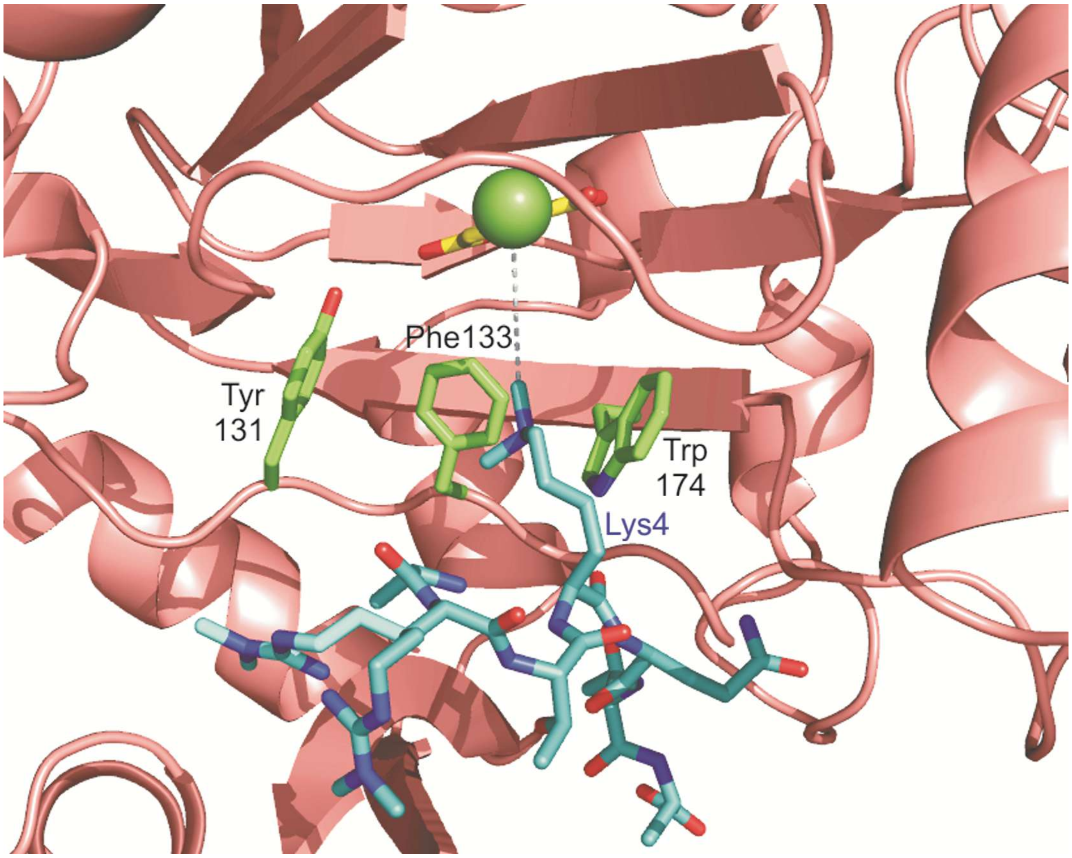

Supplementary figure S5. Proposed docking of HIS3-Arg2Me$_2$-Lys4Me$_2$ to JmjD6. JmjD6 is shown in pink. The active site nickel ion is shown as a green sphere. The two first poses are shown for the docked peptide, with carbon atoms respectively in pale cyan and cyan. Only Arg2 shows different positioning in the two poses (bottom left of the image). 2-Oxoglutarate was placed by superposing its distal carboxylic group to the crystallized acetate and its 2-oxocarboxylic end to the coordination site with iron observed in PHF8.
