# Supplementary material for: Toward a detailed understanding of search trajectories in fragment assembly approaches to protein structure prediction
Source: Proteins. 2016 Feb 23;84(4):411–26. doi: 10.1002/prot.24987 (PMC4982100; doi:10.1002/prot.24987)
Supplement: Supplementary file 2 — Supporting Information [file PROT-84-411-s002.pdf]

# Data Supplement

## Local Measures, MDS results and difficulty of prediction for all targets

This document contains plots of move acceptance frequency, proportion of unique torsion angle triplets explored, and MDS plots for each of the 59 targets considered in this manuscript, for each running protocol considered. Targets are presented in the same order as in supplementary table S1, with the exception of 1ail, which appears at the end. For descriptions of the different plots, please refer to the main text. The title gives the PDB identifier of the target, along with its secondary structure class as assigned by SCOP [1, 2] version 1.75, and sequence length. Note that the depiction of secondary structure beneath the plots of local measures (see Methods section in main text) are generated by PSIPRED [3] and so may differ from the SCOP annotation.

Beneath each MDS plot, we include a measure $f$ of the percentage of total variance in the dataset captured by the first two principal coordinates. This is calculated as

$$f = \frac{\lambda_1 + \lambda_2}{\sum_{i=1}^{n} |\lambda_i|} \times 100, \quad \text{where}$$

each $\lambda_i$ is an eigenvalue (sorted in descending order) of the doubly centred distance matrix calculated from the input distances during the MDS procedure, and
$n$ is the number of data points in the input.

Following this, histograms of RMSD values are given. These histograms show the distribution of backbone RMSD values when considering 1000 decoys obtained from short Rosetta runs and EdaFold runs (after all-atom refinement), and is intended to provide an indication of the difficulty of obtaining accurate predictions for each protein target: a large fraction of structures with low RMSD values indicates that a target is relatively “easy” to predict. The decoy set from EdaFold is a random sample of 1000 decoys obtained from multiple replicate runs using the parameters given in the main text, and contains structures obtained at the end of the fourth round of the iterative procedure.

# 1enh ( all-$\alpha$, 54 residues )

Sets of short Rosetta Runs | Long Rosetta Runs | EdaFoldAA

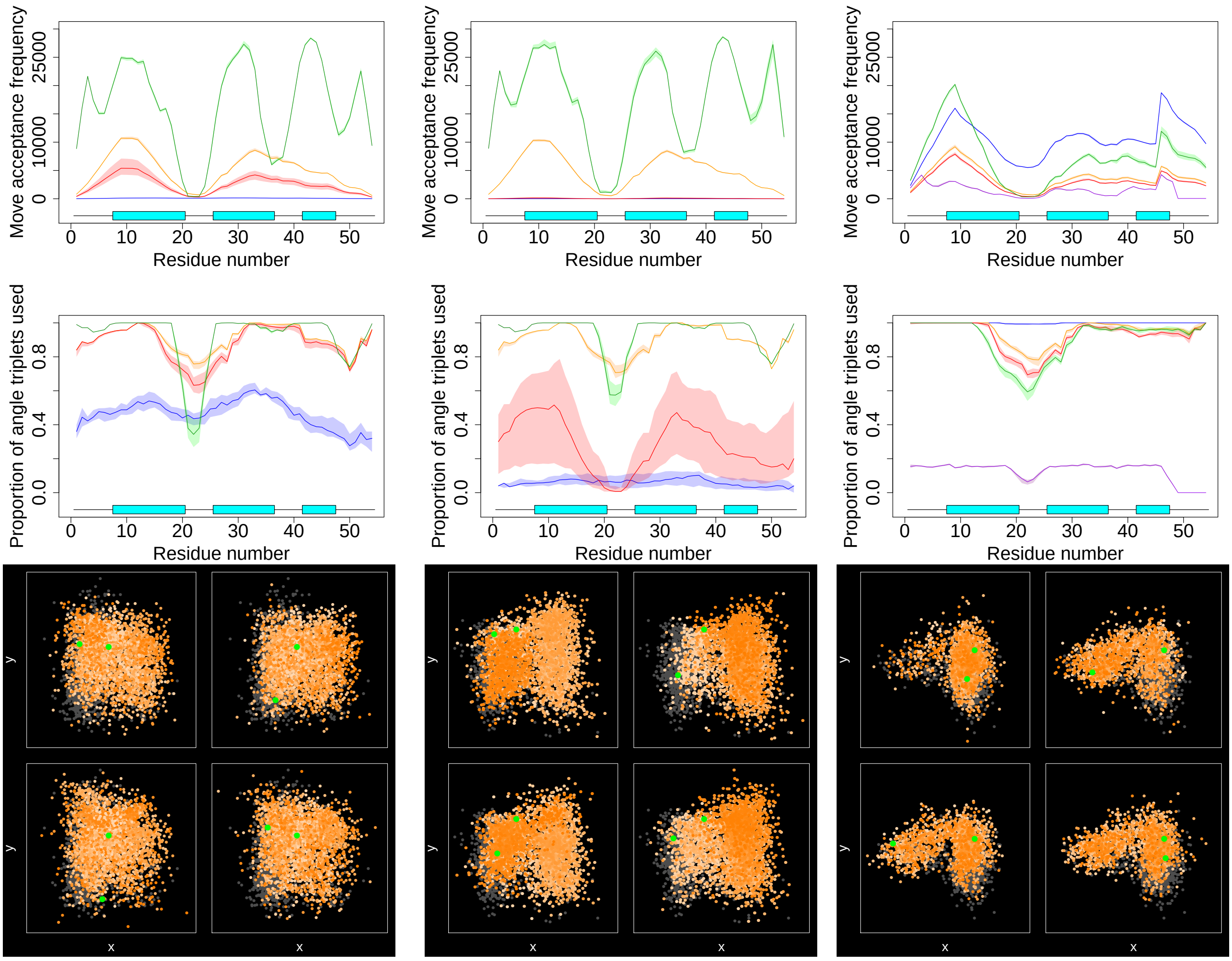


$f$ = 11.186 | $f$ = 12.369 | $f$ = 12.104

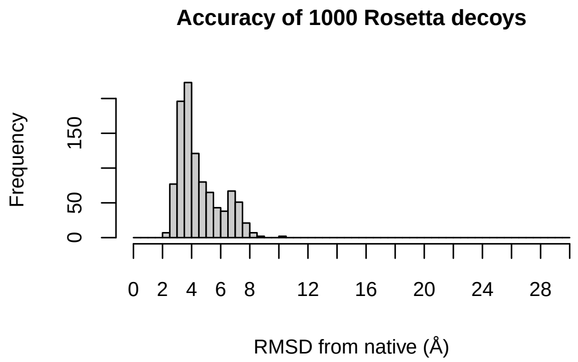


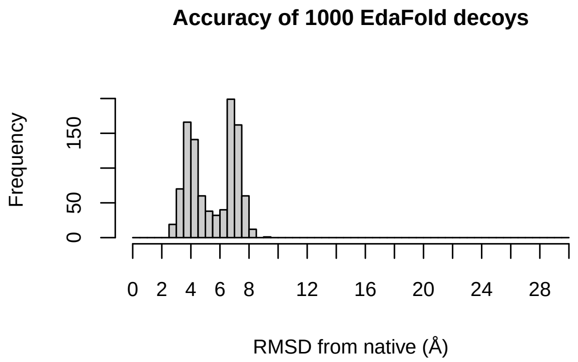

# 1r69 ( all-$\alpha$, 63 residues )

Sets of short Rosetta Runs | Long Rosetta Runs | EdaFoldAA

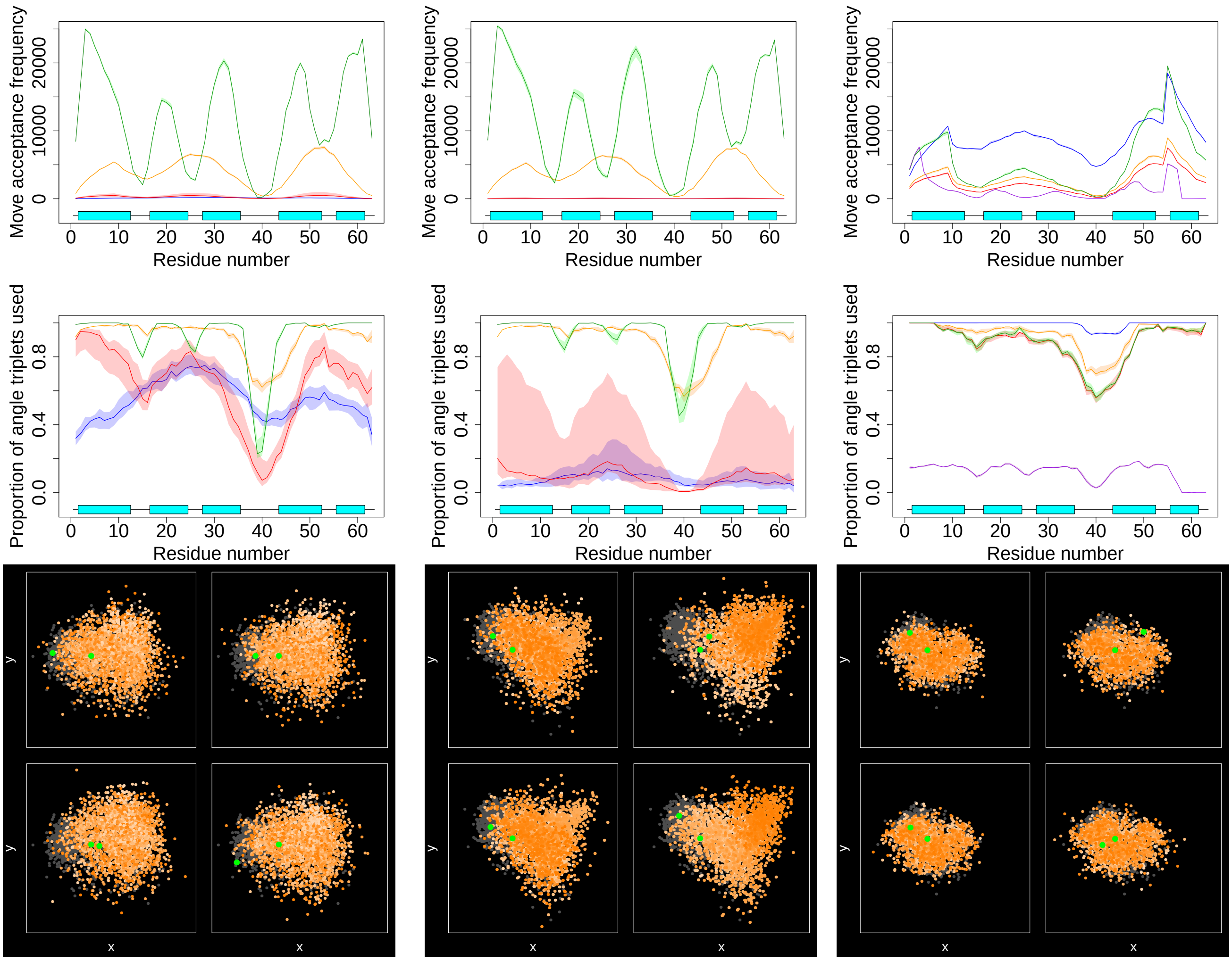


$f$ = 11.442 | $f$ = 11.305 | $f$ = 15.263

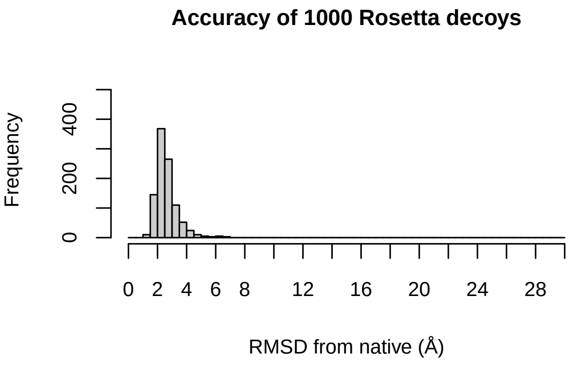


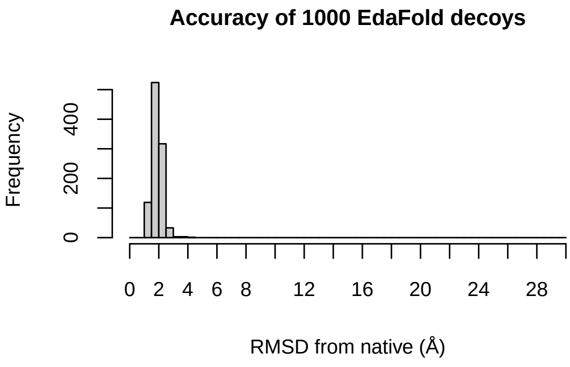

## 1a32 ( all-$\alpha$, 65 residues )

Sets of short Rosetta Runs | Long Rosetta Runs | EdaFoldAA

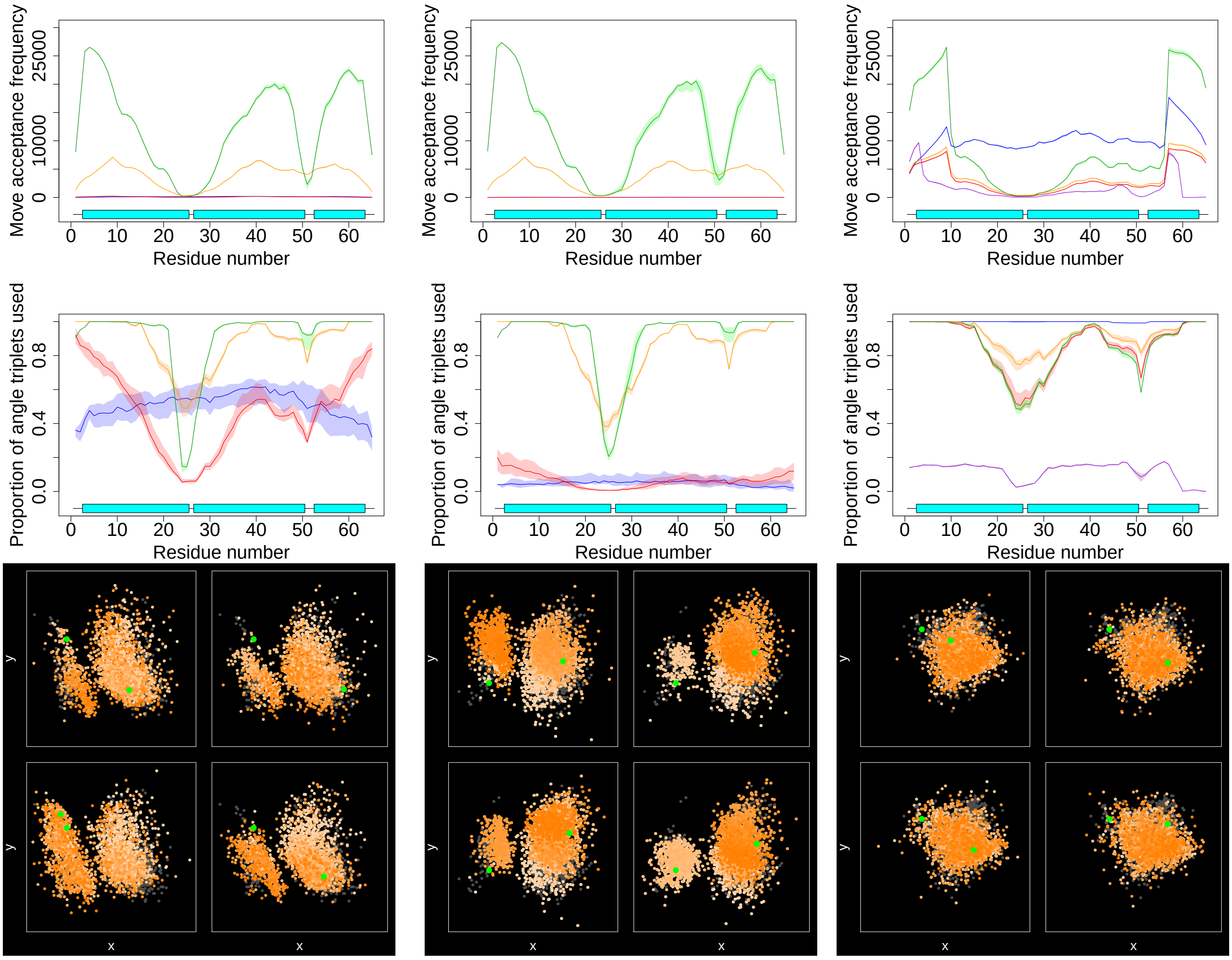


$f$ = 13.907 | $f$ = 14.969 | $f$ = 19.498

**Accuracy of 1000 Rosetta decoys**

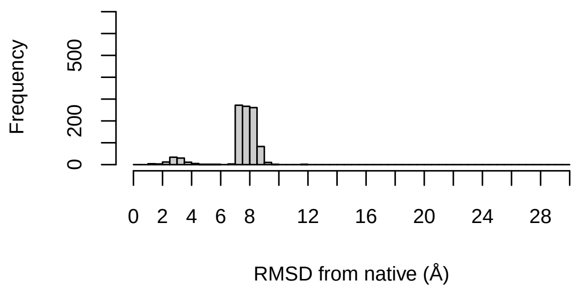


**Accuracy of 1000 EdaFold decoys**

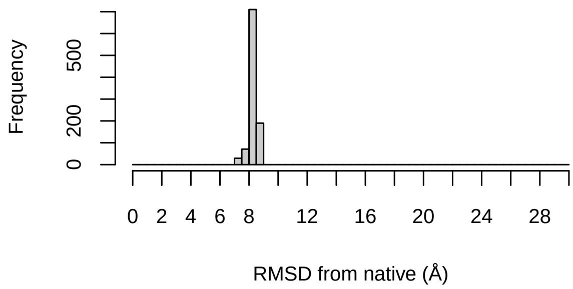

# 1utg ( all-$\alpha$, 70 residues )

Sets of short Rosetta Runs | Long Rosetta Runs | EdaFoldAA

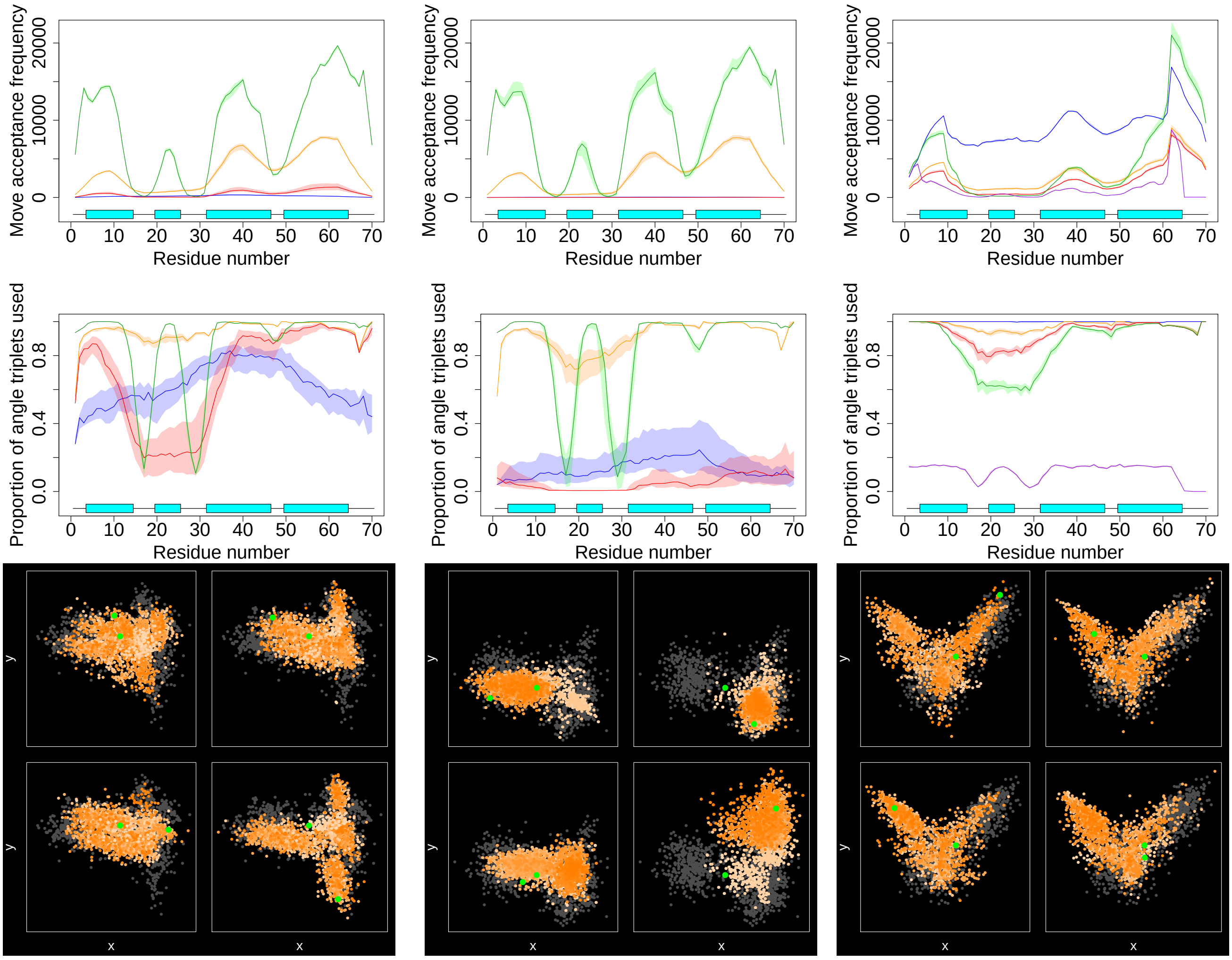


$f$ = 10.001 | $f$ = 12.411 | $f$ = 12.898

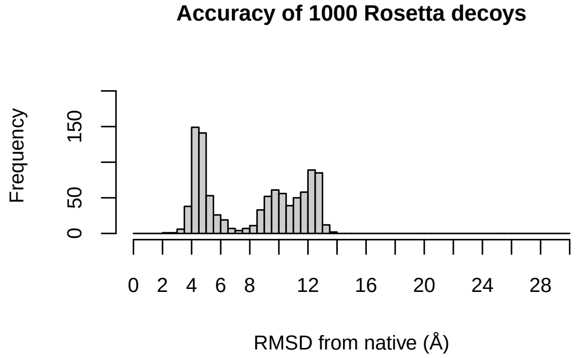


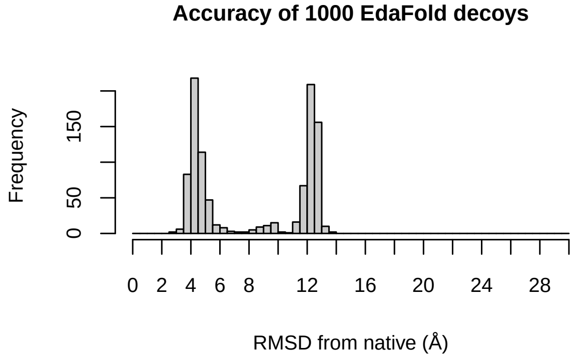

# 1ig5A ( all-$\alpha$, 75 residues )

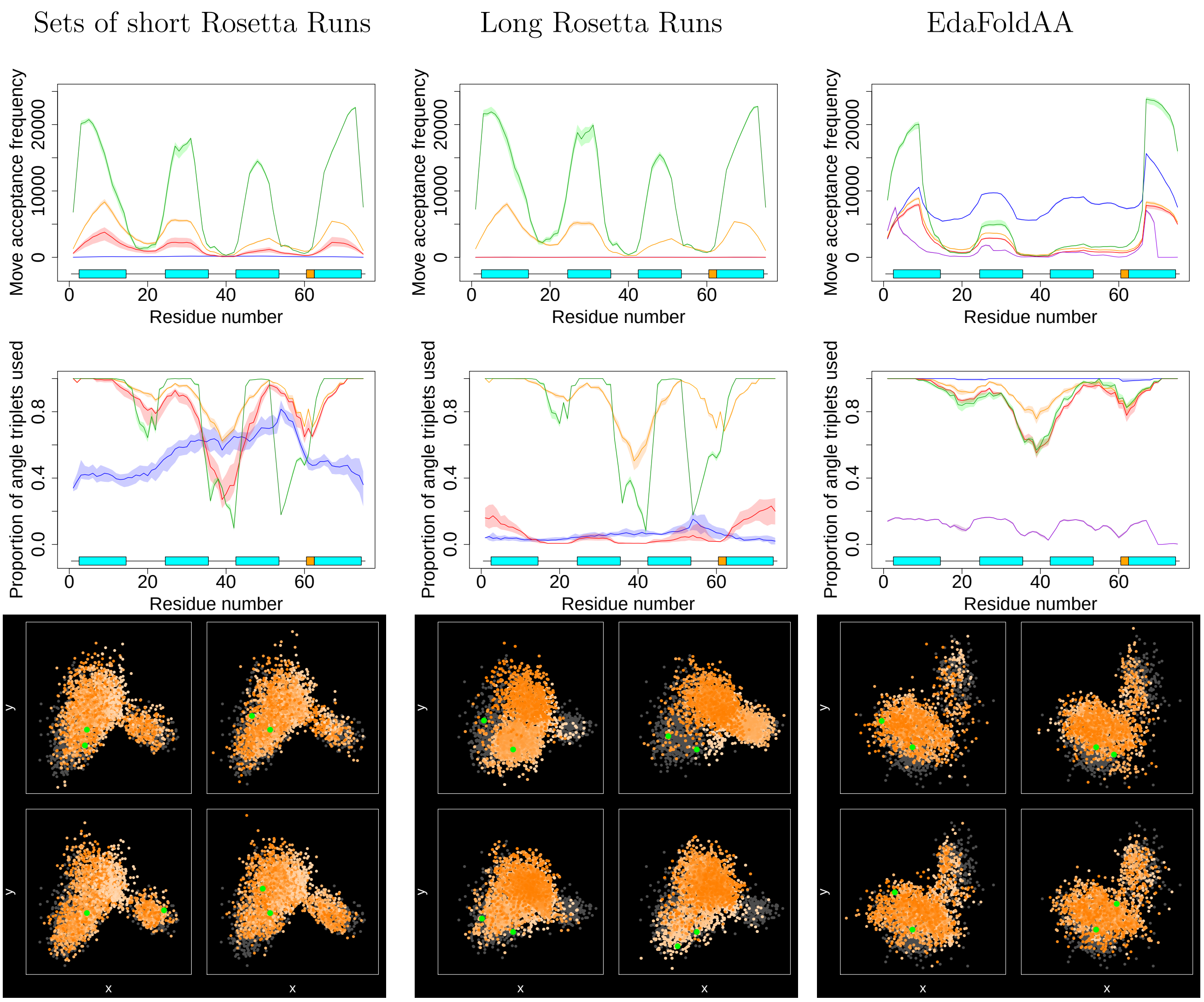


$f$ = 10.028 $f$ = 8.8922 $f$ = 9.2093

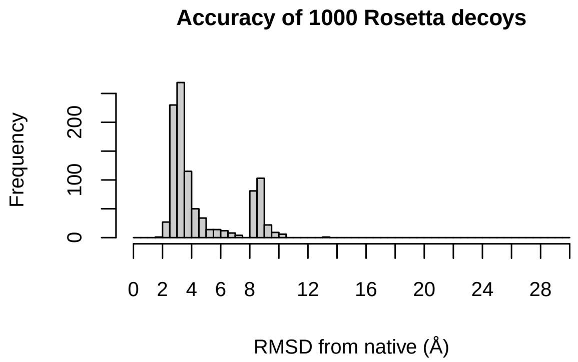


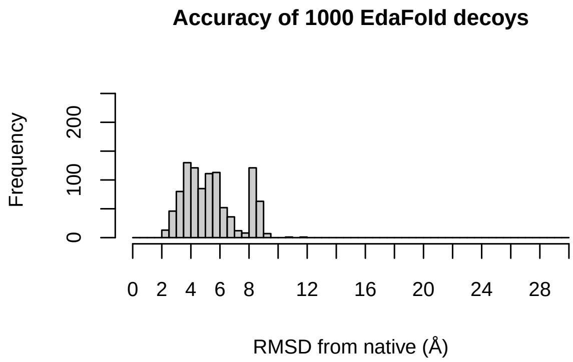

# 1cei ( all-$\alpha$, 85 residues )

Sets of short Rosetta Runs | Long Rosetta Runs | EdaFoldAA

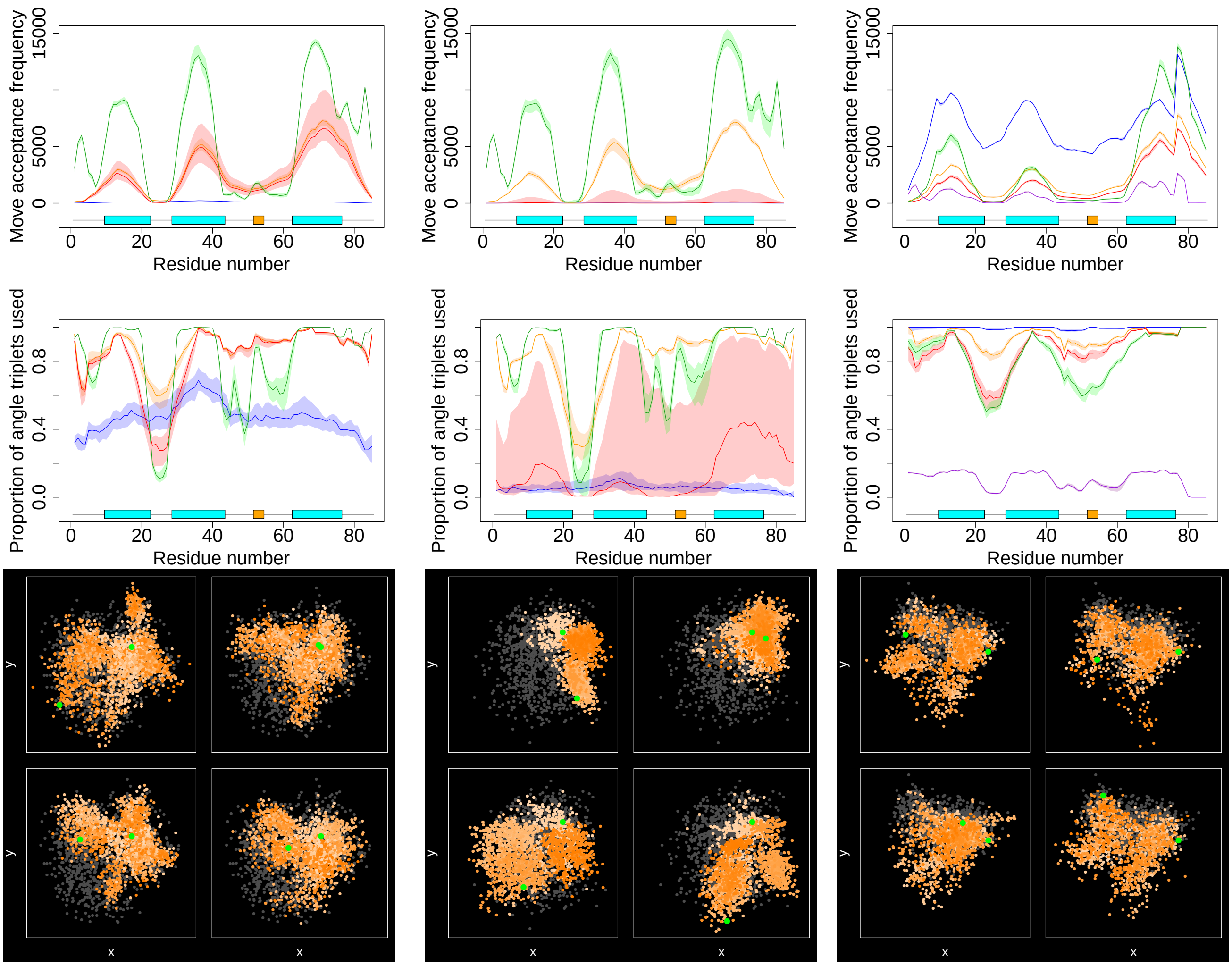


$f$ = 5.5184 | $f$ = 7.9817 | $f$ = 6.9501

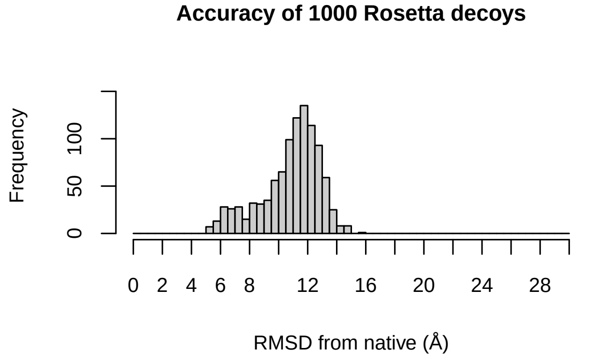


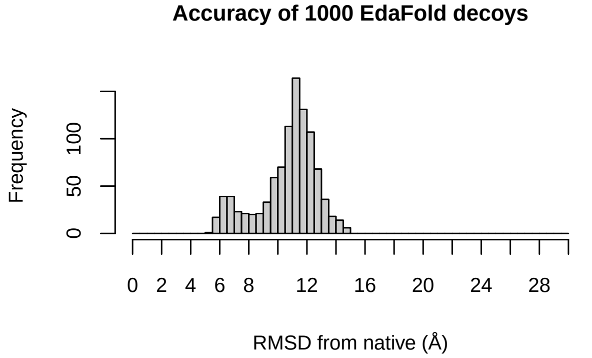

# 256bA ( all-$\alpha$, 106 residues )

Sets of short Rosetta Runs | Long Rosetta Runs | EdaFoldAA

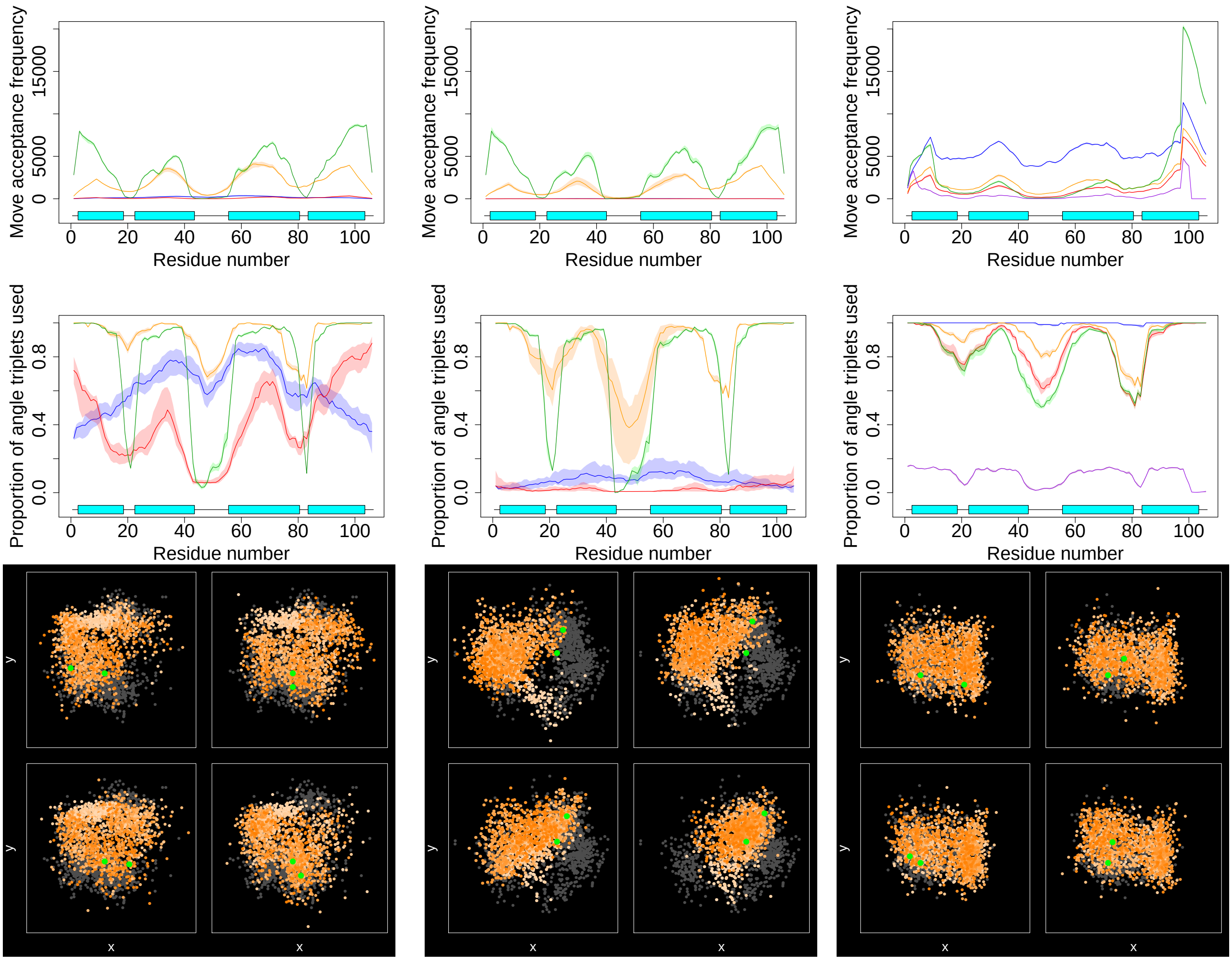


$f = 11.54$ | $f = 11.182$ | $f = 12.982$

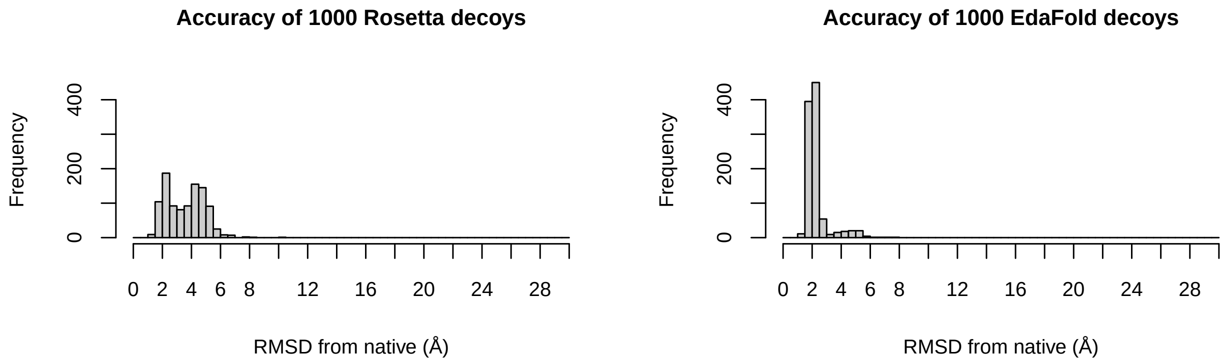

# 1bkrA ( all-$\alpha$, 108 residues )

Sets of short Rosetta Runs | Long Rosetta Runs | EdaFoldAA

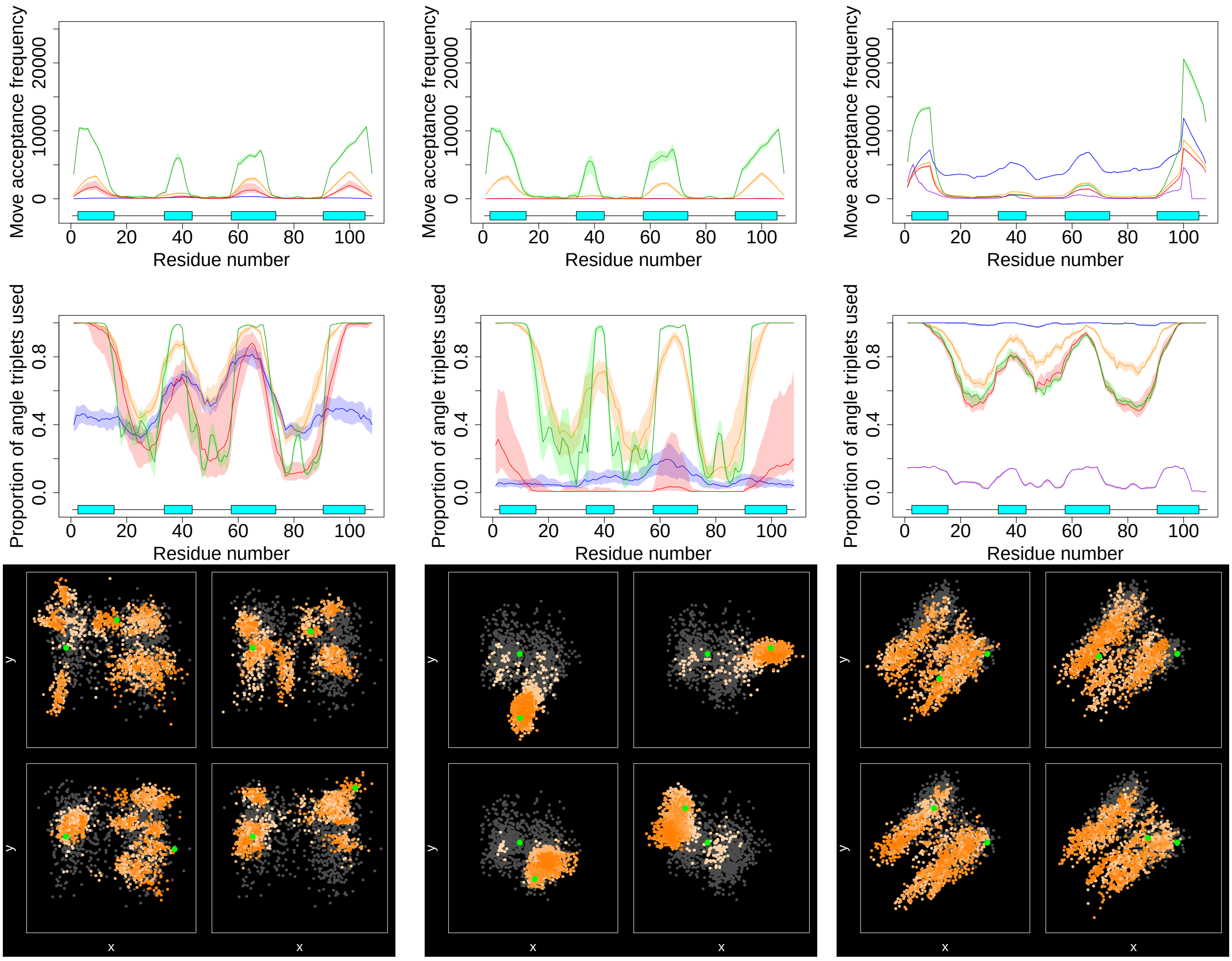


$f = 6.923$ | $f = 10.069$ | $f = 8.1618$

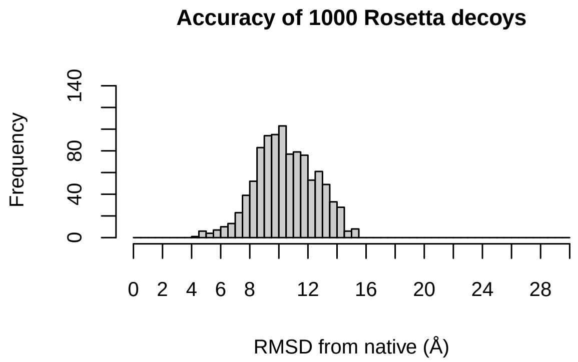


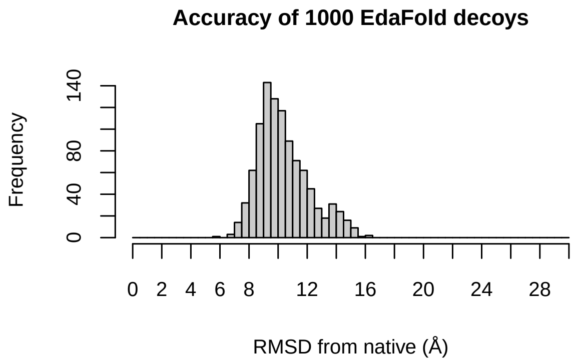

# 1elwA ( all-$\alpha$, 117 residues )

Sets of short Rosetta Runs | Long Rosetta Runs | EdaFoldAA

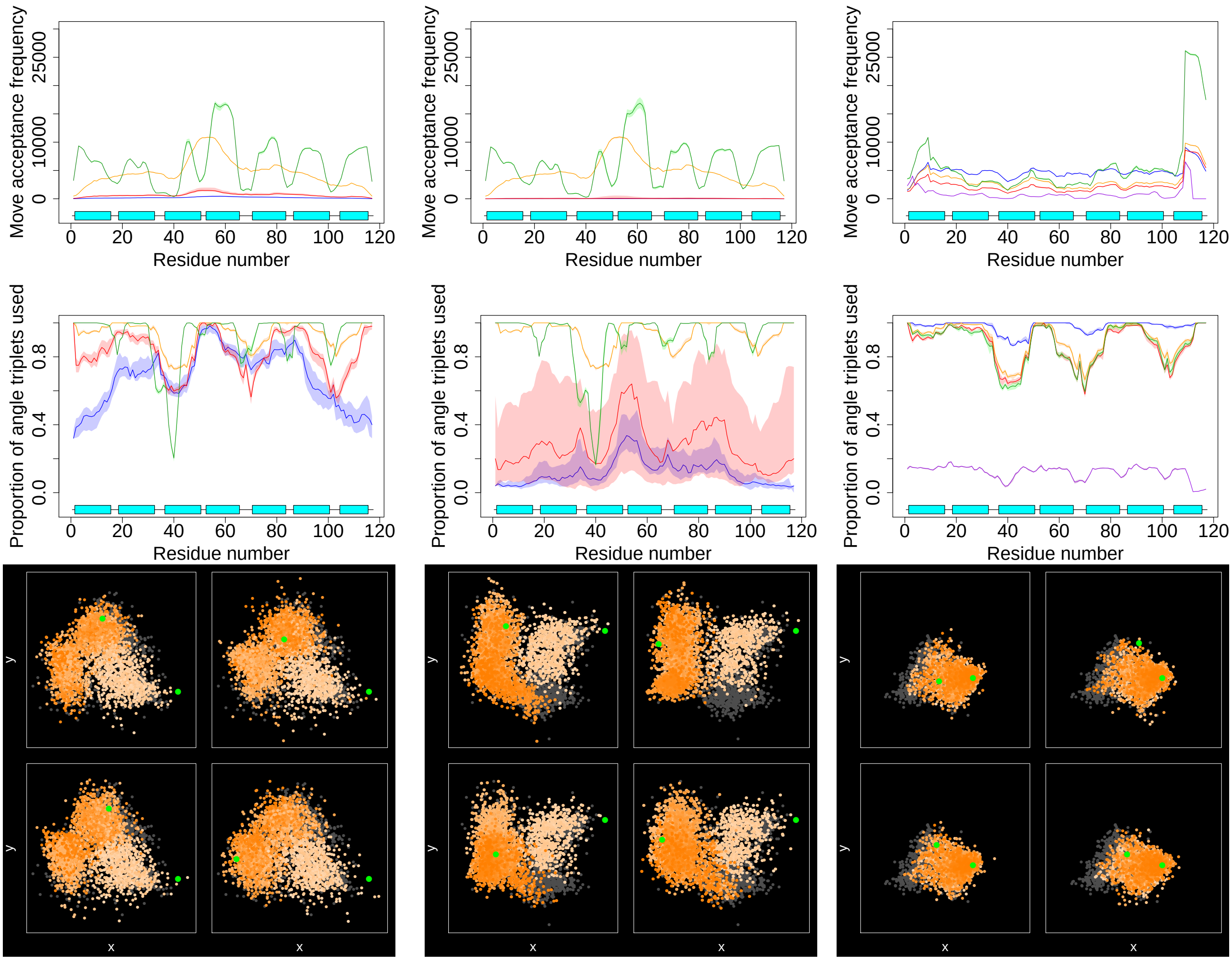


$f = 9.9212$ | $f = 10.51$ | $f = 10.795$

**Accuracy of 1000 Rosetta decoys**

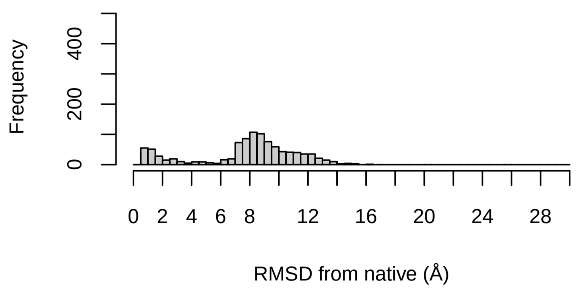


**Accuracy of 1000 EdaFold decoys**

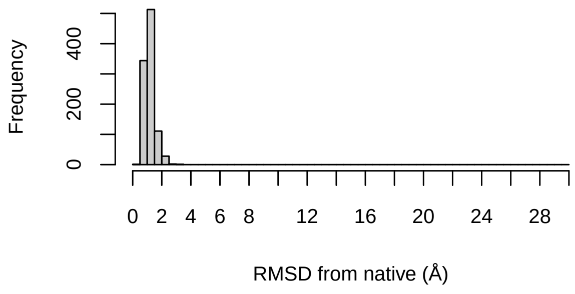

# 1bgf ( all-$\alpha$, 118 residues )

Sets of short Rosetta Runs | Long Rosetta Runs | EdaFoldAA

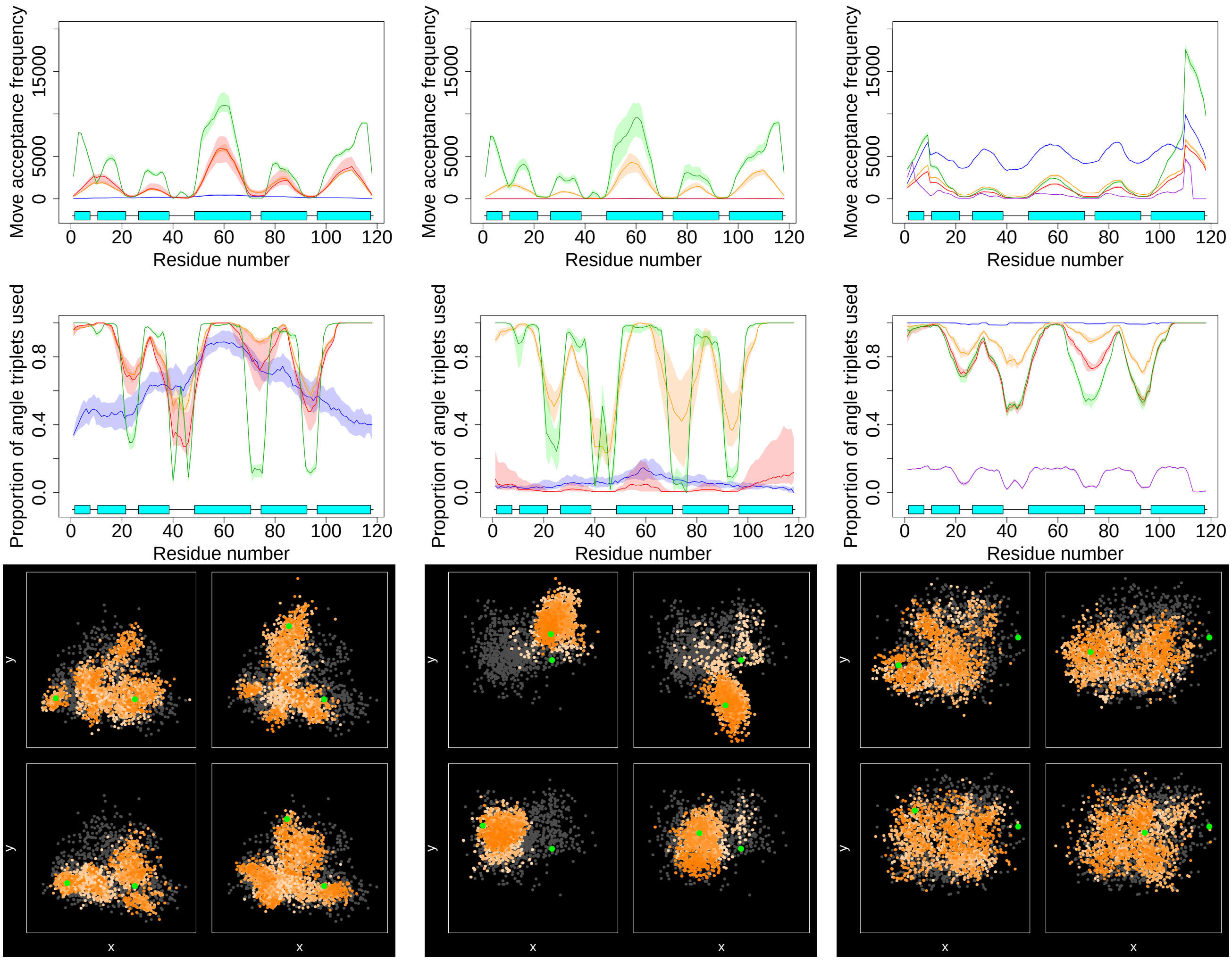


$f = 6.344$ | $f = 7.953$ | $f = 5.7092$

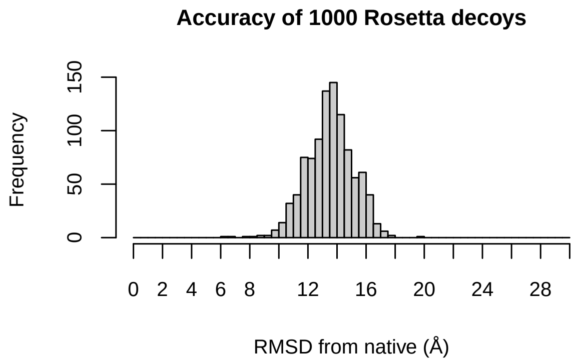


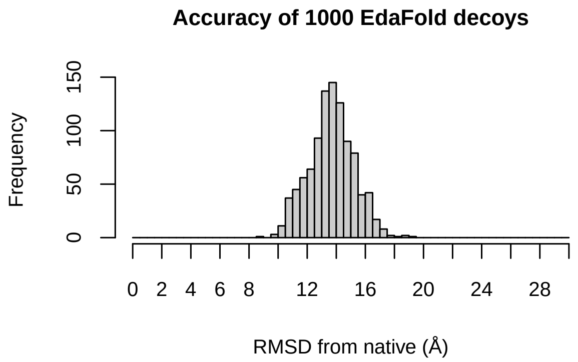

# 1lis ( all-$\alpha$, 125 residues )

Sets of short Rosetta Runs | Long Rosetta Runs | EdaFoldAA

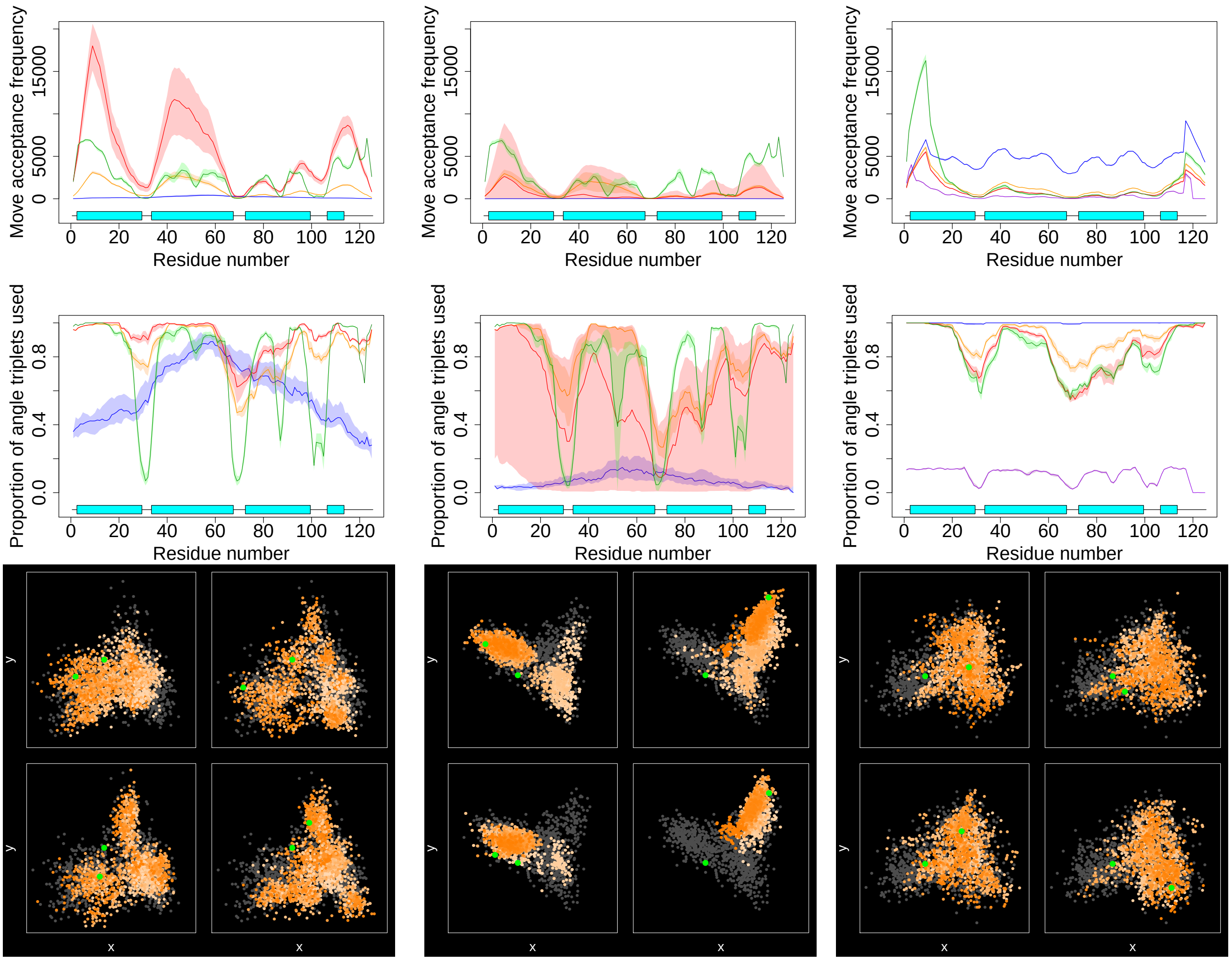


$f$ = 7.0561 | $f$ = 9.1876 | $f$ = 4.7426

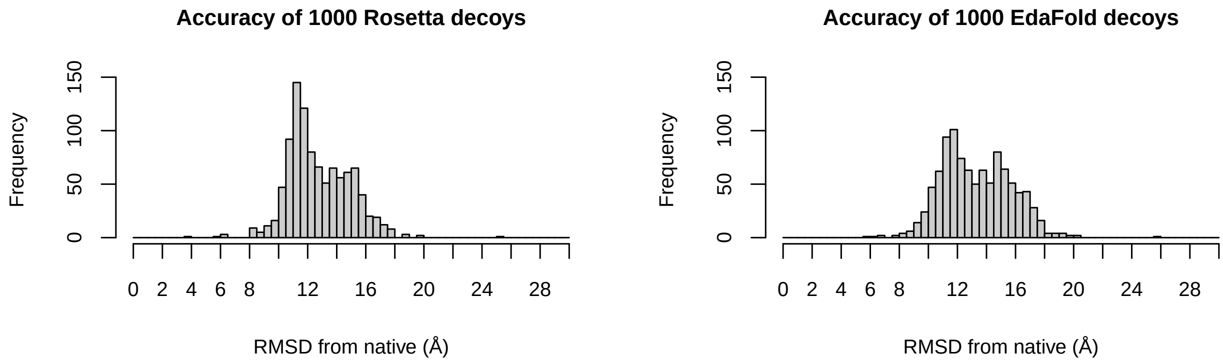

# 1eyvA ( all-$\alpha$, 131 residues )

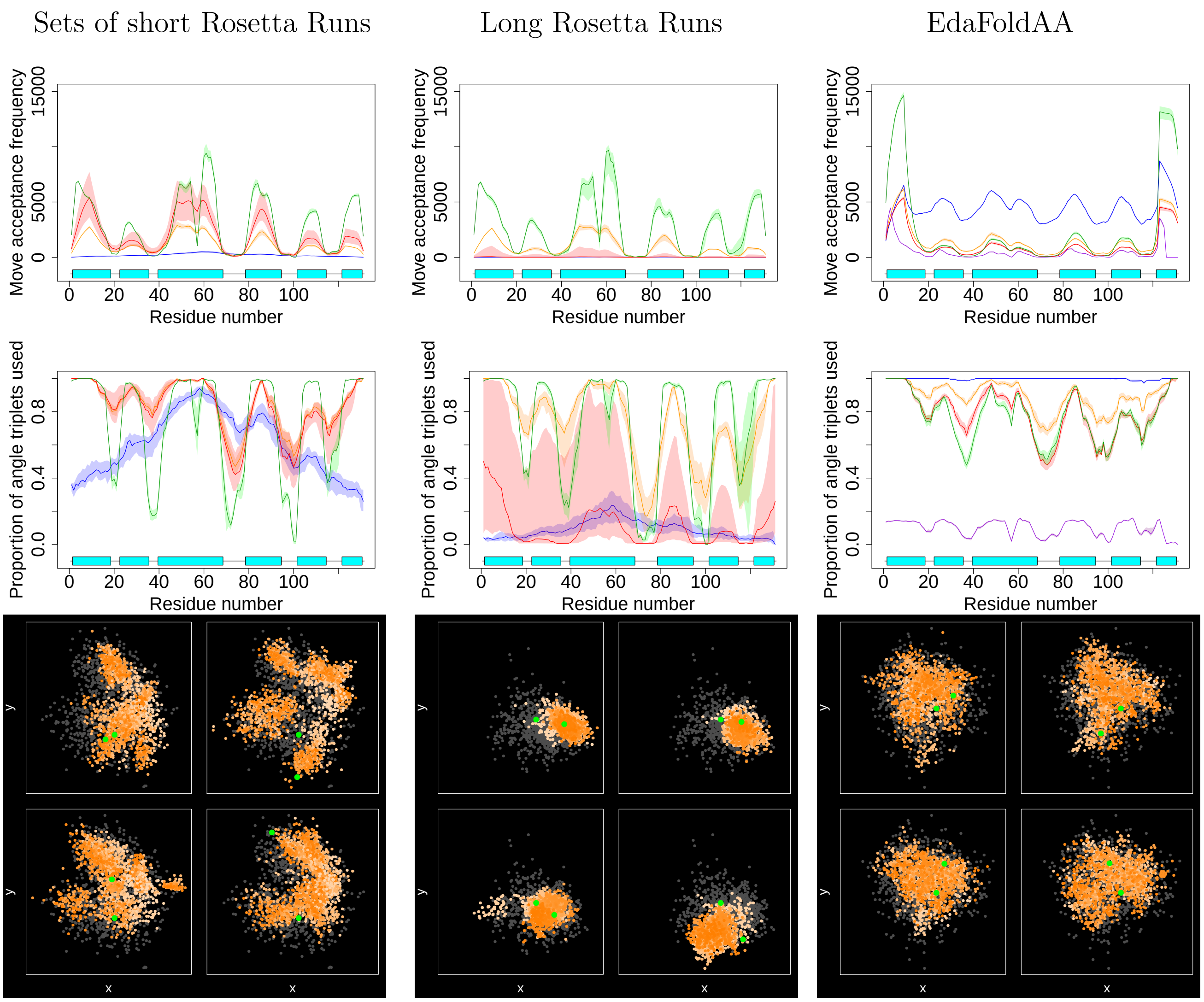


$f = 6.1202$ $f = 9.5257$ $f = 5.1275$

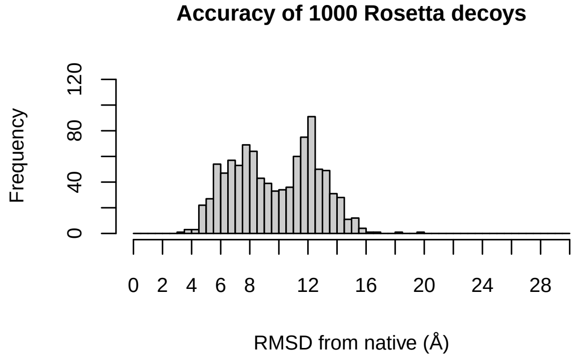


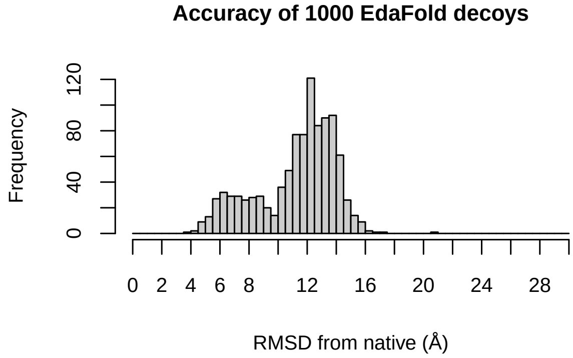

# 1cg5B ( all-$\alpha$, 141 residues )

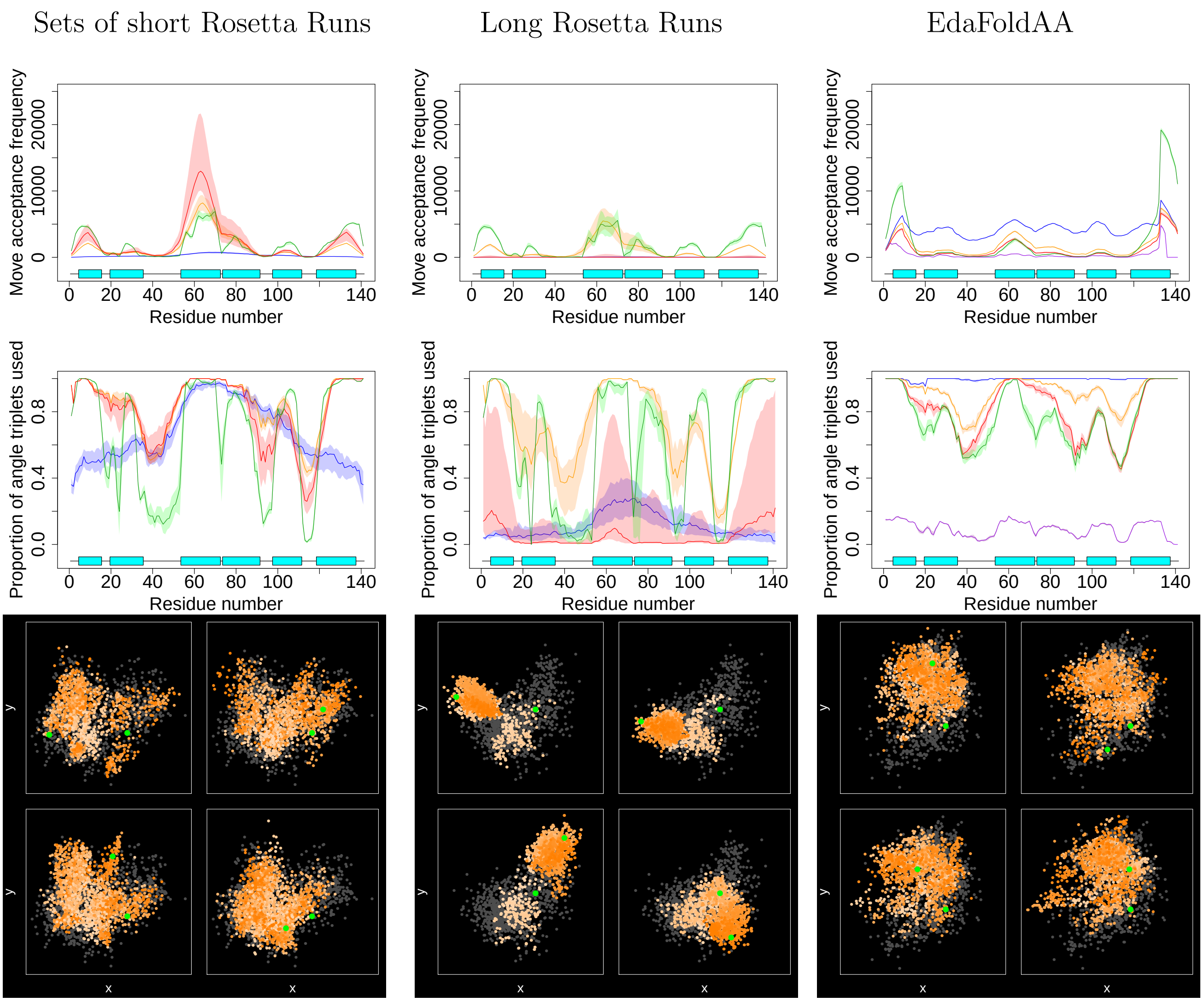


$f$ = 5.0799 $f$ = 8.0351 $f$ = 5.0366

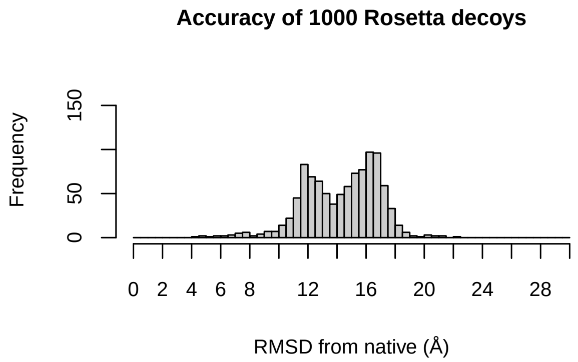


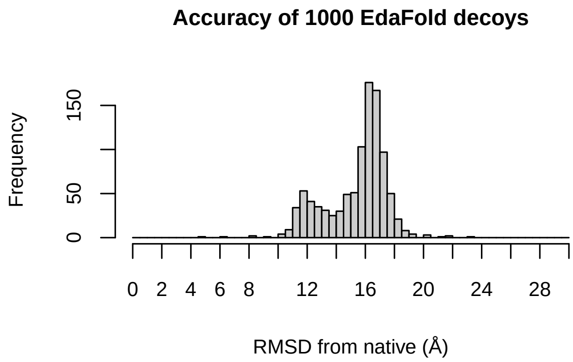

# 1a19A ( $\alpha/\beta$, 89 residues )

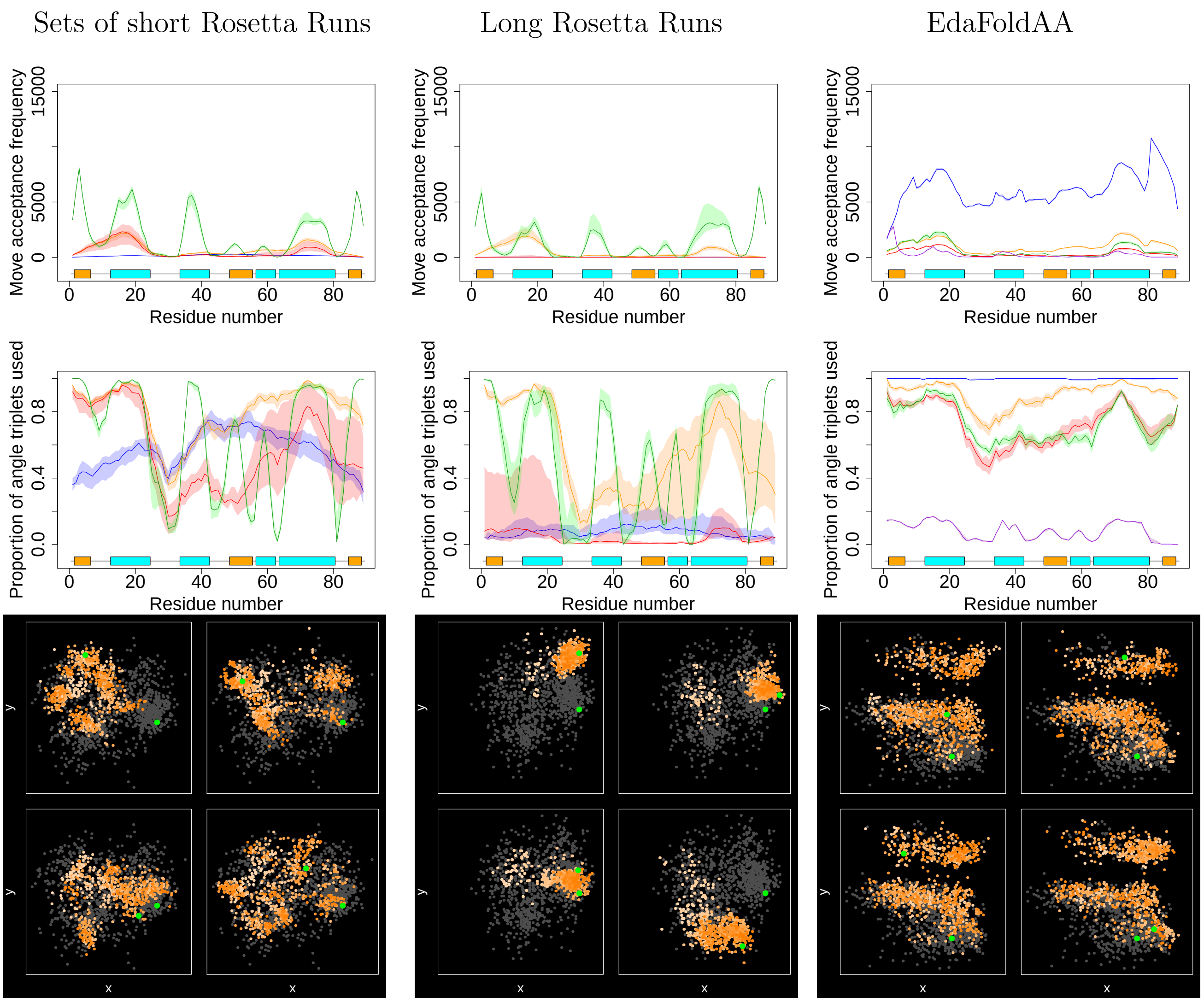


$f = 11.033$ $f = 17.343$ $f = 12.46$

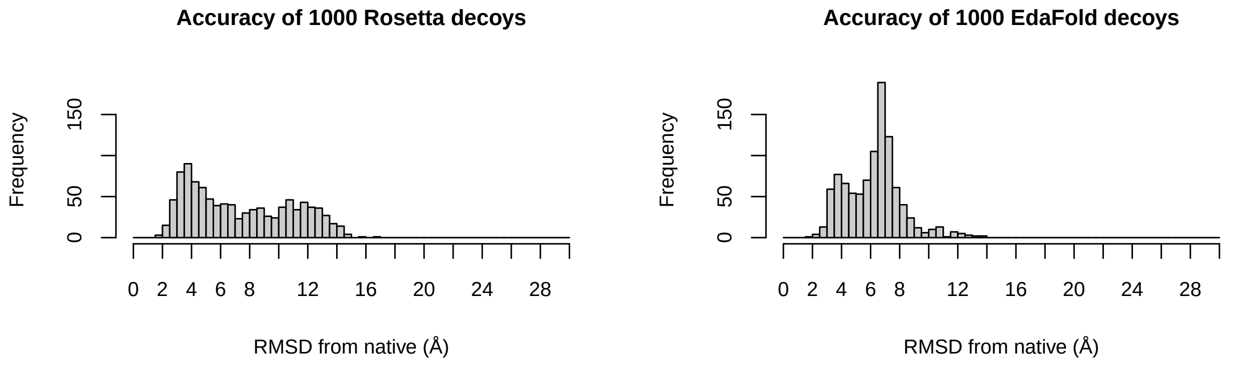

# 1iibA ( $\alpha/\beta$, 103 residues )

Sets of short Rosetta Runs | Long Rosetta Runs | EdaFoldAA

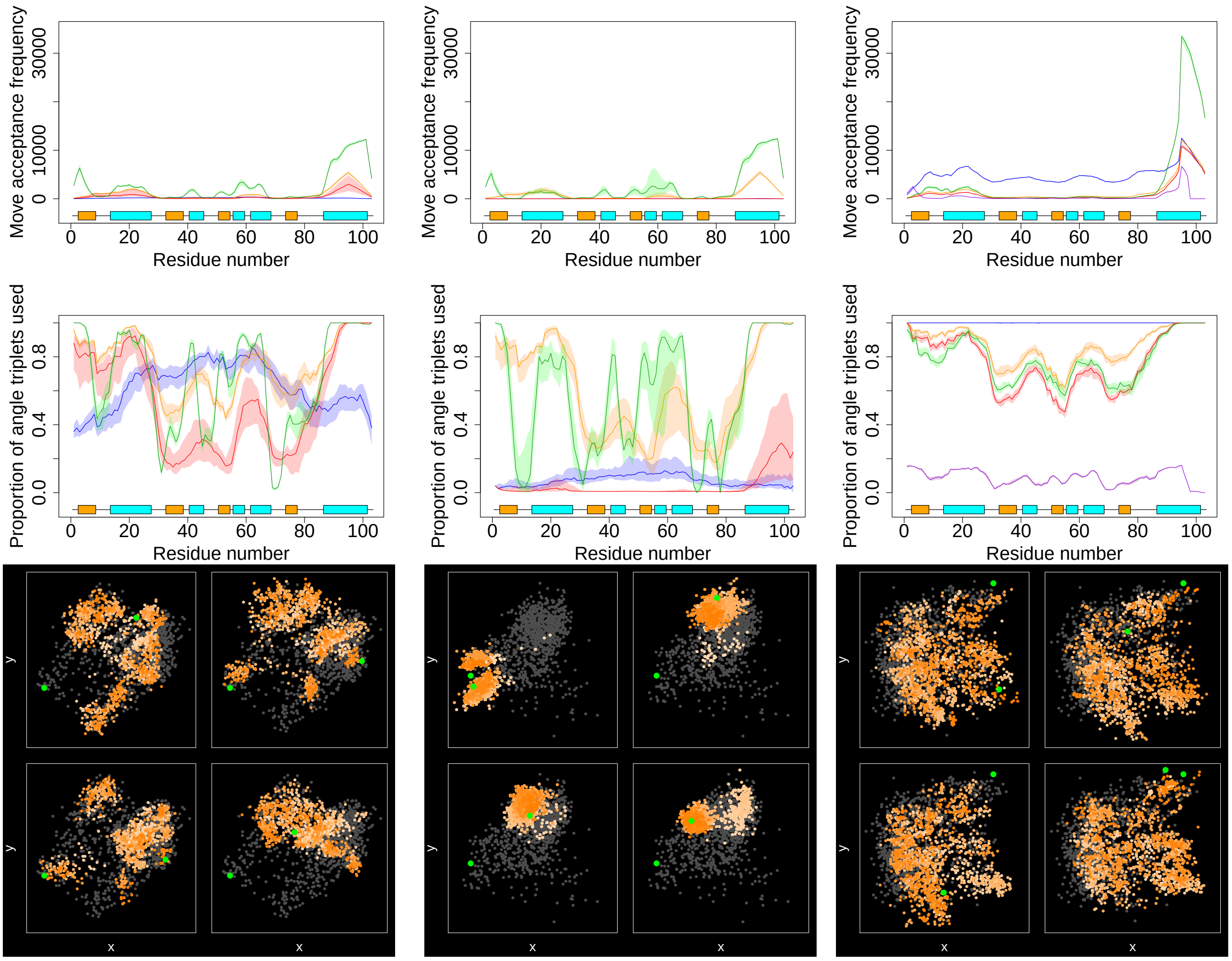


$f = 8.2218$ | $f = 17.955$ | $f = 10.905$

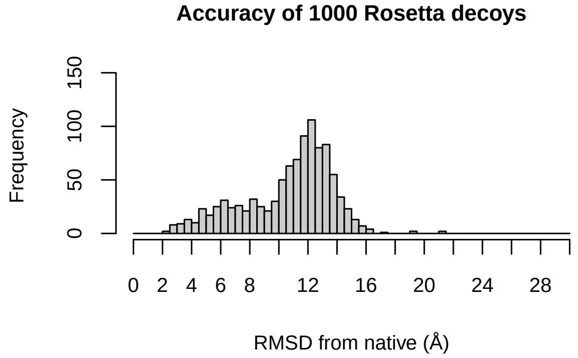


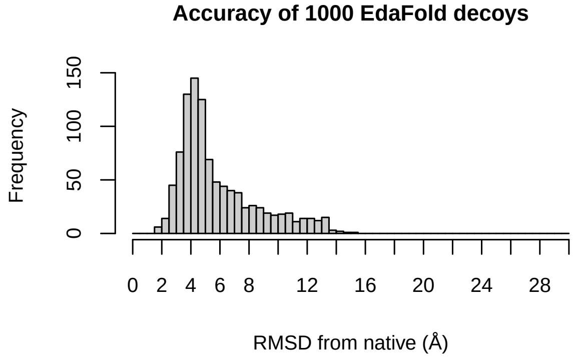

# 1aiu ( $\alpha/\beta$, 105 residues )

Sets of short Rosetta Runs | Long Rosetta Runs | EdaFoldAA

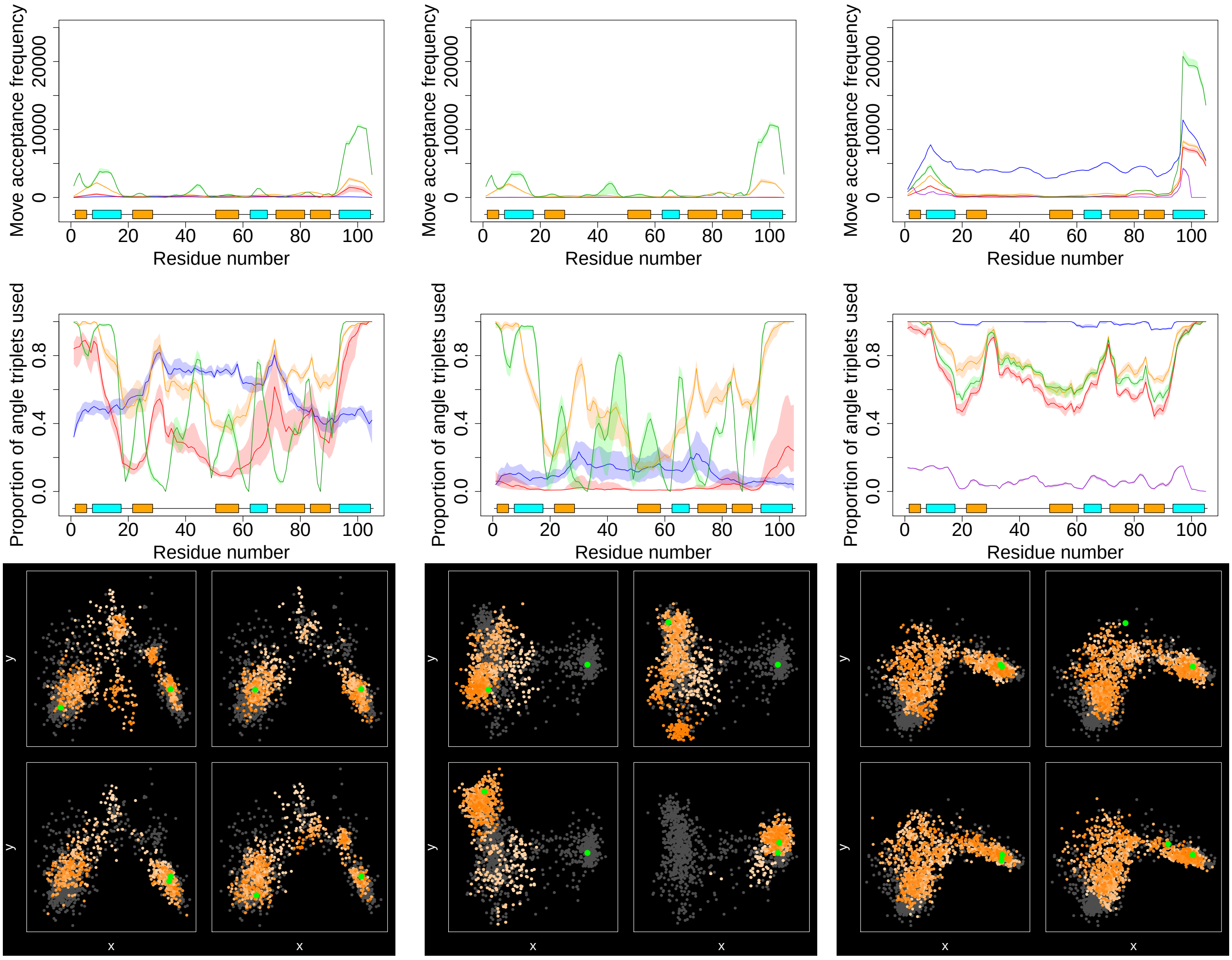


$f$ = 20.19 | $f$ = 25.059 | $f$ = 19.227

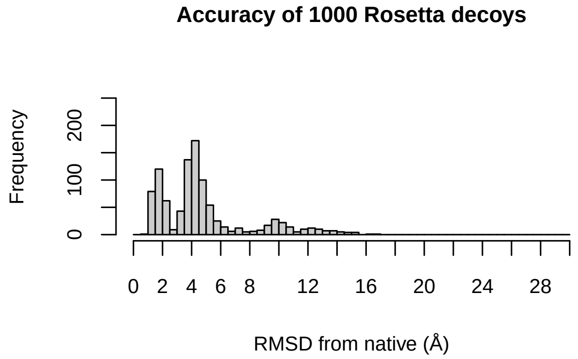


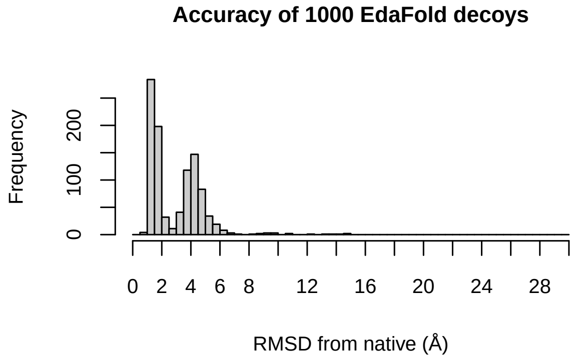

# 2chf ( $\alpha/\beta$, 128 residues )

Sets of short Rosetta Runs | Long Rosetta Runs | EdaFoldAA

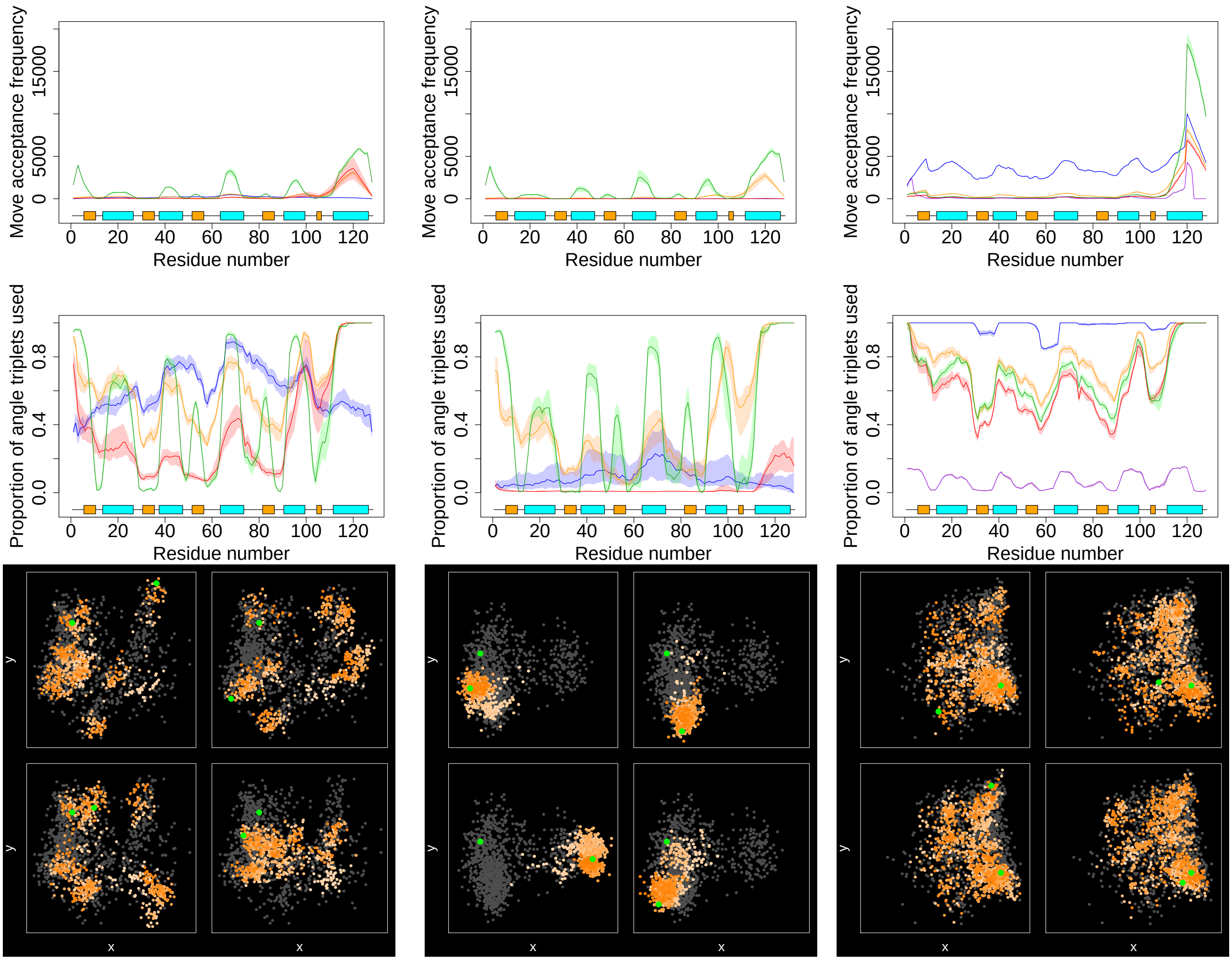


$f = 10.559$ | $f = 18.894$ | $f = 12.347$

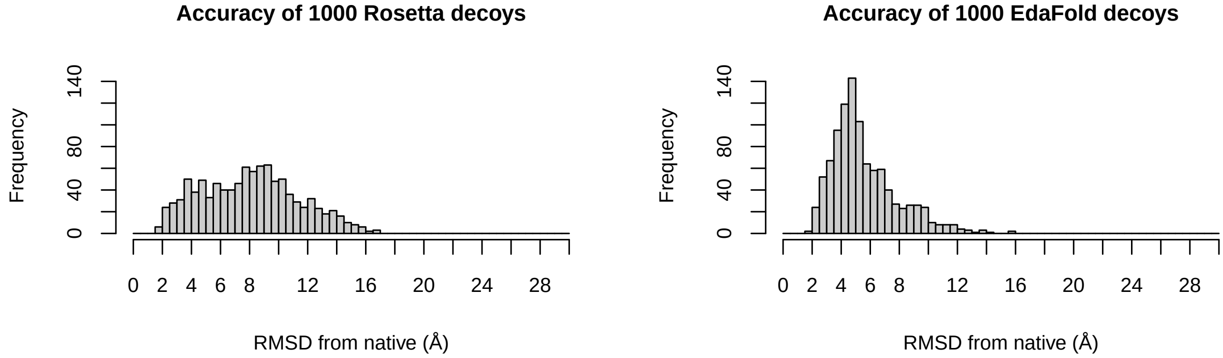

# 1ptq ( $\alpha + \beta$, 50 residues )

Sets of short Rosetta Runs | Long Rosetta Runs | EdaFoldAA

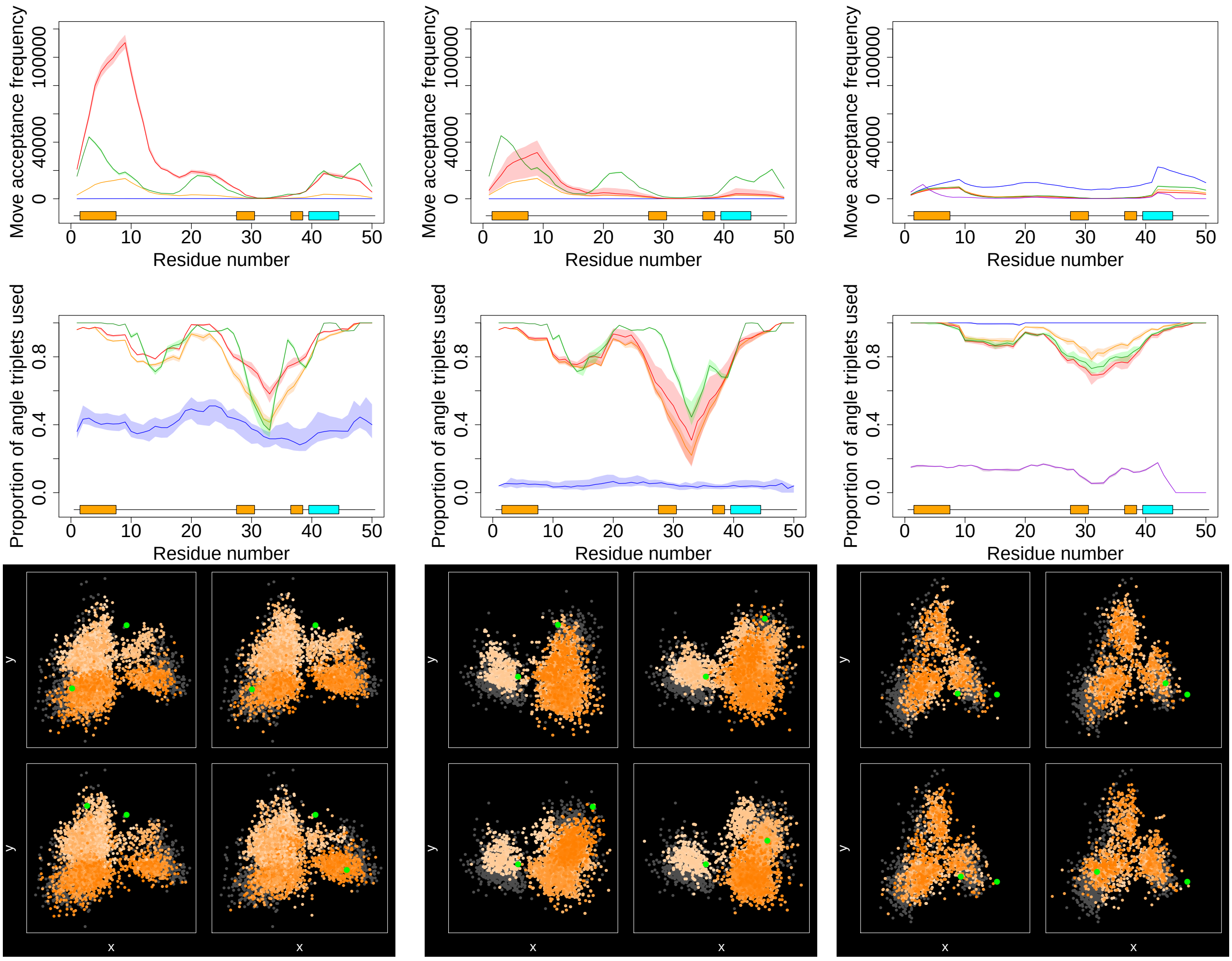


$f = 10.587$ | $f = 11.603$ | $f = 8.6355$

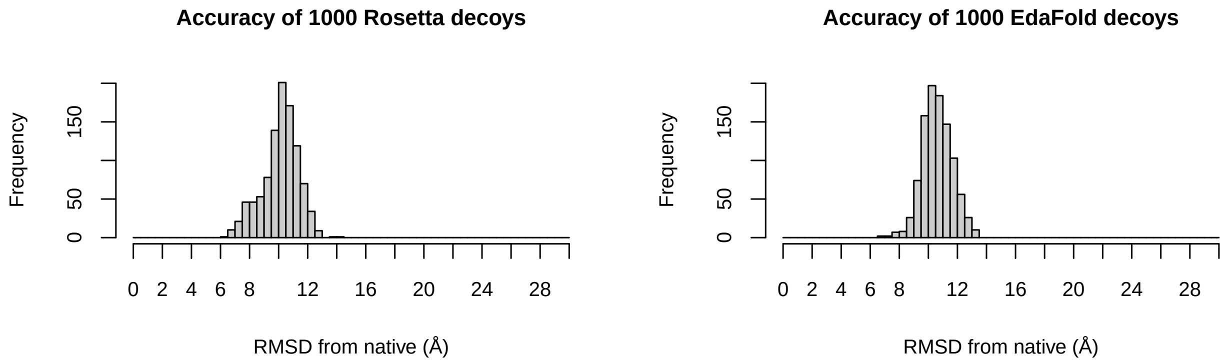

# 1pgx ( $\alpha + \beta$, 55 residues )

Sets of short Rosetta Runs | Long Rosetta Runs | EdaFoldAA

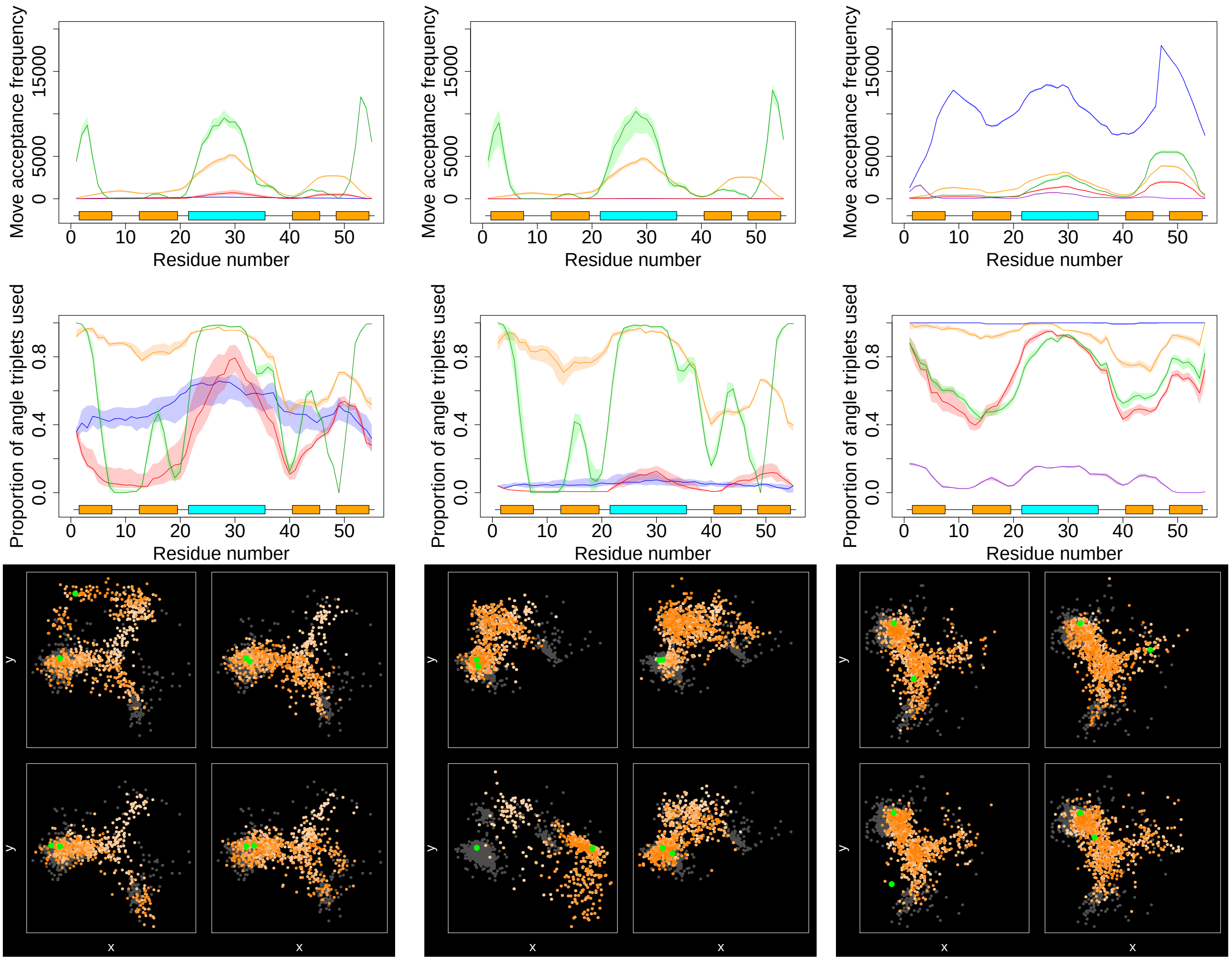


$f$ = 16.788 | $f$ = 19.83 | $f$ = 21.503

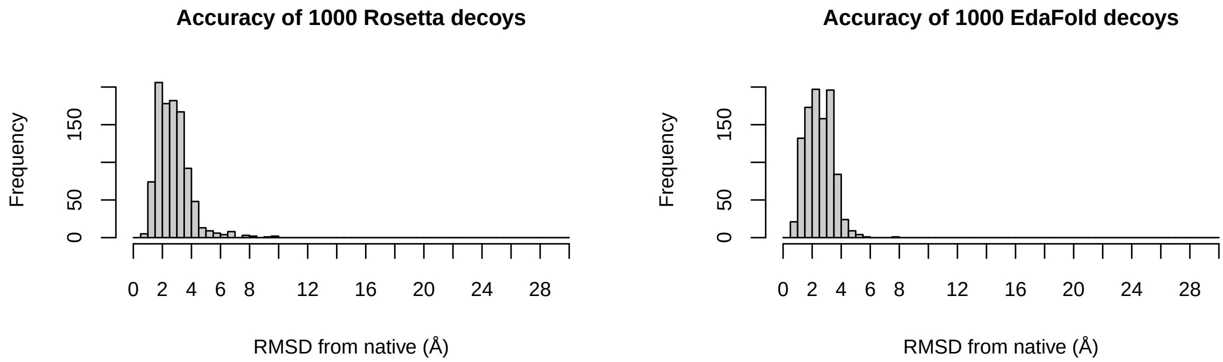

## 1b3aA ( $\alpha + \beta$, 55 residues )

Sets of short Rosetta Runs | Long Rosetta Runs | EdaFoldAA

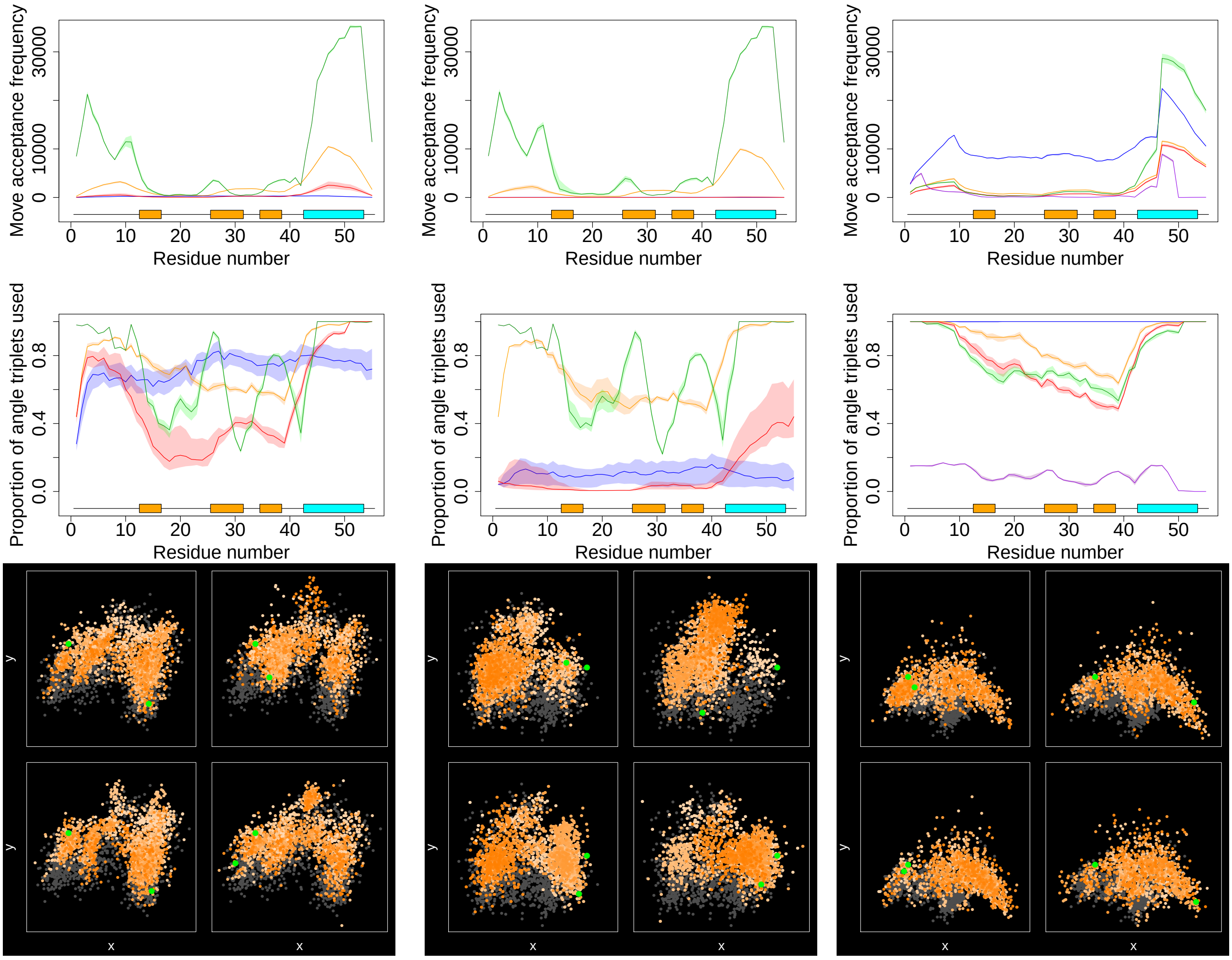


$f$ = 11.482 | $f$ = 11.373 | $f$ = 12.134

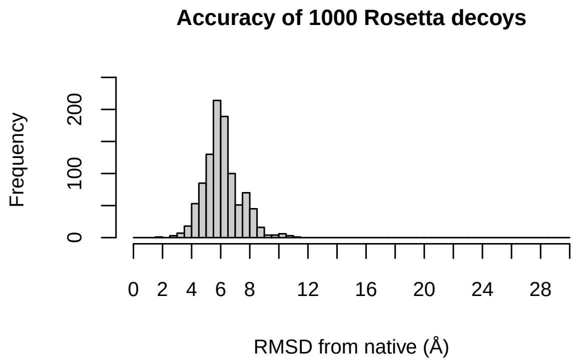


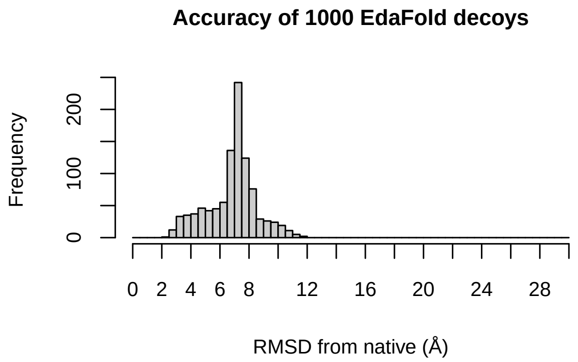

# 5croA ( $\alpha + \beta$, 55 residues )

Sets of short Rosetta Runs | Long Rosetta Runs | EdaFoldAA

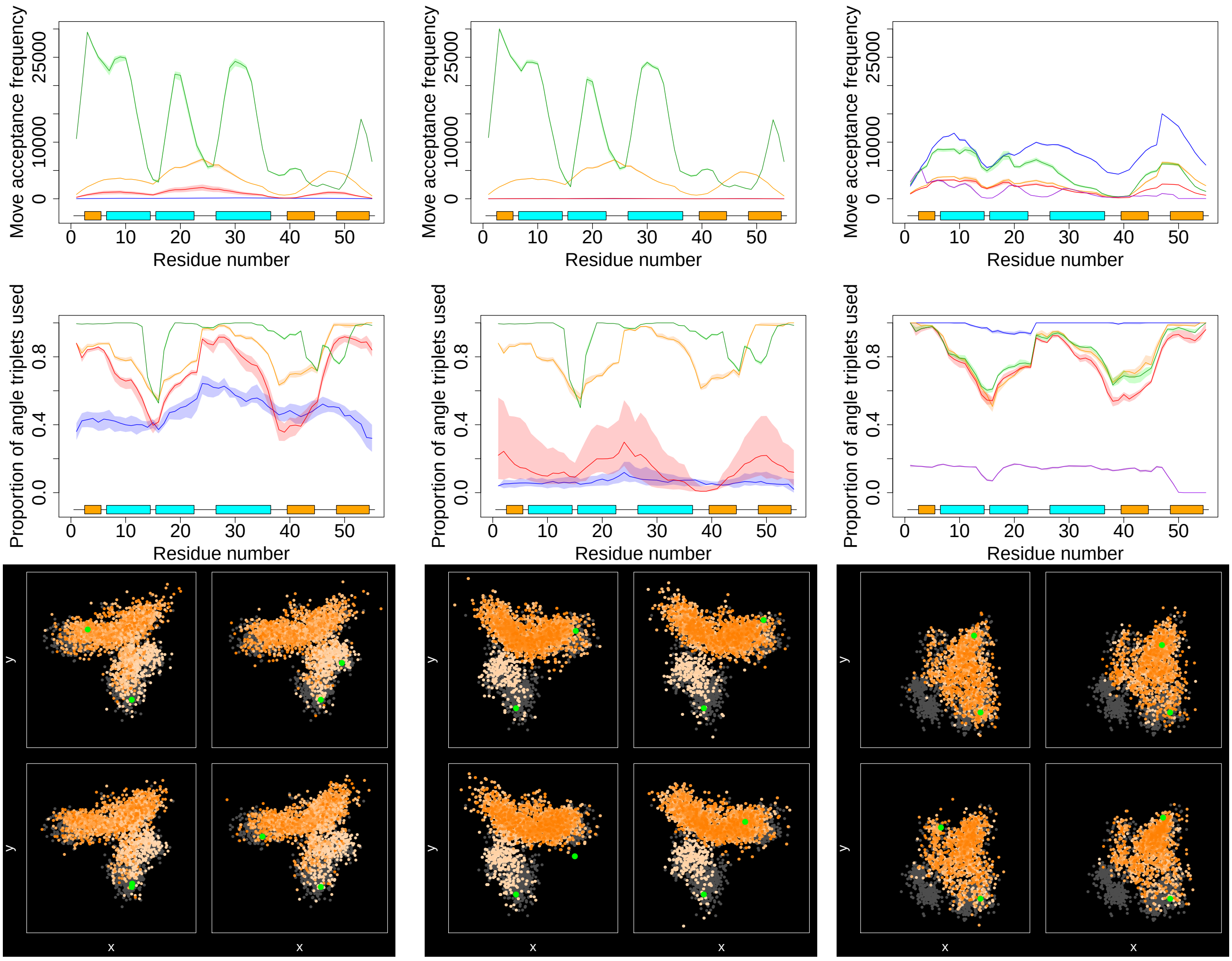


$f$ = 14.315 | $f$ = 14.678 | $f$ = 14.174

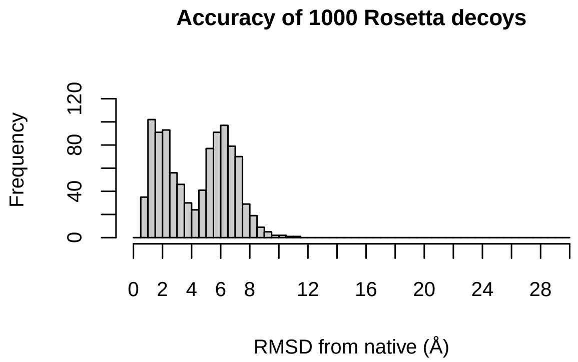


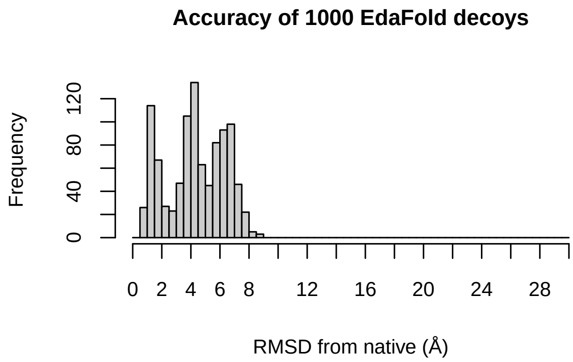

# 1tif ( $\alpha + \beta$, 59 residues )

Sets of short Rosetta Runs | Long Rosetta Runs | EdaFoldAA

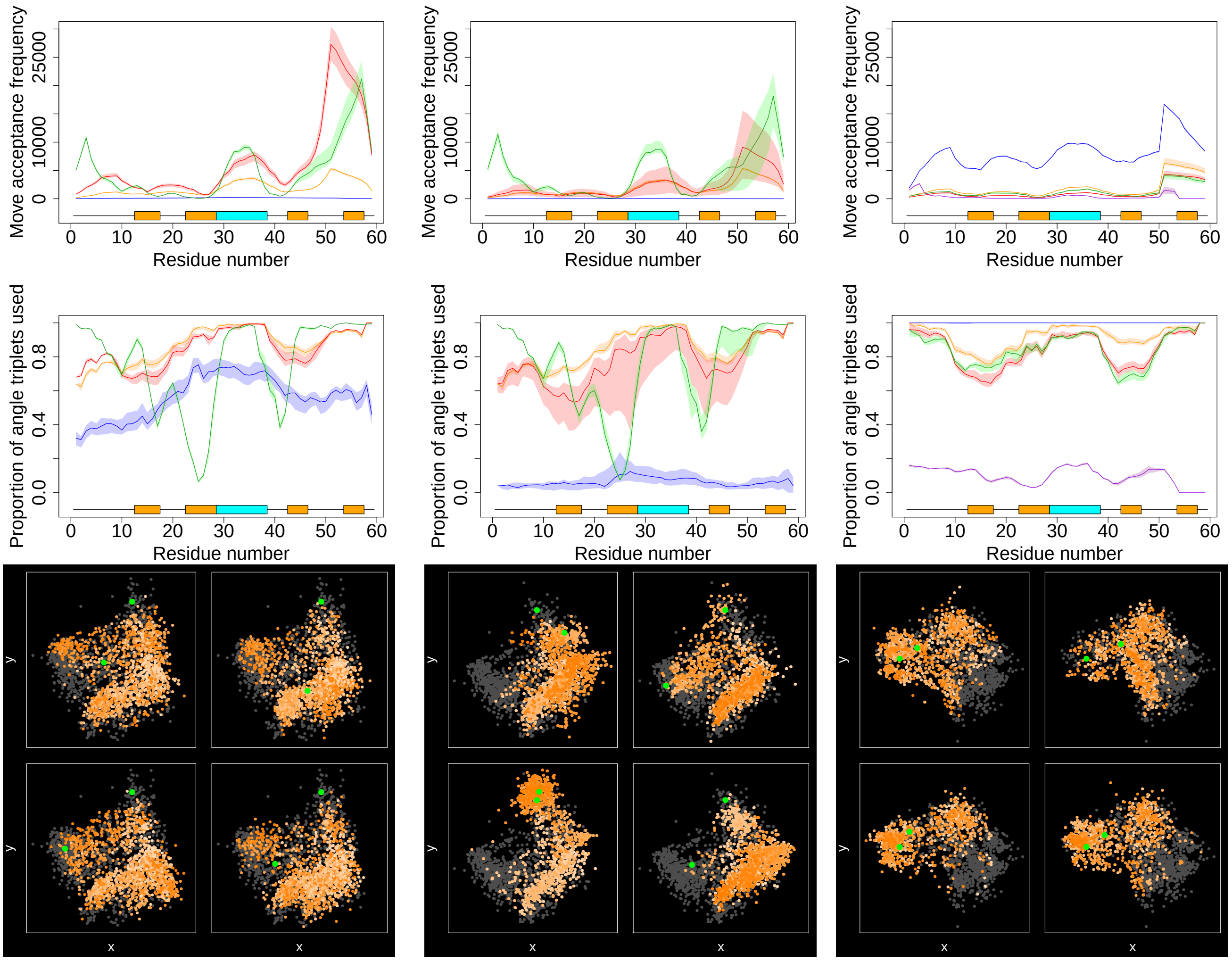


$f = 12.368$ | $f = 13.69$ | $f = 15.739$

**Accuracy of 1000 Rosetta decoys**

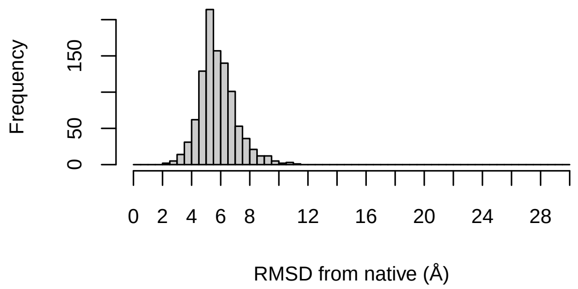


**Accuracy of 1000 EdaFold decoys**

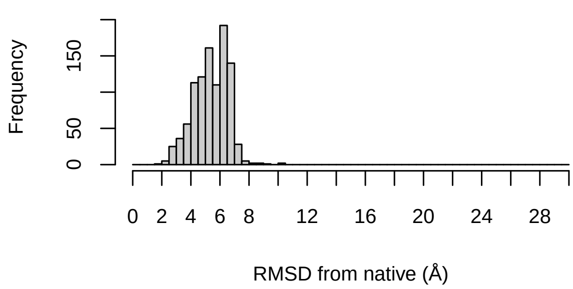

# 1hz6A ( $\alpha + \beta$, 61 residues )

Sets of short Rosetta Runs | Long Rosetta Runs | EdaFoldAA

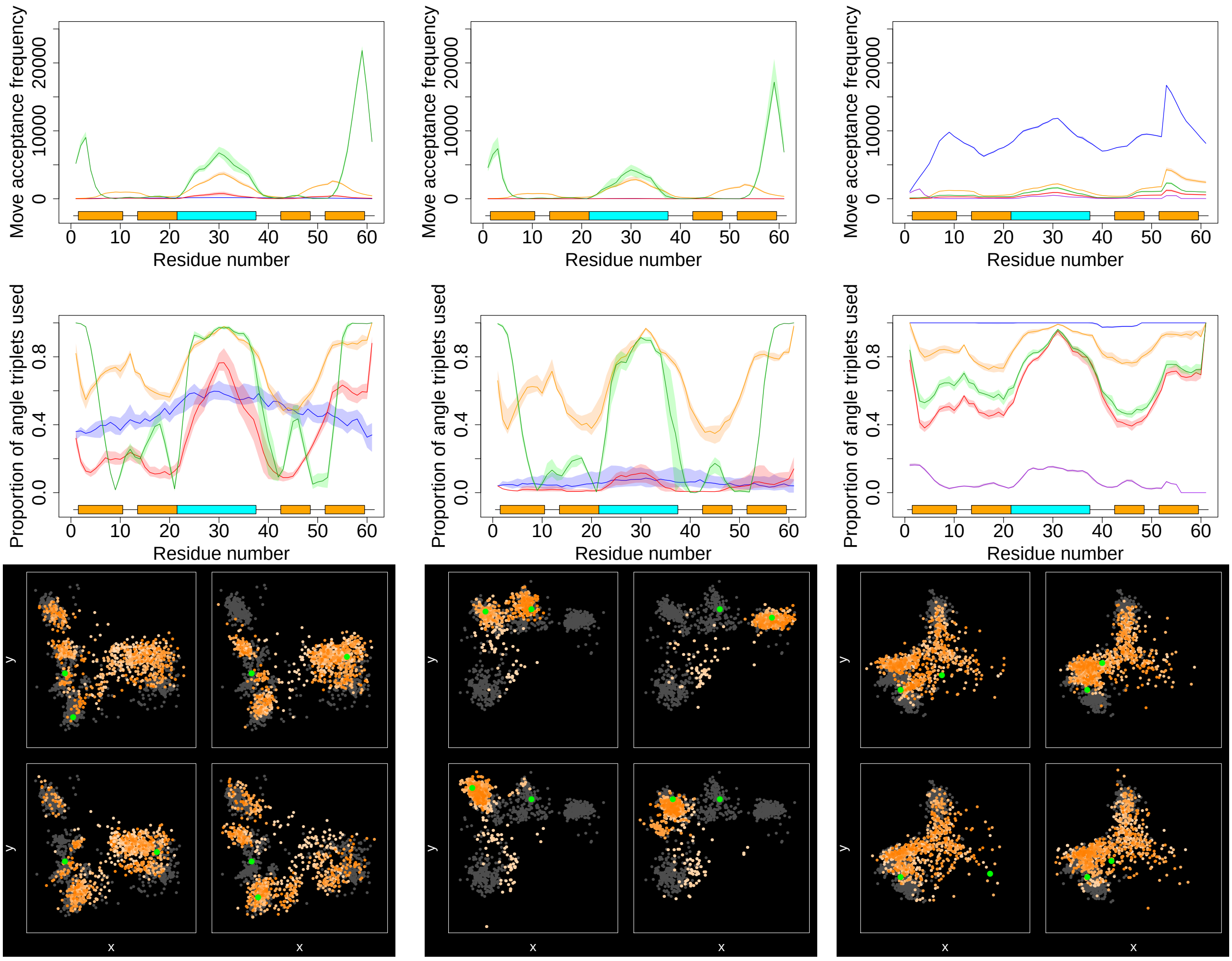


$f = 24.668$ | $f = 29.52$ | $f = 22.062$

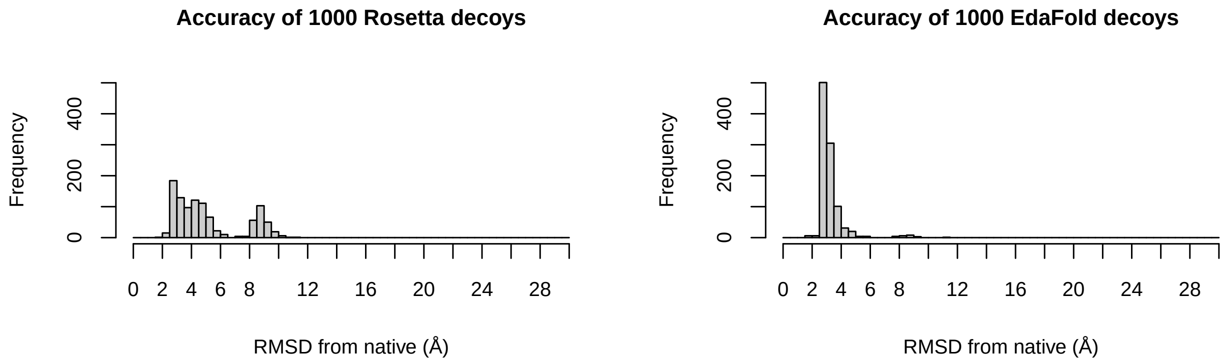

# 2ci2I ( $\alpha + \beta$, 62 residues )

Sets of short Rosetta Runs | Long Rosetta Runs | EdaFoldAA

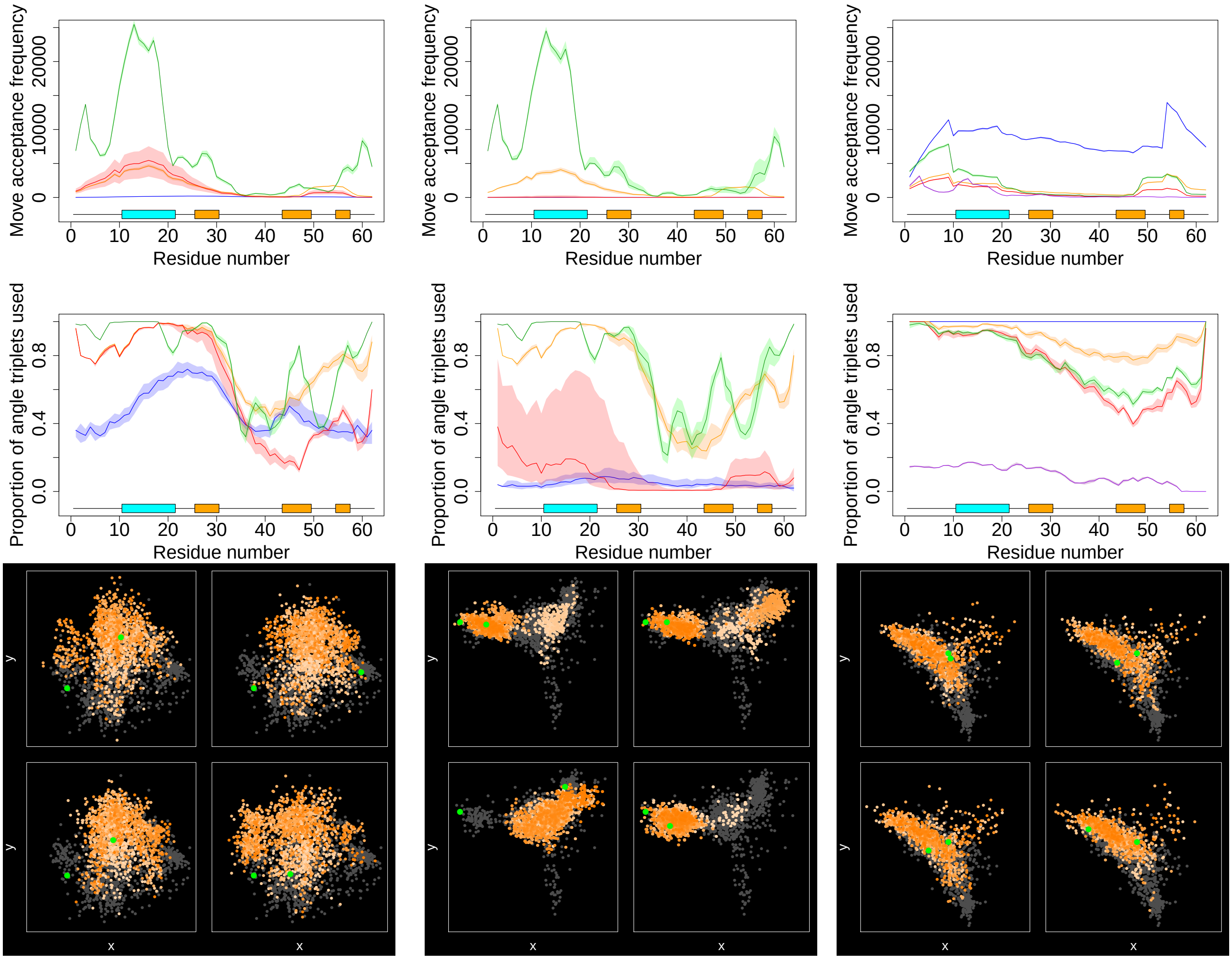


$f$ = 8.5886 | $f$ = 14.622 | $f$ = 13.351

**Accuracy of 1000 Rosetta decoys**

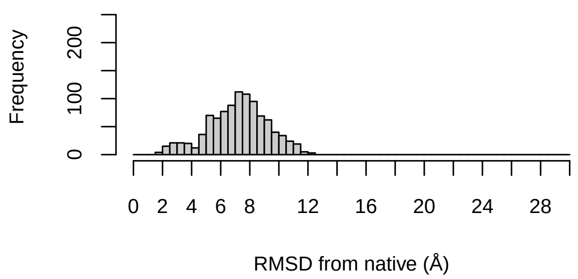


**Accuracy of 1000 EdaFold decoys**

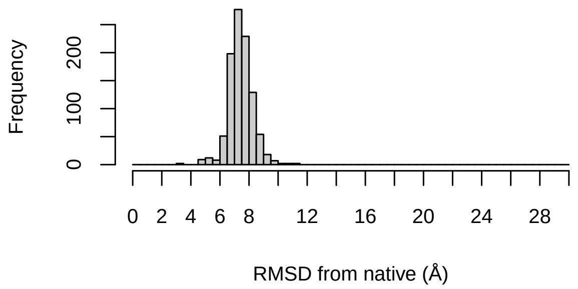

# 1scjB ( $\alpha + \beta$, 66 residues )

Sets of short Rosetta Runs | Long Rosetta Runs | EdaFoldAA

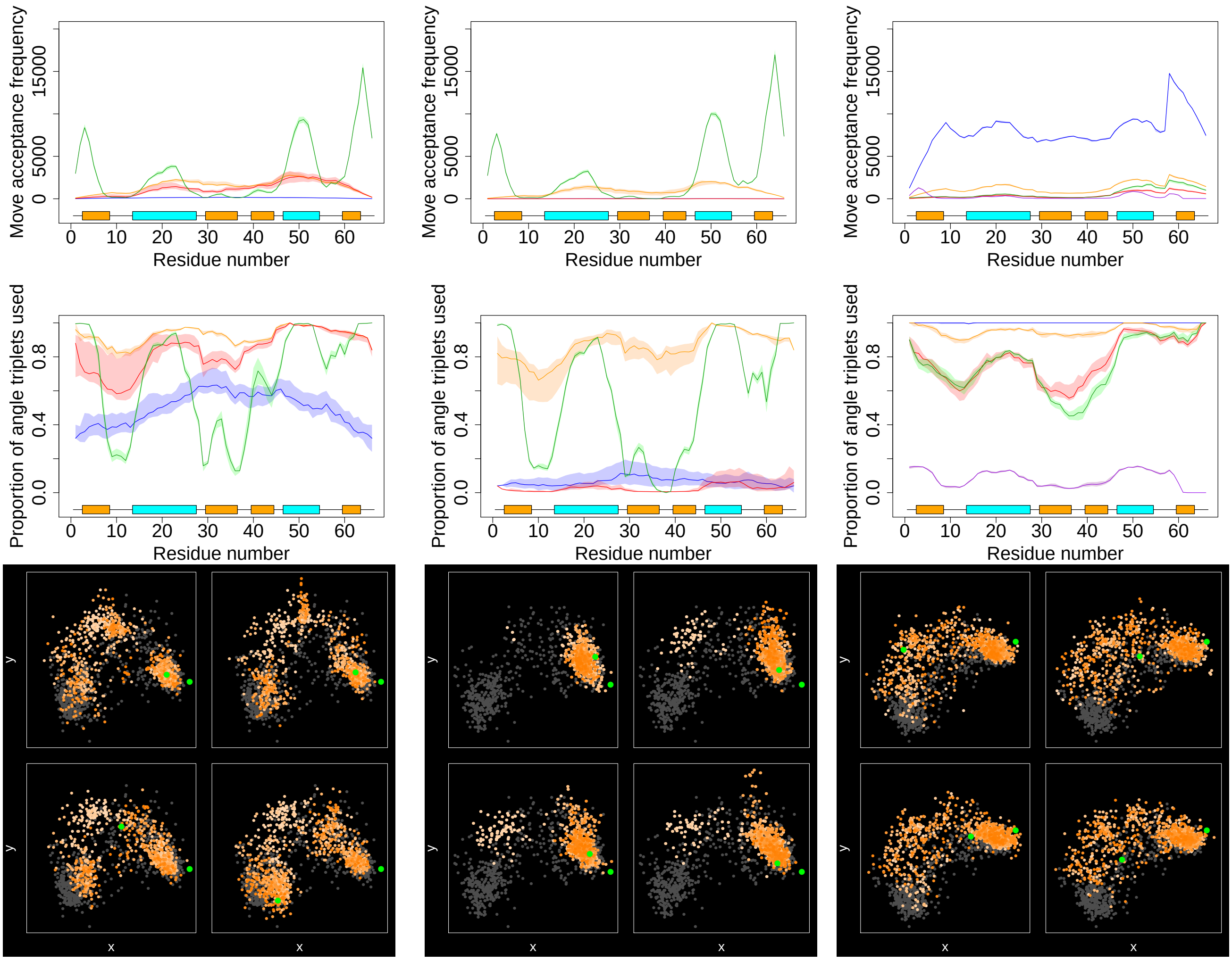


$f = 24.794$ | $f = 18.801$ | $f = 26.539$

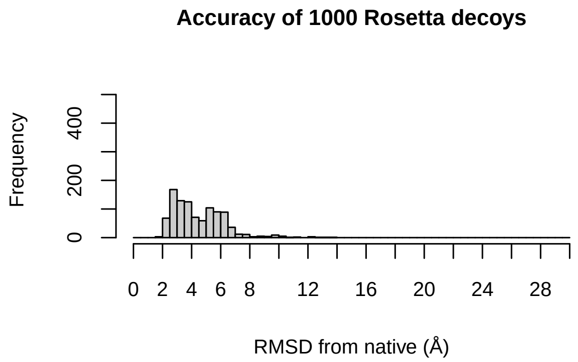


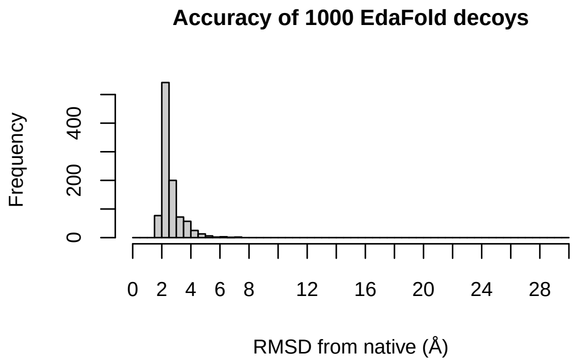

# 1ctf ( $\alpha + \beta$, 68 residues )

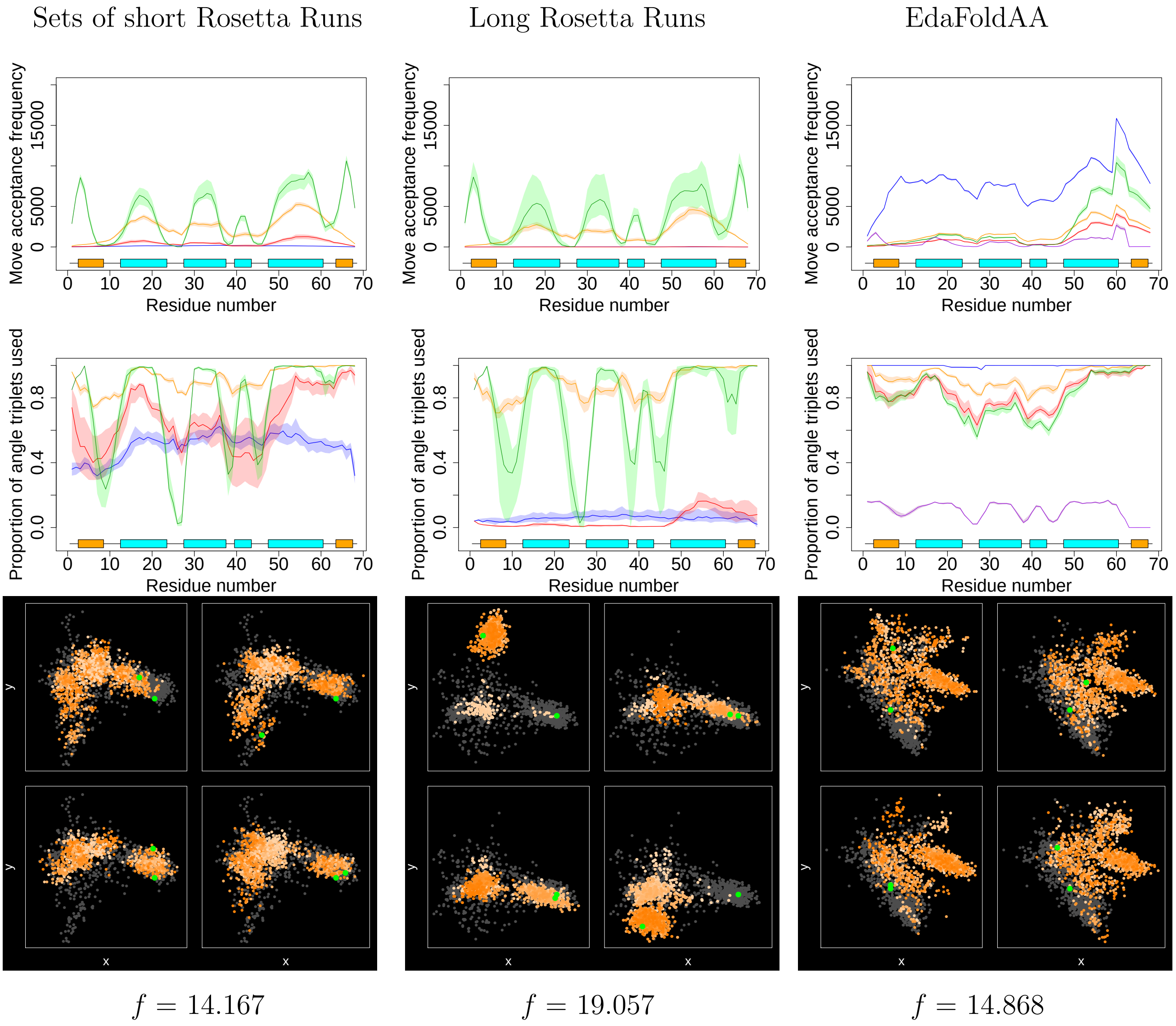


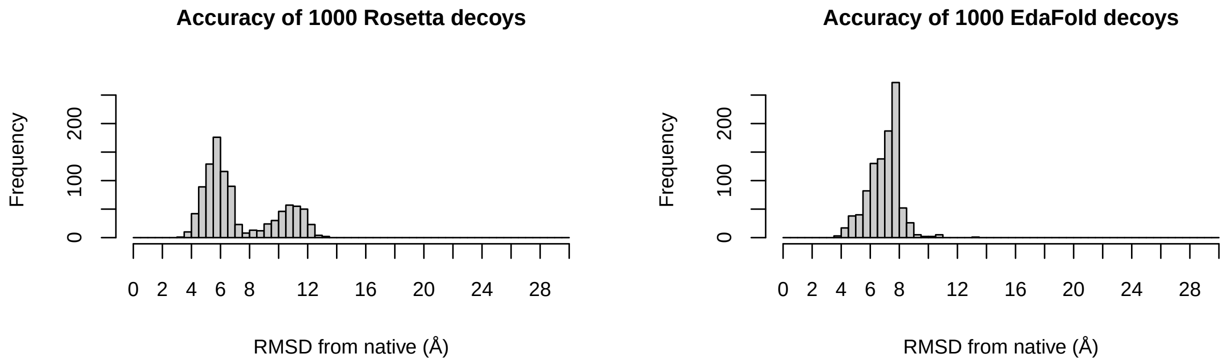

# 1ubi ( $\alpha + \beta$, 71 residues )

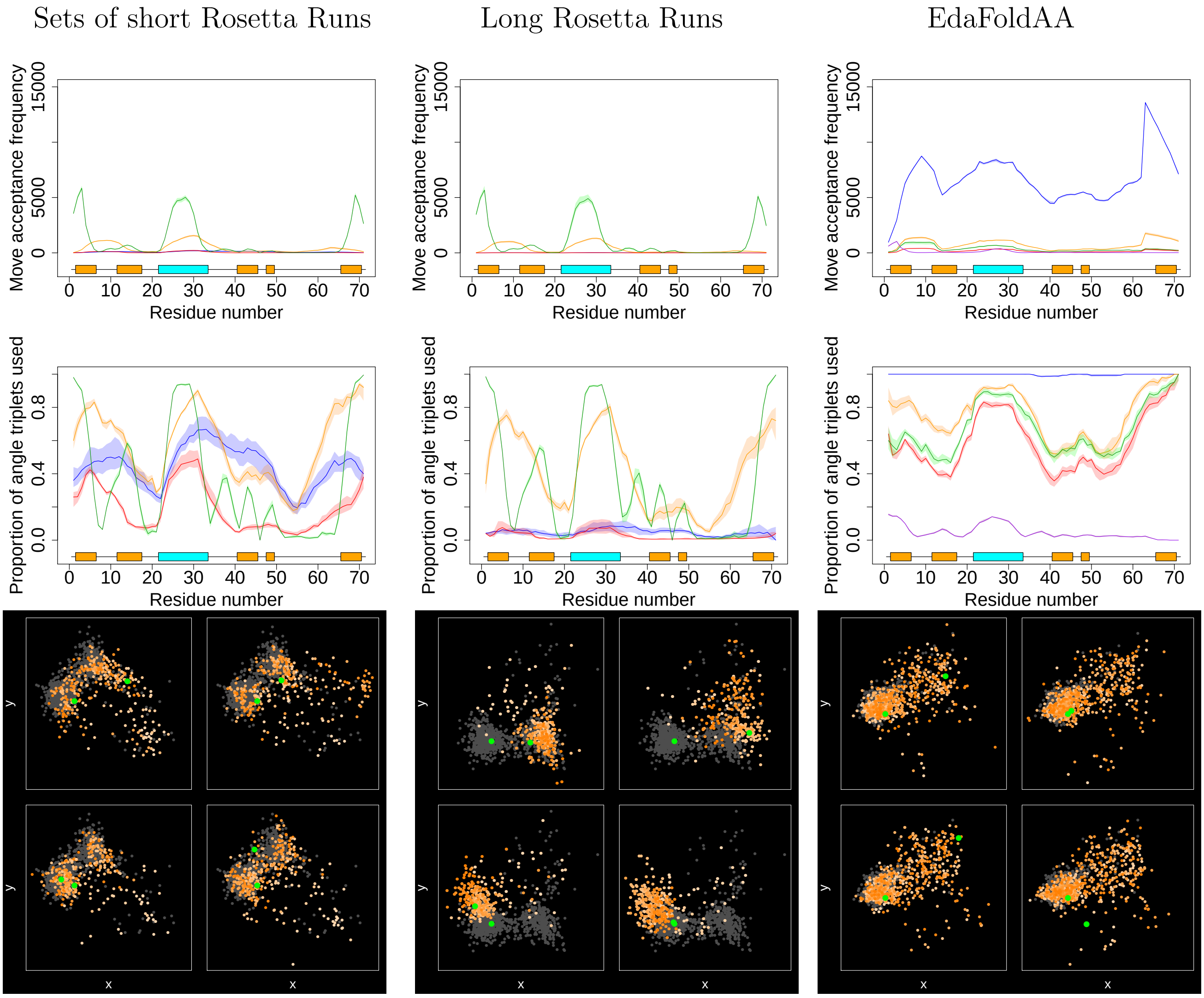


$f = 13.264$ $f = 12.293$ $f = 18.87$

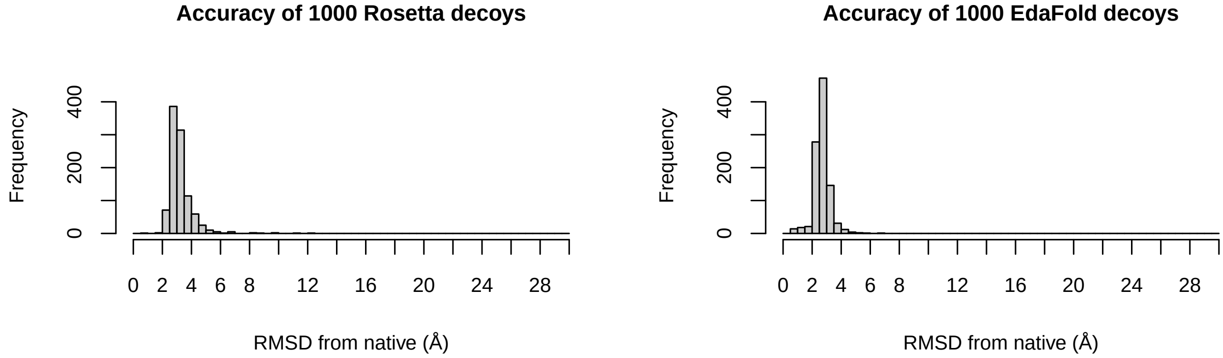

# 1cc8A ( $\alpha + \beta$, 72 residues )

Sets of short Rosetta Runs | Long Rosetta Runs | EdaFoldAA

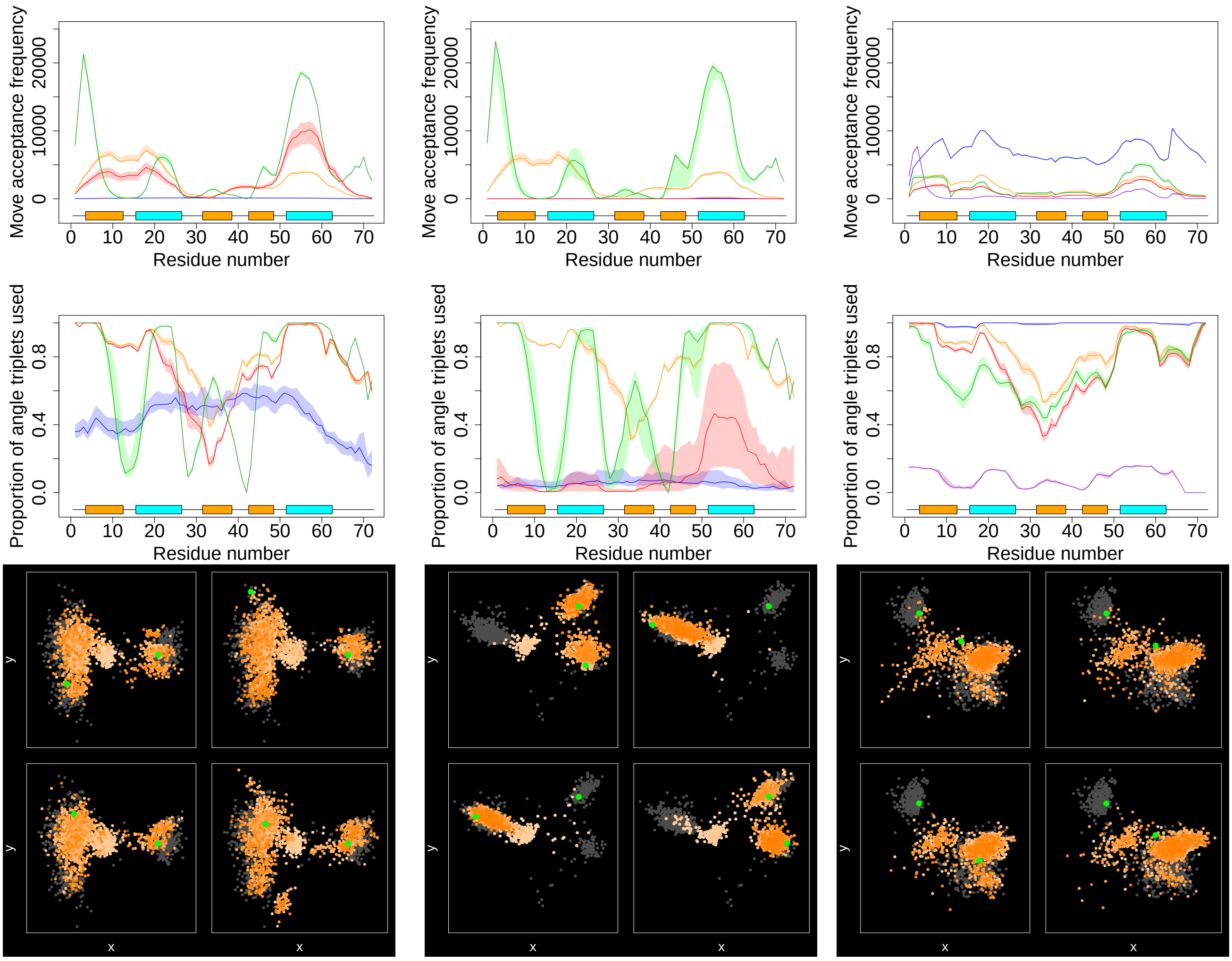


$f$ = 15.813 | $f$ = 22.96 | $f$ = 17.2

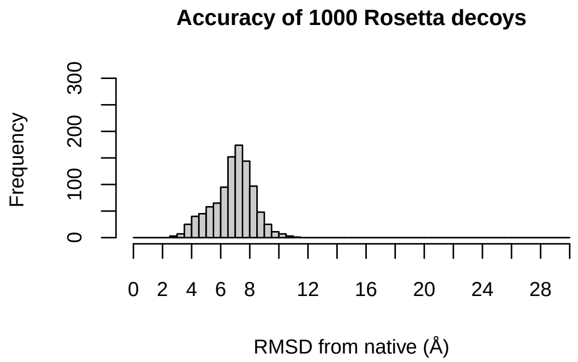


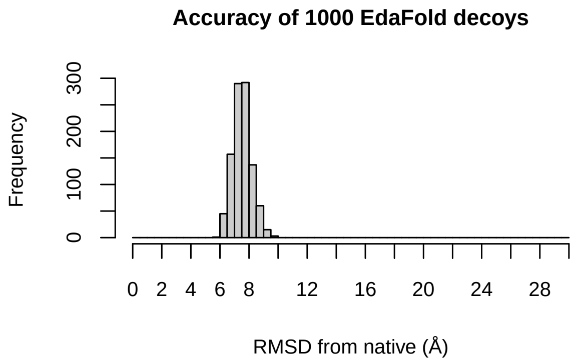

## 1vcc ( $\alpha + \beta$, 77 residues )

Sets of short Rosetta Runs | Long Rosetta Runs | EdaFoldAA

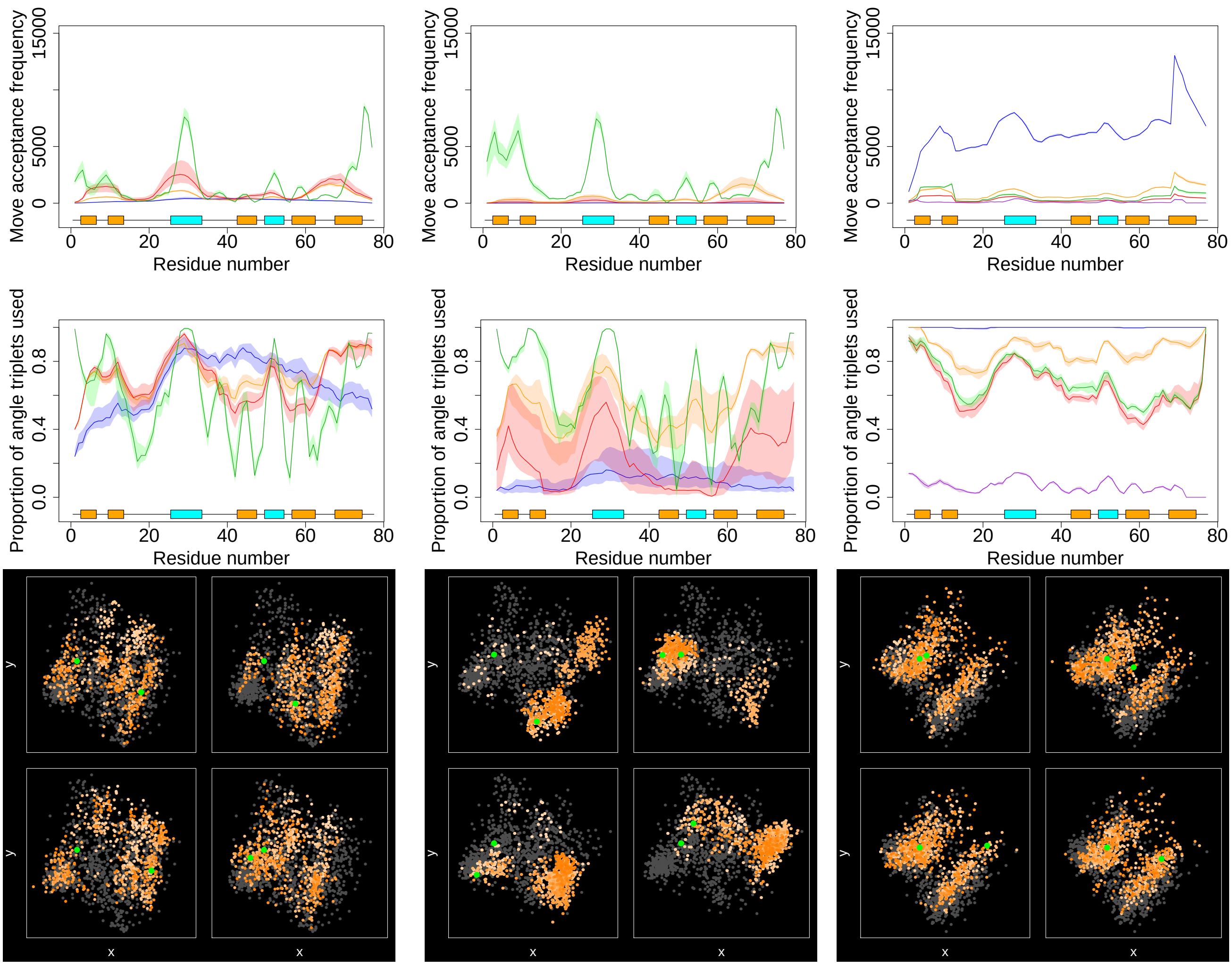


$f = 10.789$ | $f = 13.257$ | $f = 13.138$

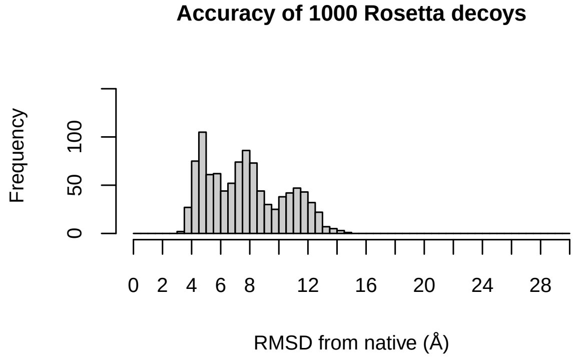


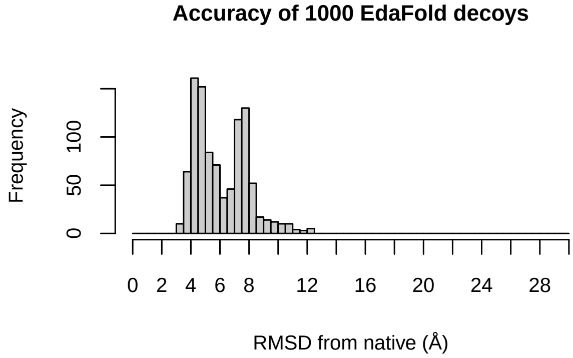

# 1ughI ( $\alpha + \beta$, 82 residues )

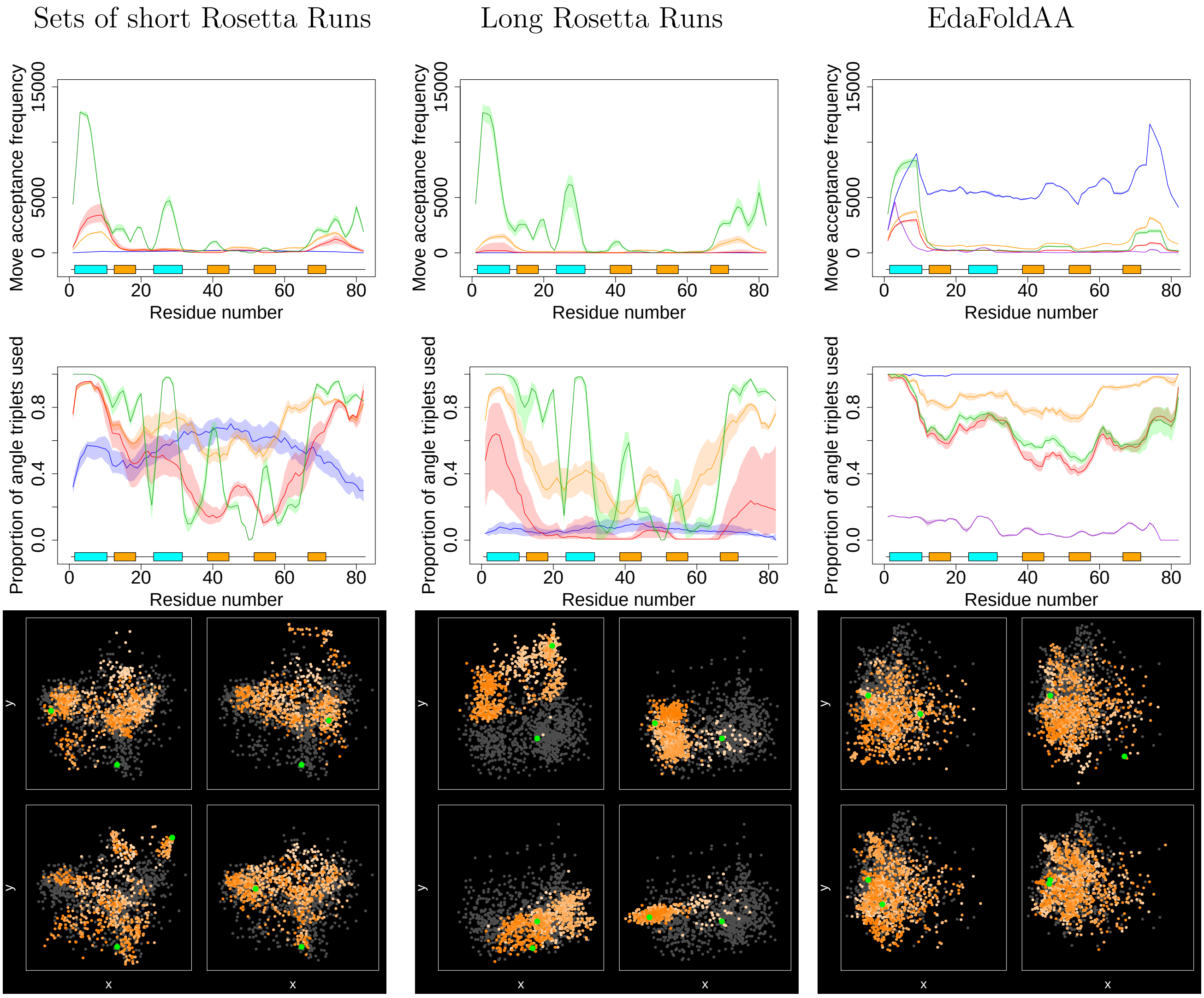


$f = 9.3702$ $f = 12.507$ $f = 11.187$

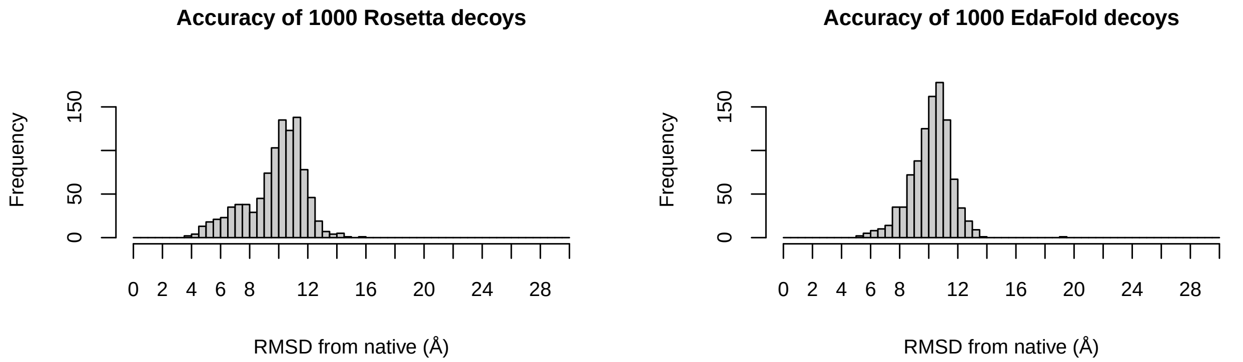

# 1opd ( $\alpha + \beta$, 85 residues )

Sets of short Rosetta Runs | Long Rosetta Runs | EdaFoldAA

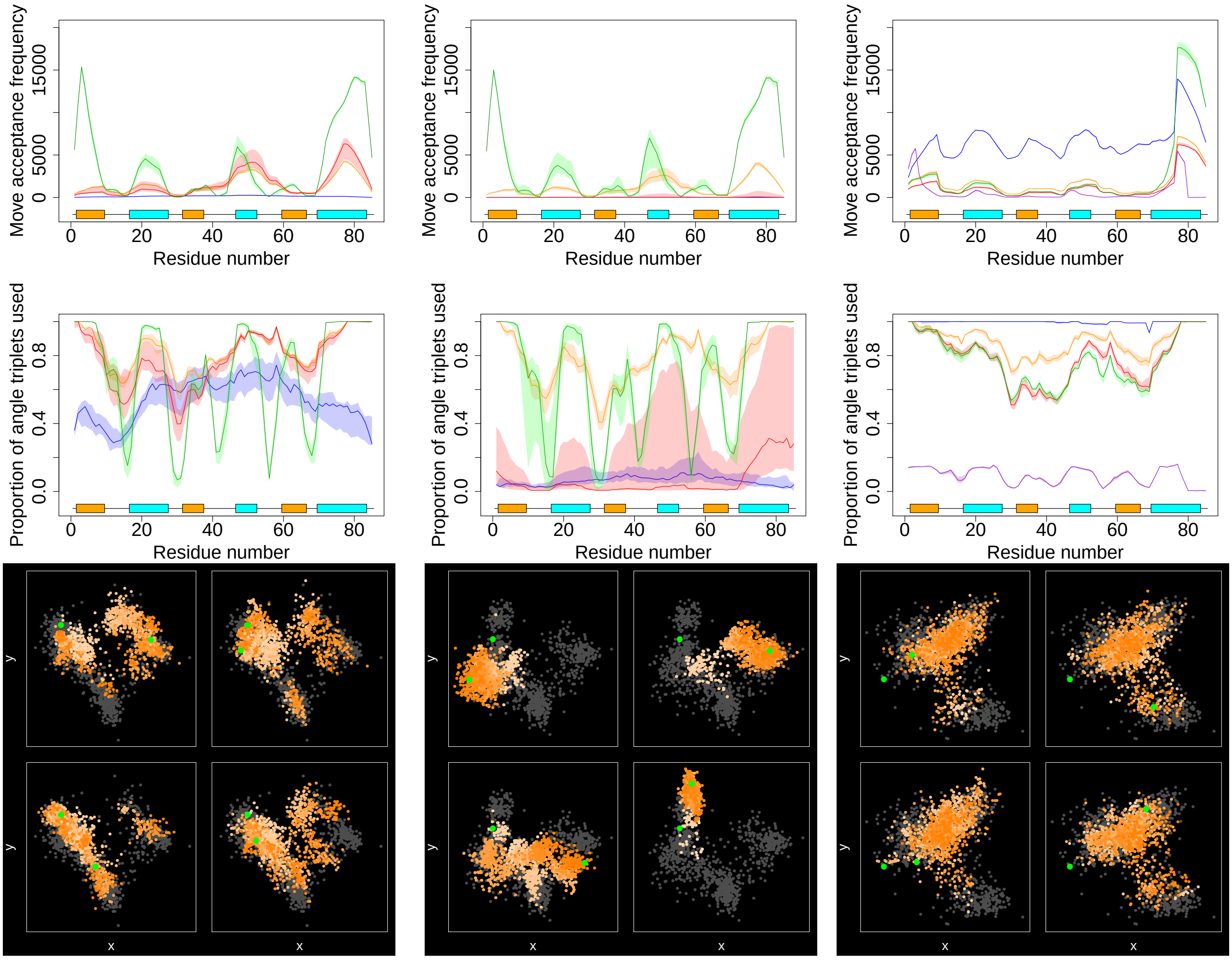


$f$ = 11.891 | $f$ = 15.461 | $f$ = 9.6847

**Accuracy of 1000 Rosetta decoys**

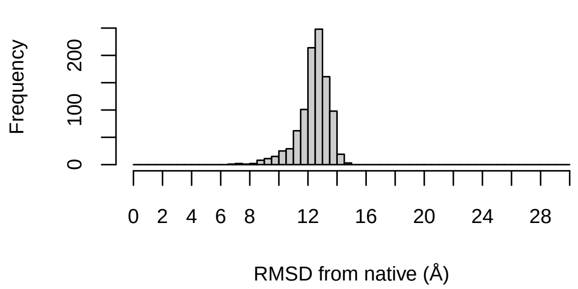


**Accuracy of 1000 EdaFold decoys**

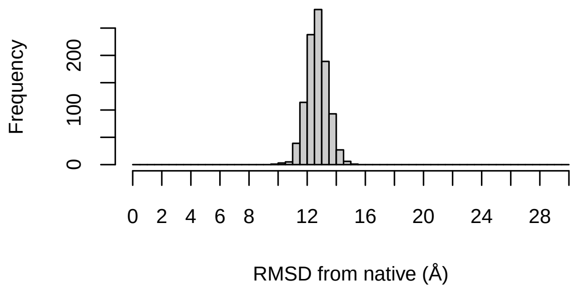

# 1tig ( $\alpha + \beta$, 88 residues )

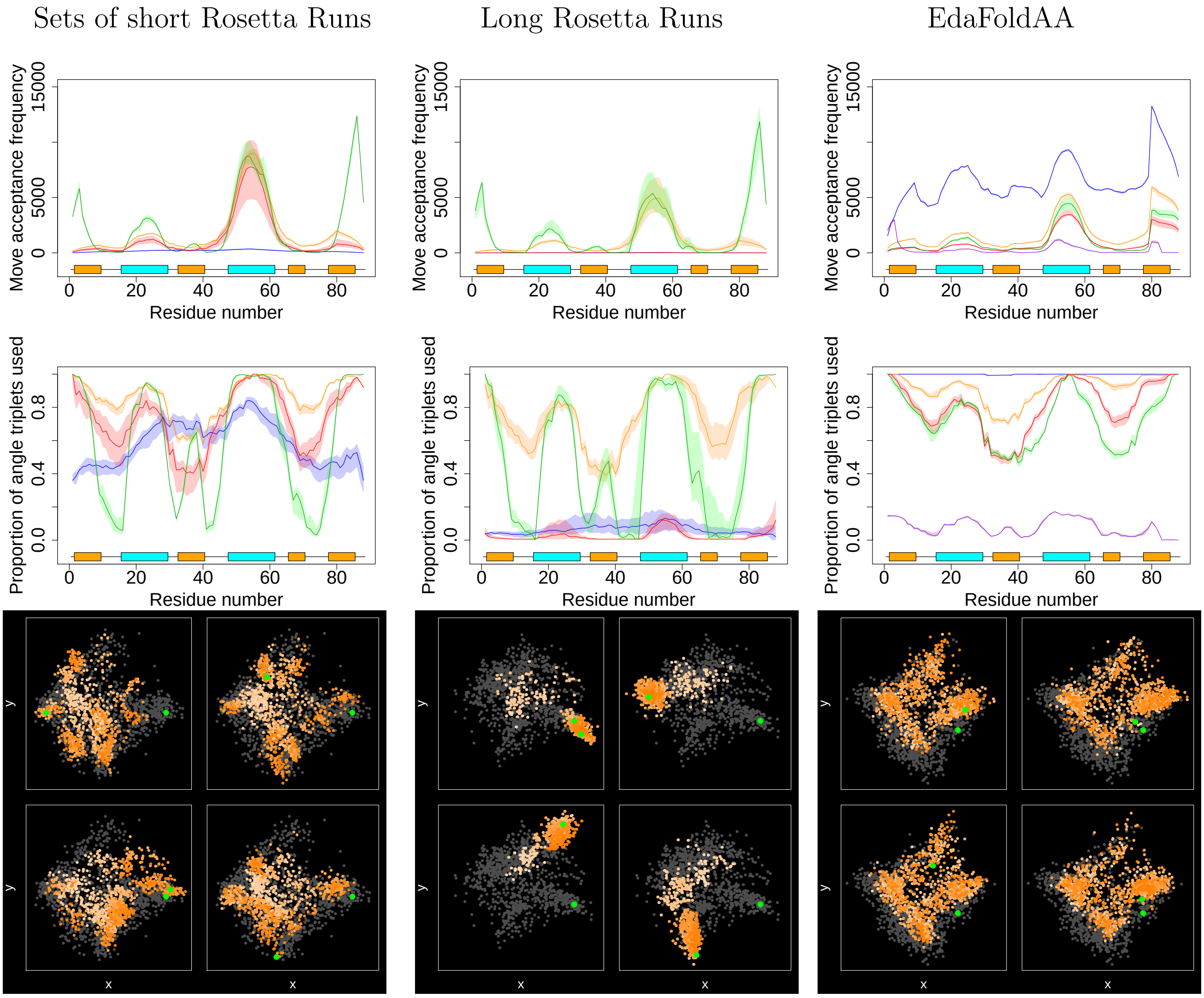


$f = 11.576$ $f = 16.714$ $f = 16.34$

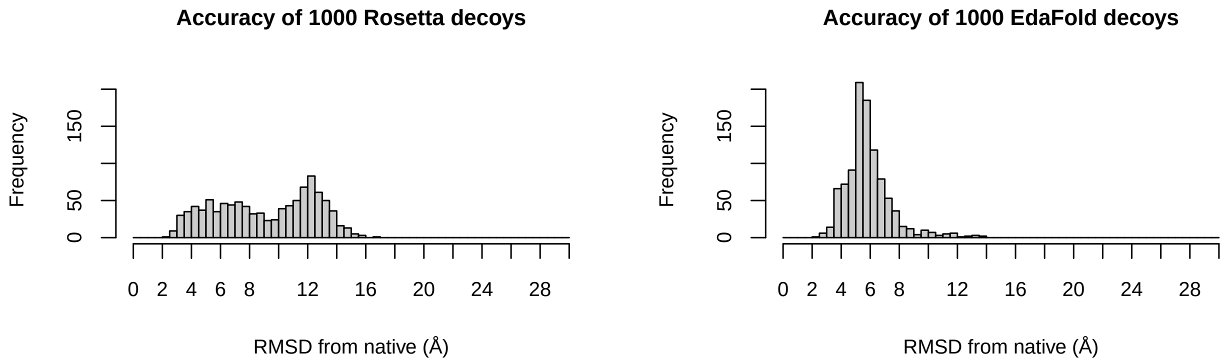

# 1urnA ( $\alpha + \beta$, 90 residues )

Sets of short Rosetta Runs | Long Rosetta Runs | EdaFoldAA

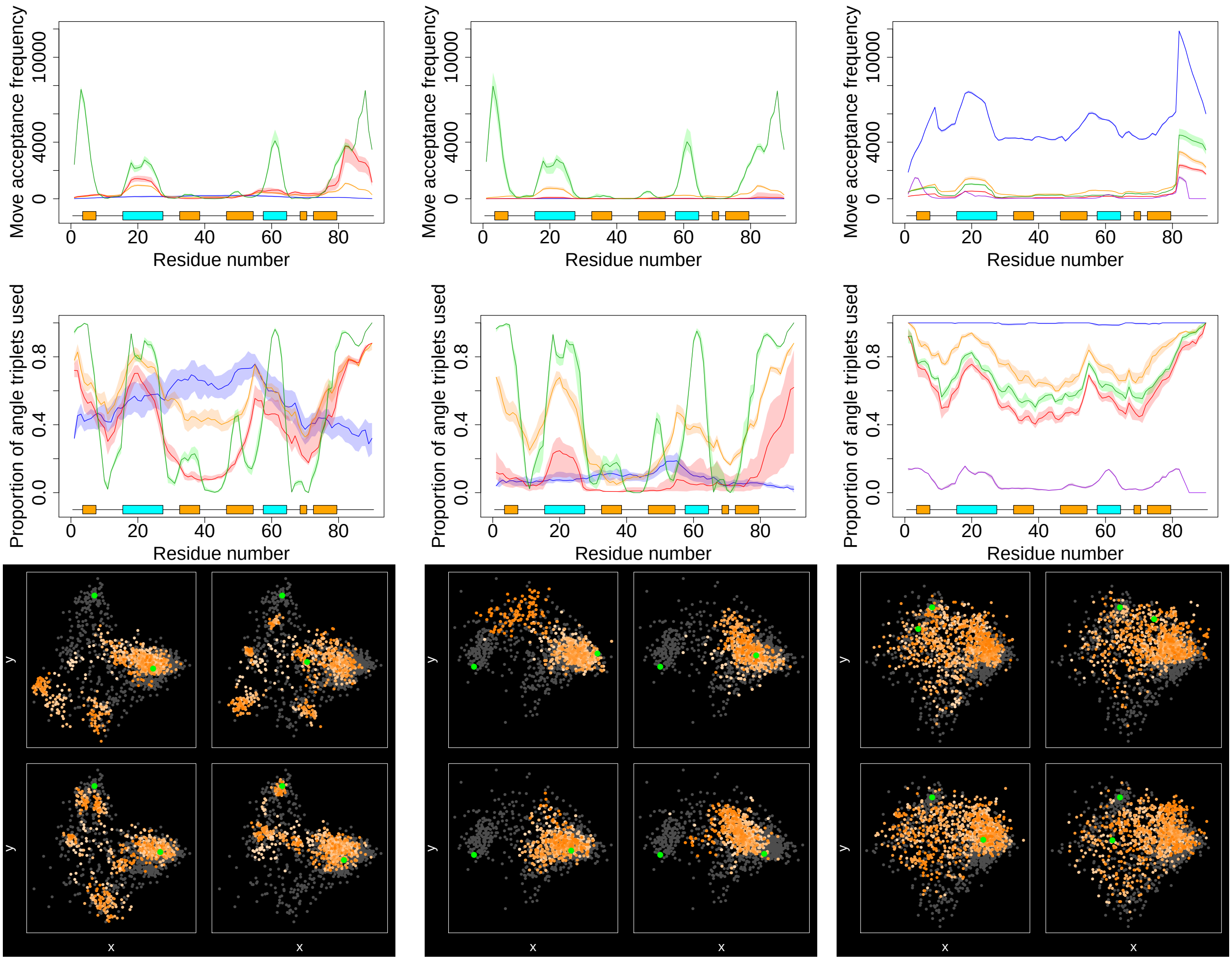


$f = 14.421$ $f = 10.745$ $f = 16.432$

**Accuracy of 1000 Rosetta decoys**

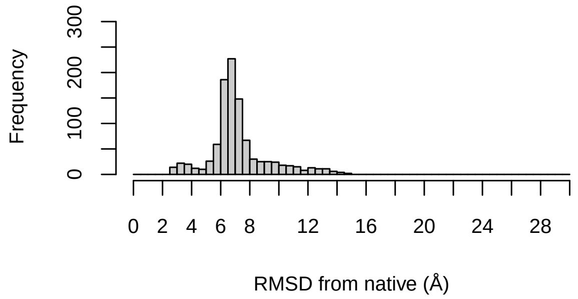


**Accuracy of 1000 EdaFold decoys**

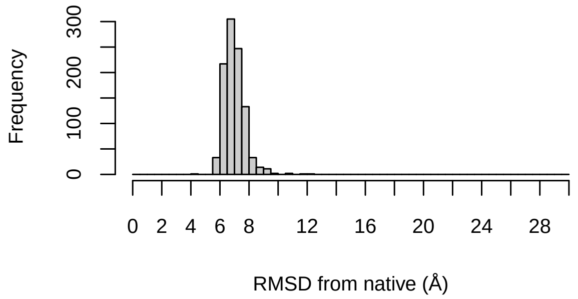

# 1louA ( $\alpha + \beta$, 92 residues )

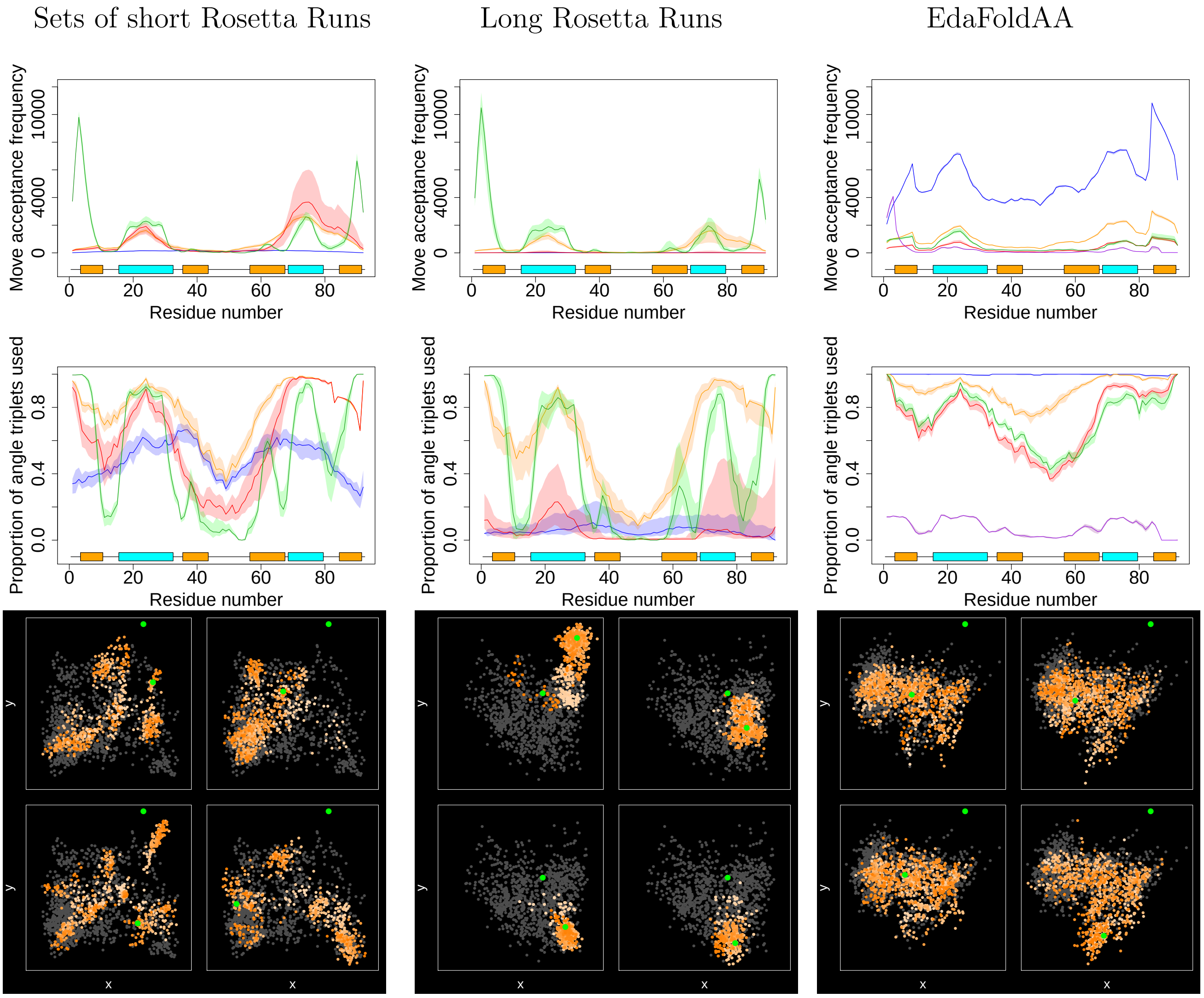


$f = 12.912$ $f = 14.658$ $f = 11.892$

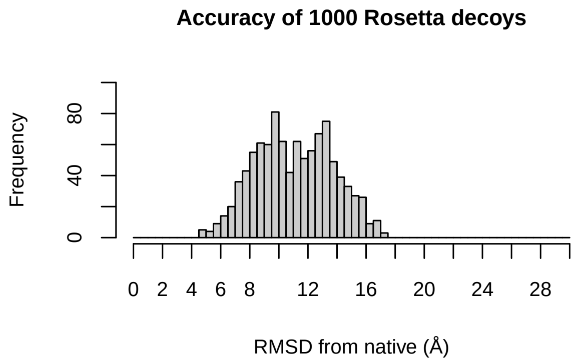


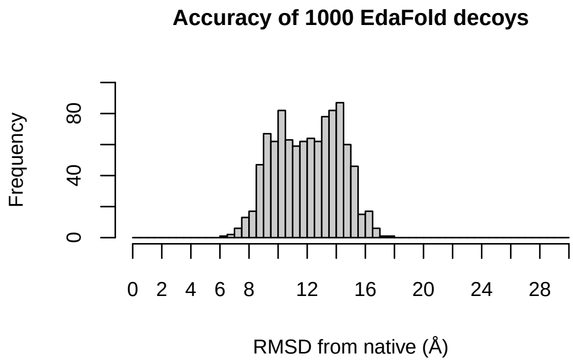

# 2acy ( $\alpha + \beta$, 98 residues )

Sets of short Rosetta Runs | Long Rosetta Runs | EdaFoldAA

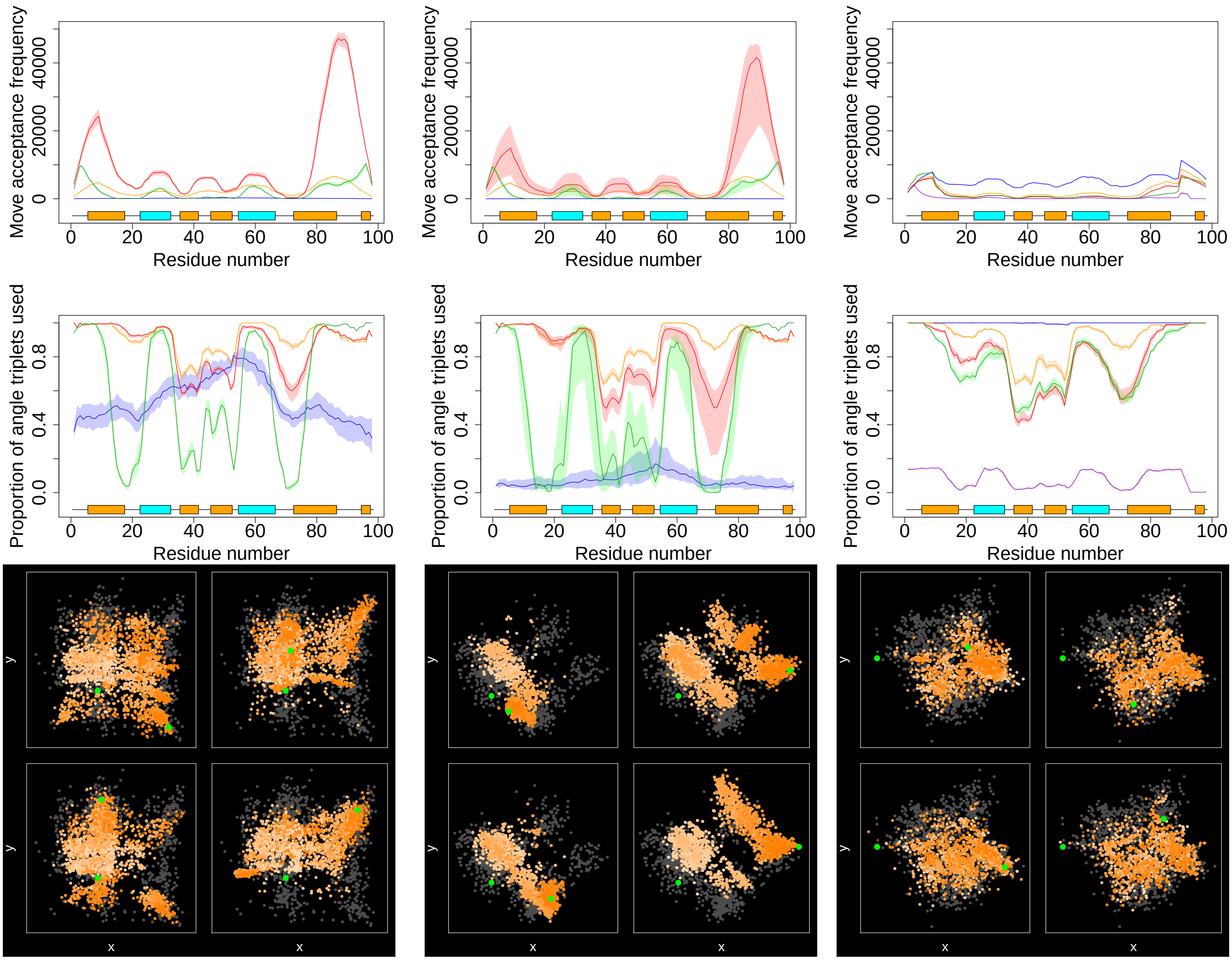


$f = 12.69$ | $f = 15.357$ | $f = 10.287$

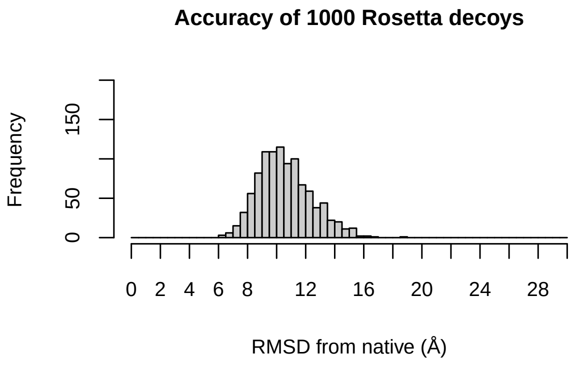


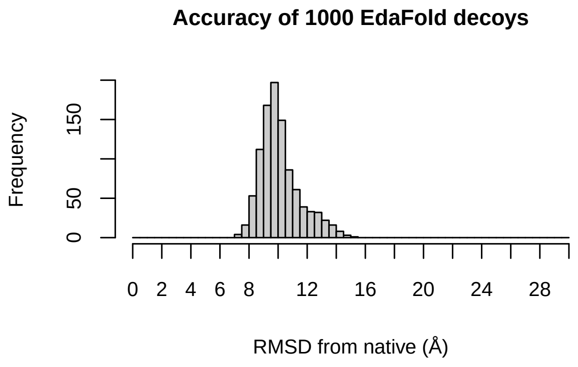

## 1bm8 ( $\alpha + \beta$, 99 residues )

Sets of short Rosetta Runs | Long Rosetta Runs | EdaFoldAA

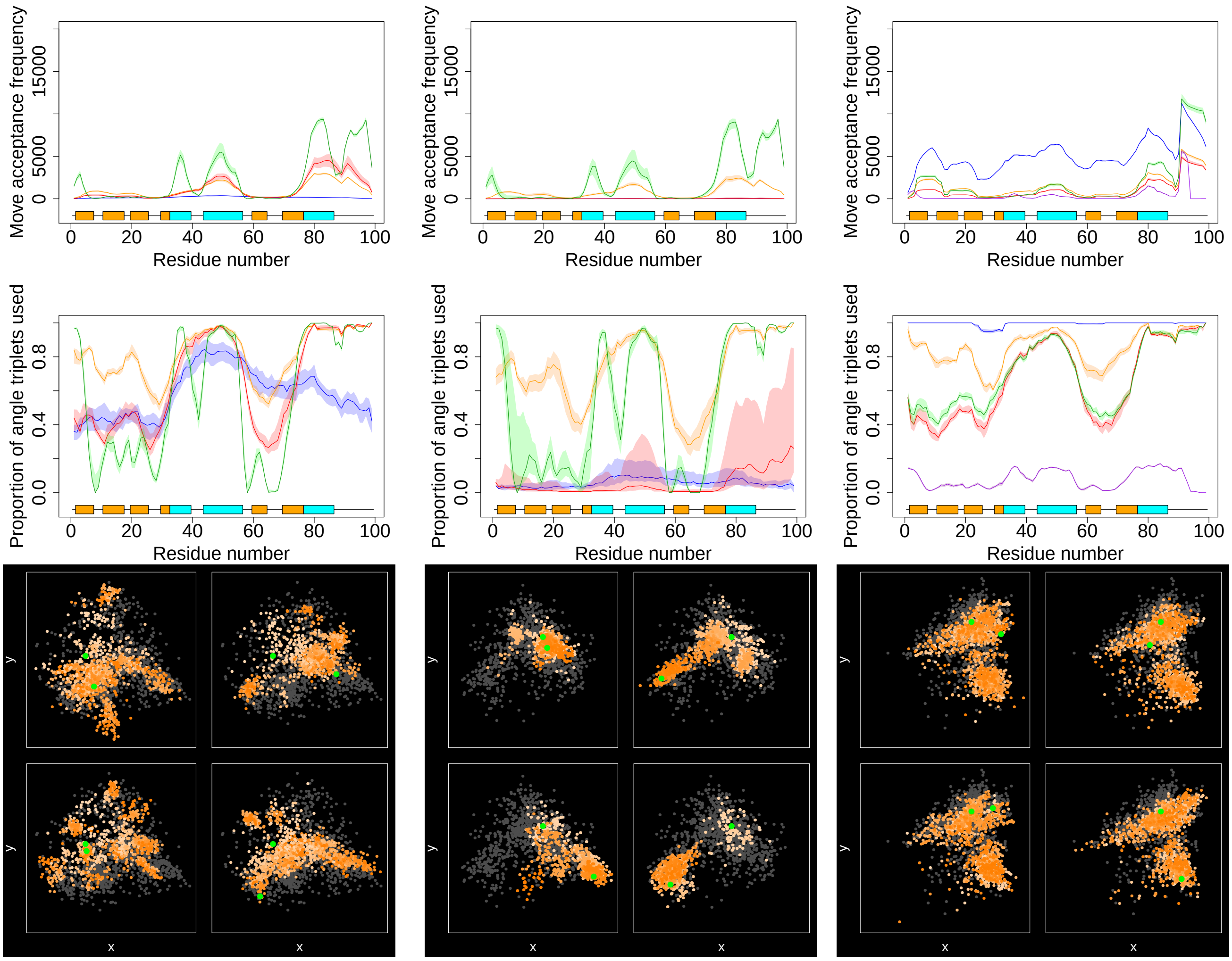


$f = 7.2699$ | $f = 12.97$ | $f = 10.362$

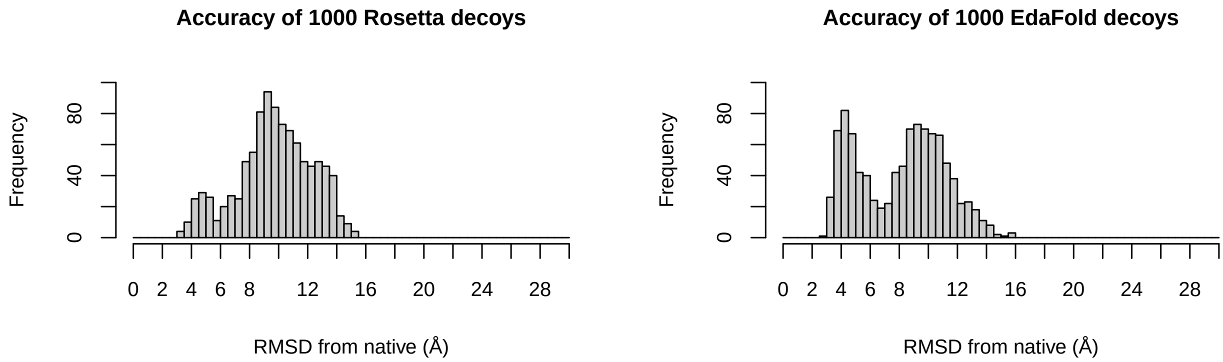

# 4ubpA ( $\alpha + \beta$, 100 residues )

Sets of short Rosetta Runs | Long Rosetta Runs | EdaFoldAA

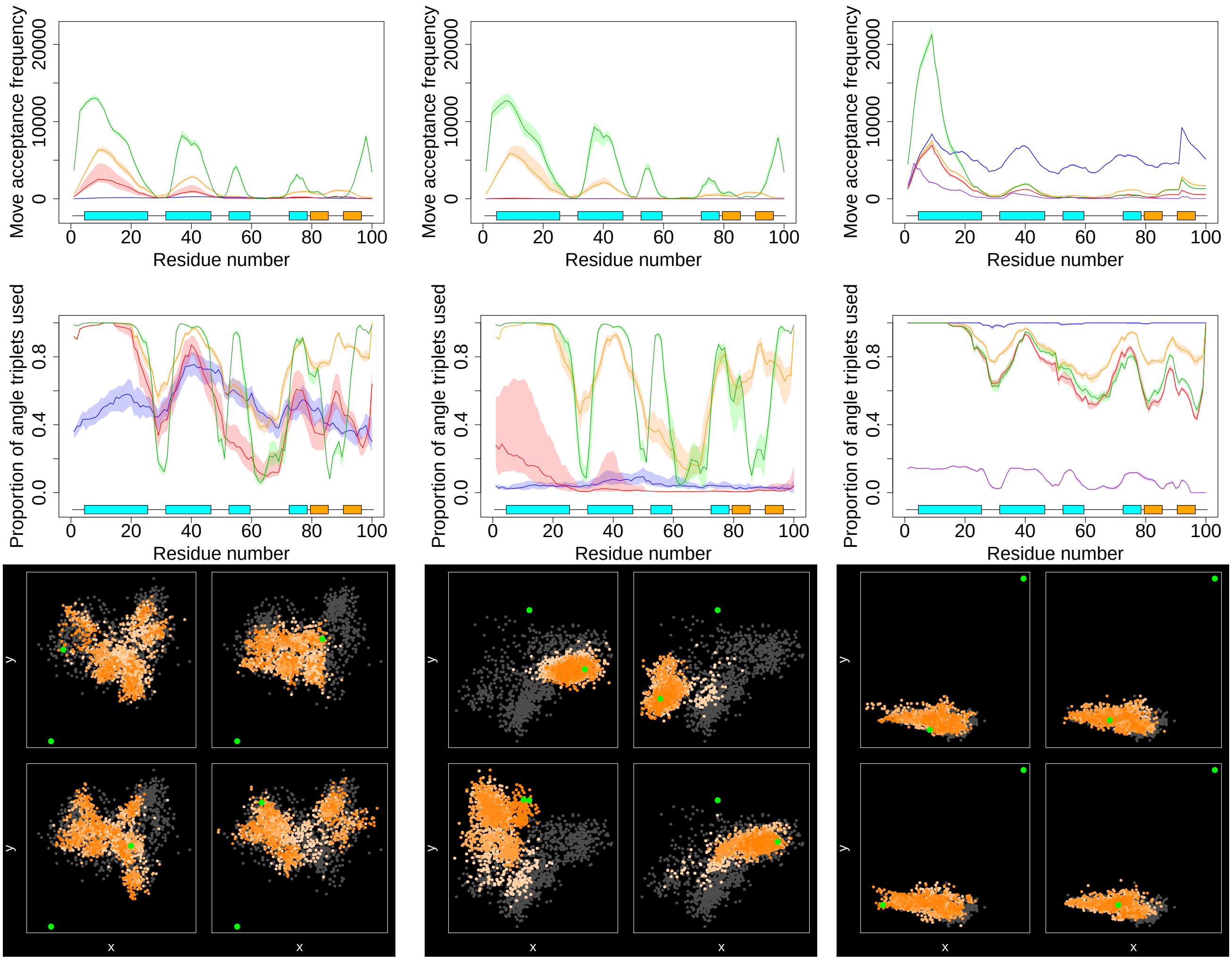


$f = 8.2921$ | $f = 11.465$ | $f = 7.7316$

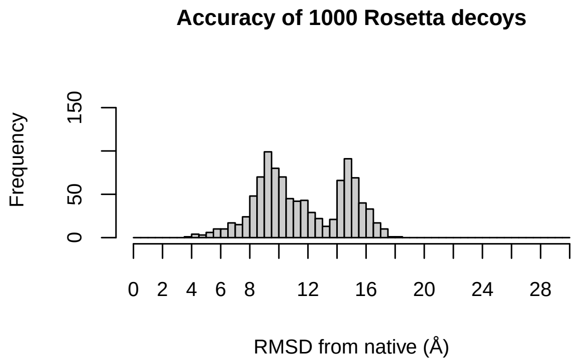


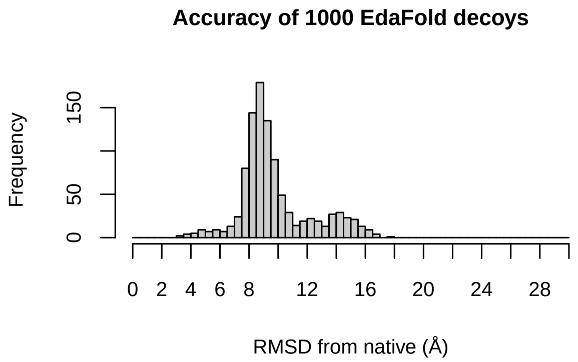

# 1ew4A ( $\alpha + \beta$, 106 residues )

Sets of short Rosetta Runs | Long Rosetta Runs | EdaFoldAA

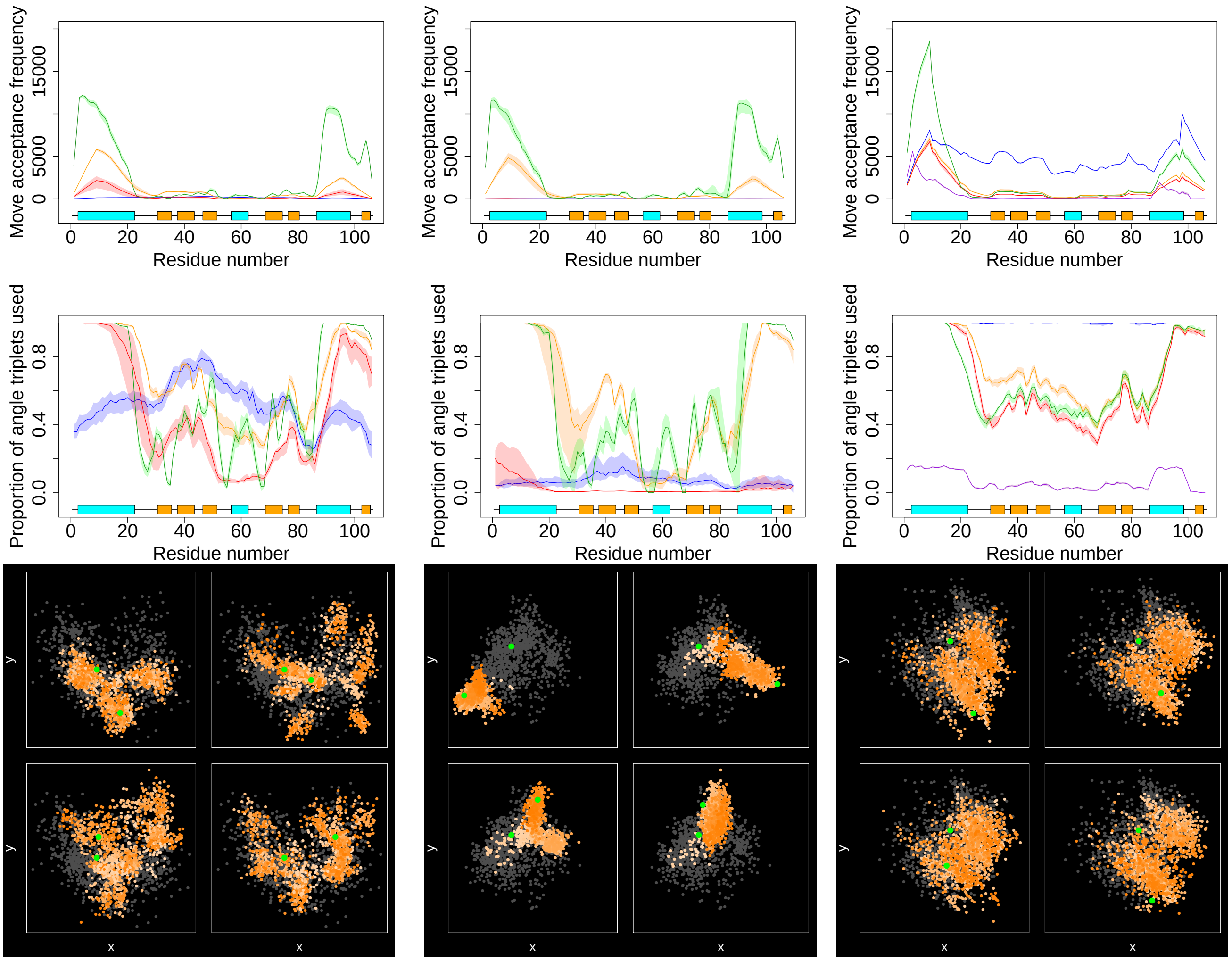


$f = 7.419$ | $f = 13.914$ | $f = 9.7681$

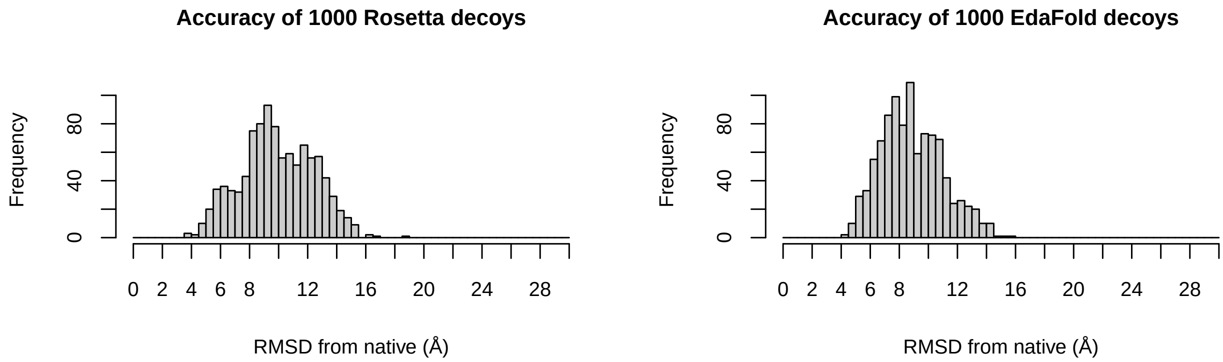

# 1fkb ( $\alpha + \beta$, 107 residues )

Sets of short Rosetta Runs | Long Rosetta Runs | EdaFoldAA

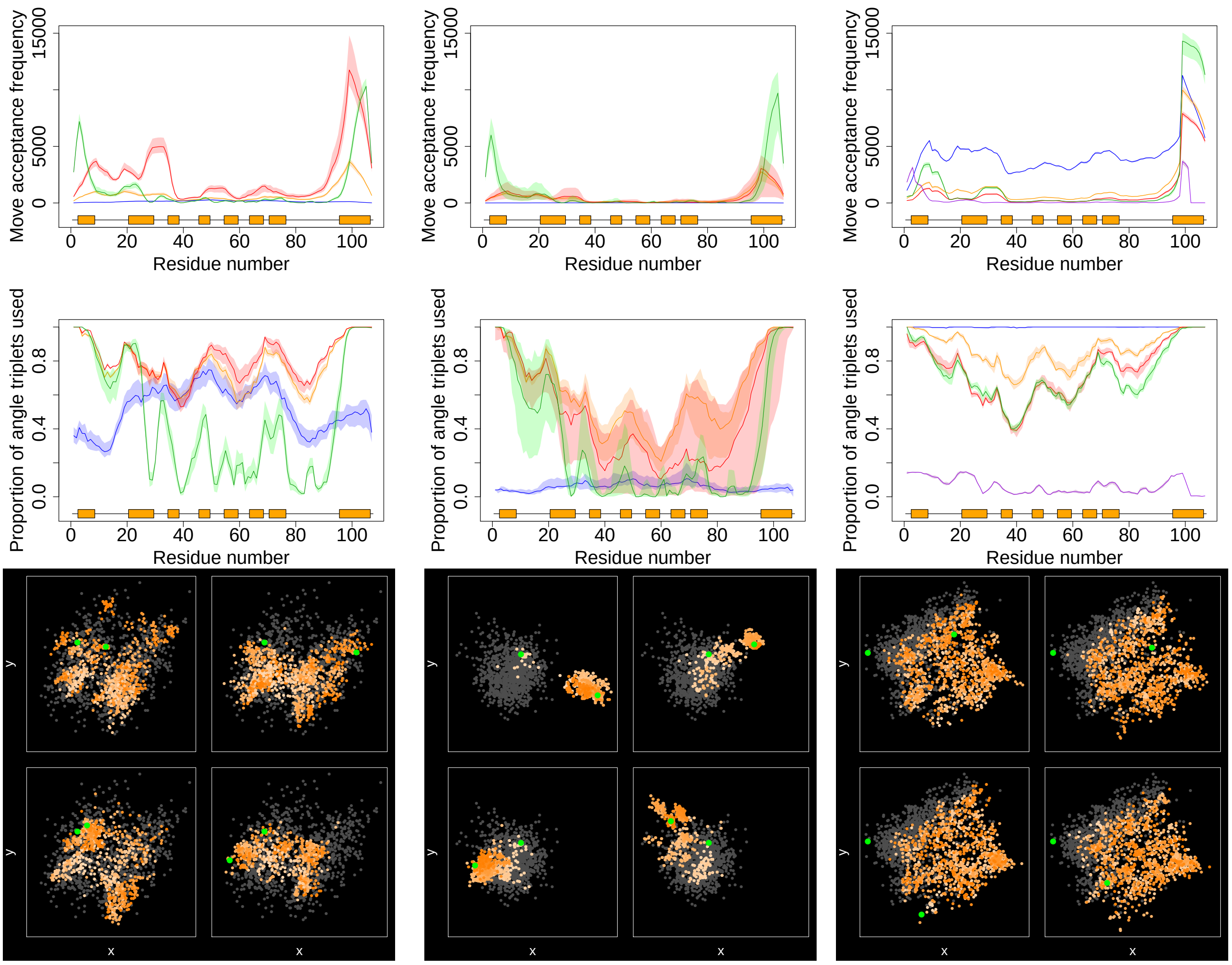


$f = 5.8105$ | $f = 12.718$ | $f = 7.8088$

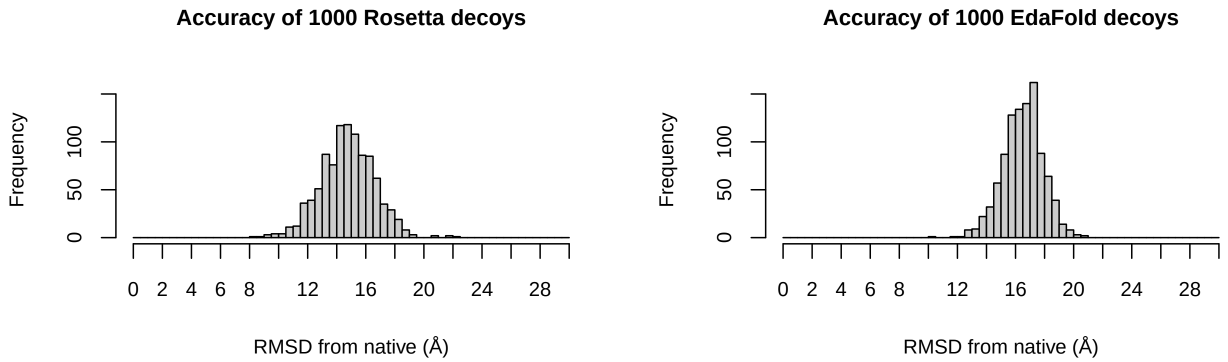

# 1kpeA ( $\alpha + \beta$, 108 residues )

Sets of short Rosetta Runs | Long Rosetta Runs | EdaFoldAA

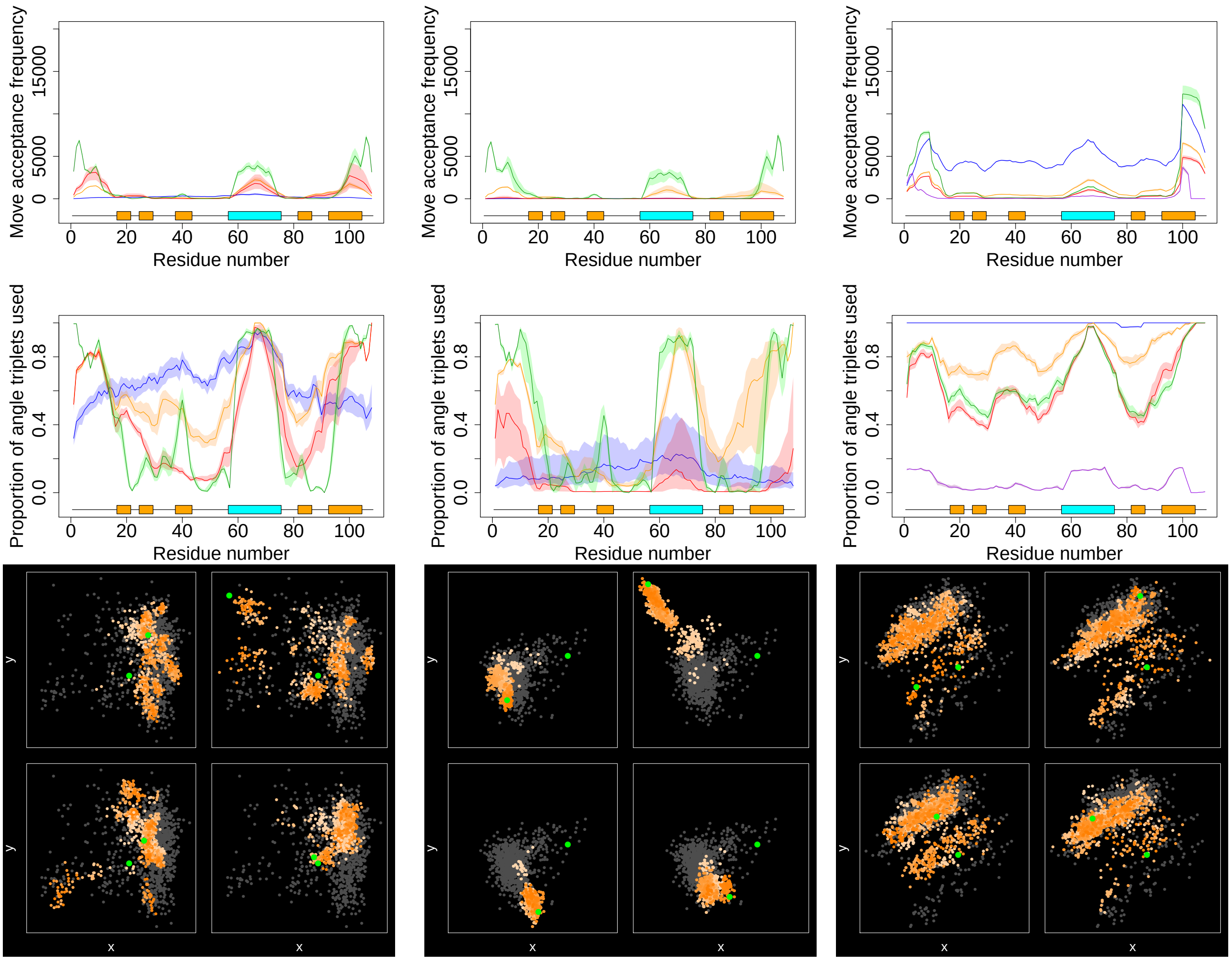


$f = 7.6517$ | $f = 12.52$ | $f = 8.4666$

**Accuracy of 1000 Rosetta decoys**

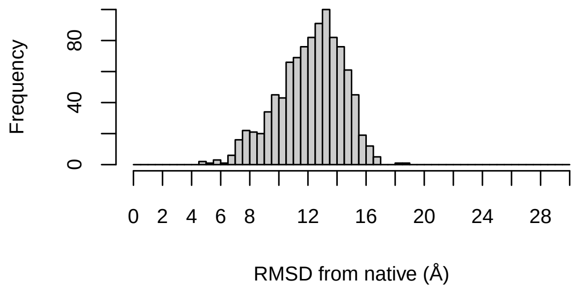


**Accuracy of 1000 EdaFold decoys**

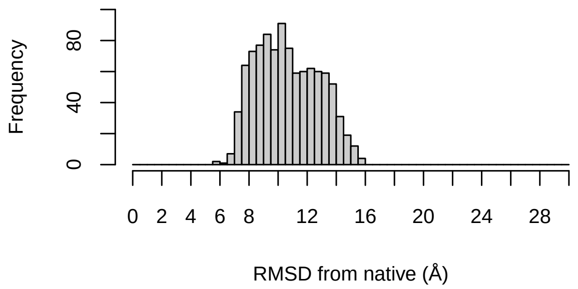

# 1rnbA ( $\alpha + \beta$, 109 residues )

Sets of short Rosetta Runs | Long Rosetta Runs | EdaFoldAA

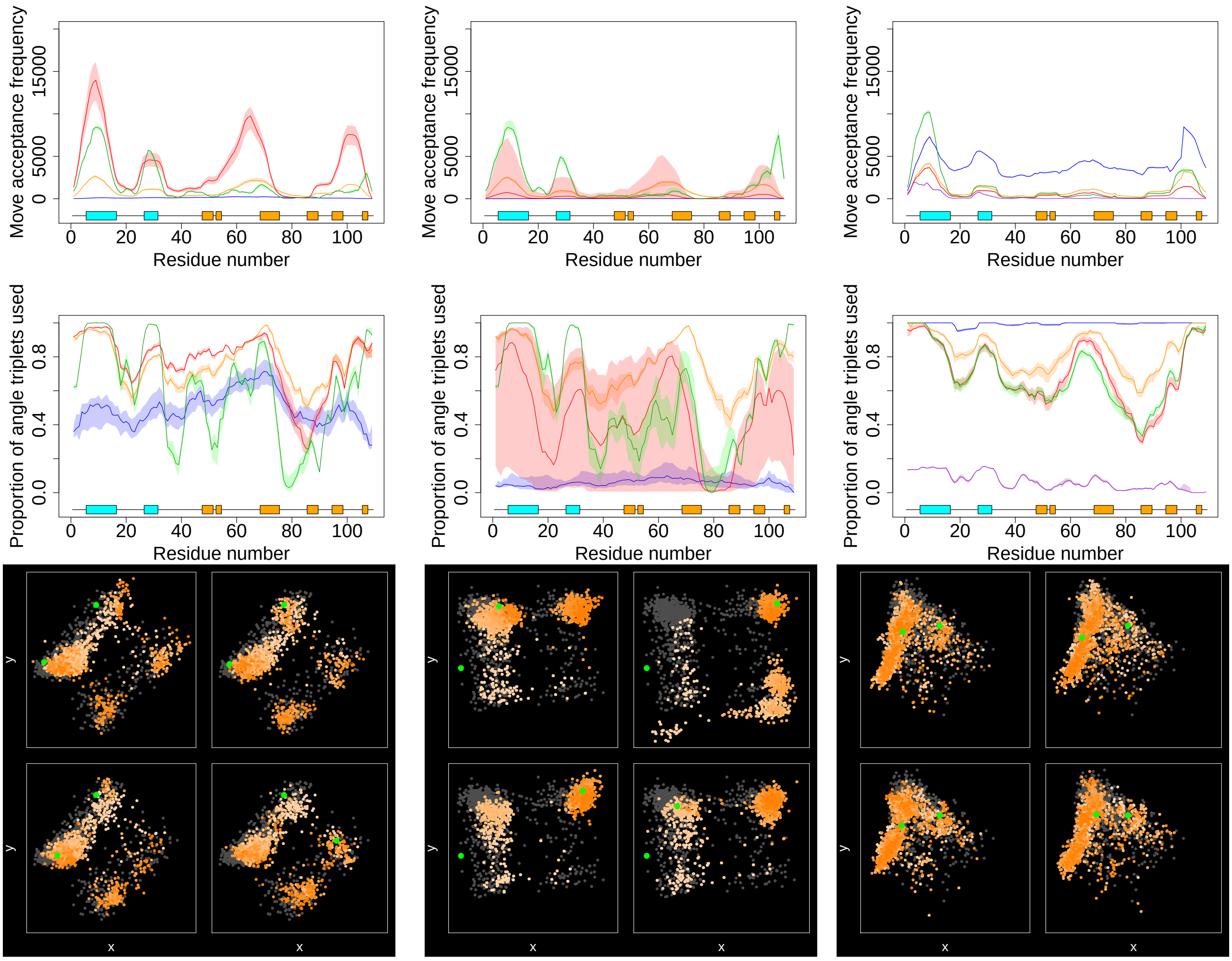


$f$ = 7.56 | $f$ = 11.209 | $f$ = 12.541

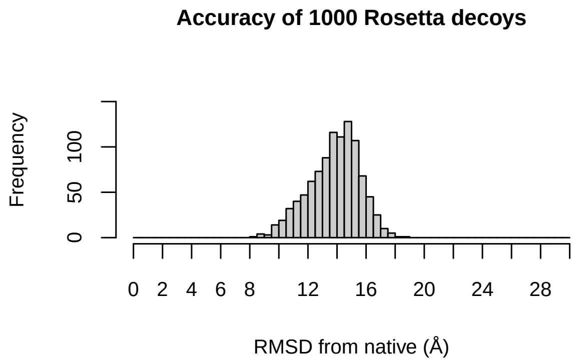


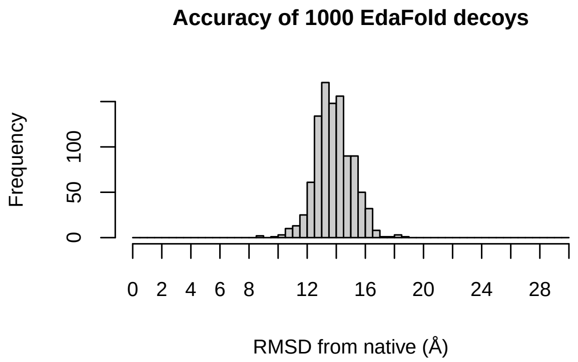

# 1dhn ( $\alpha + \beta$, 121 residues )

Sets of short Rosetta Runs | Long Rosetta Runs | EdaFoldAA

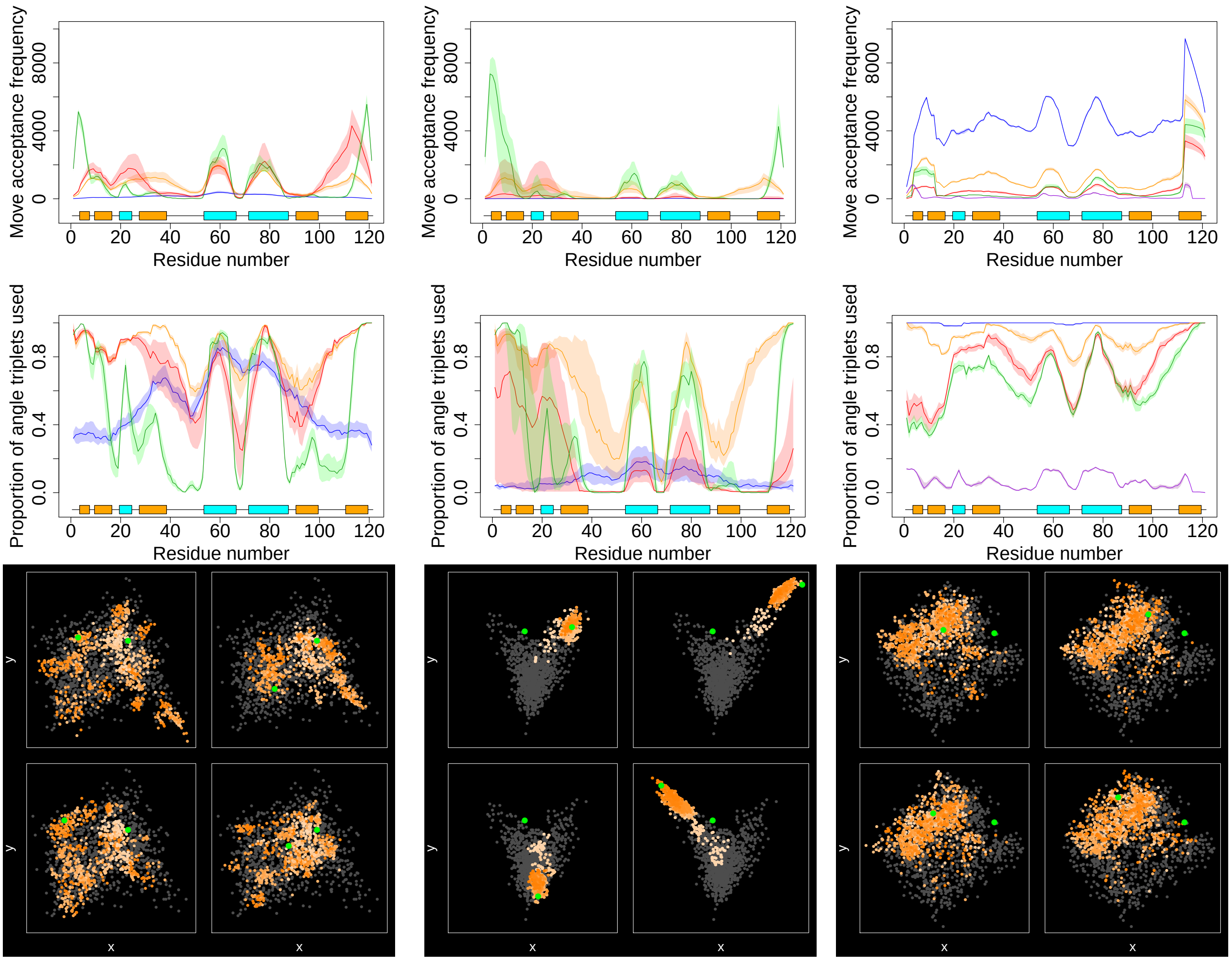


$f = 7.8429$ | $f = 21.008$ | $f = 6.8188$

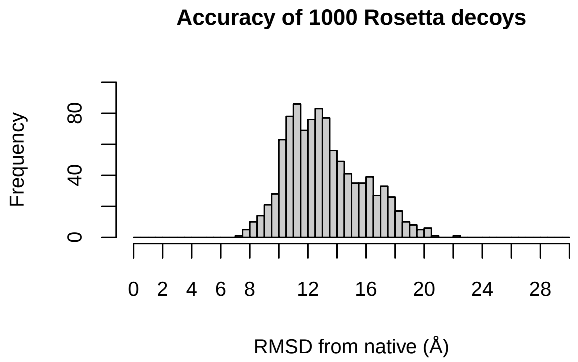


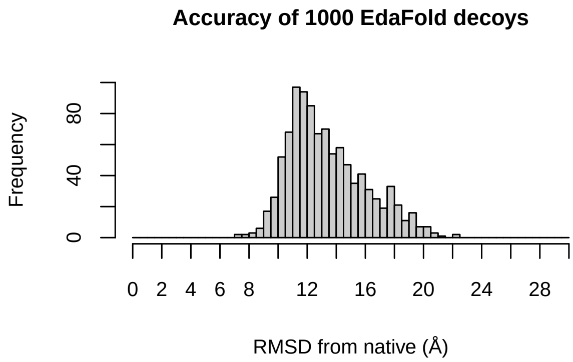

# 2vik ( $\alpha + \beta$, 122 residues )

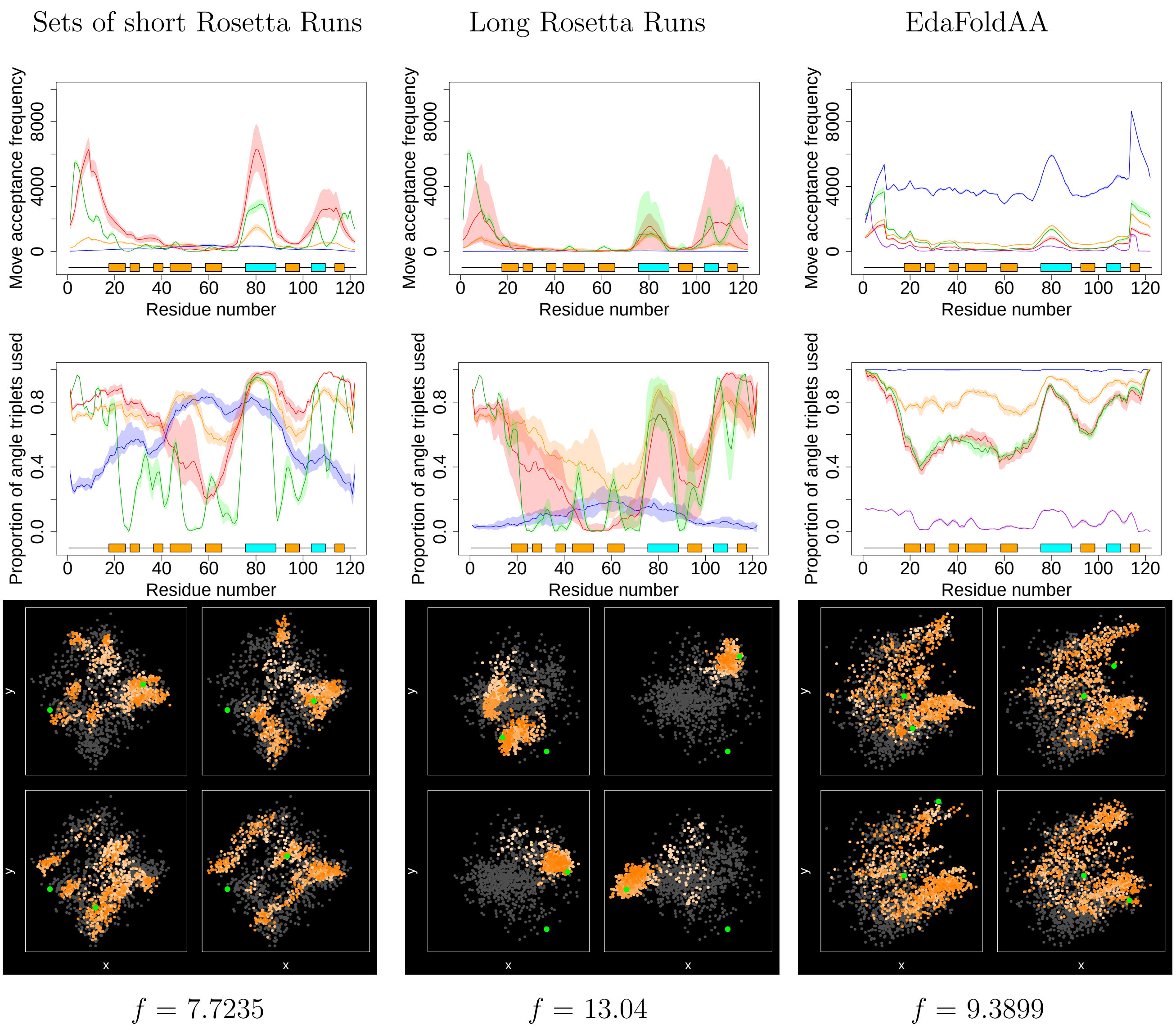


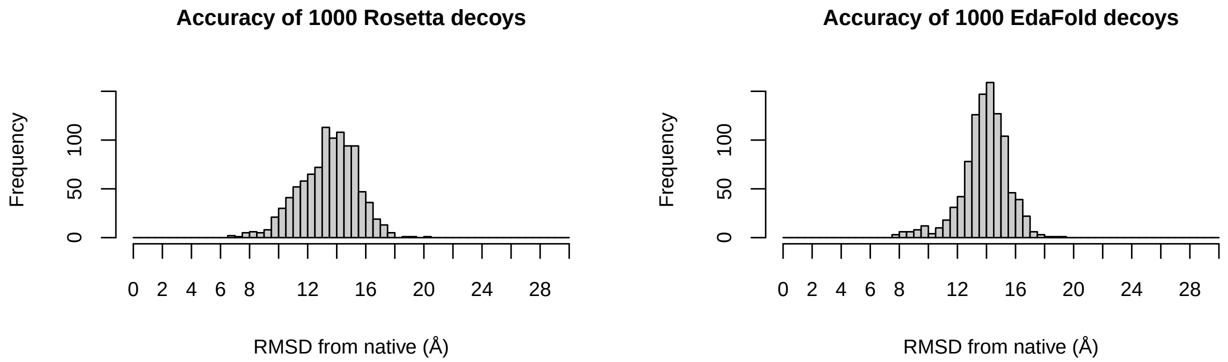

# 1acf ( $\alpha + \beta$, 125 residues )

Sets of short Rosetta Runs | Long Rosetta Runs | EdaFoldAA

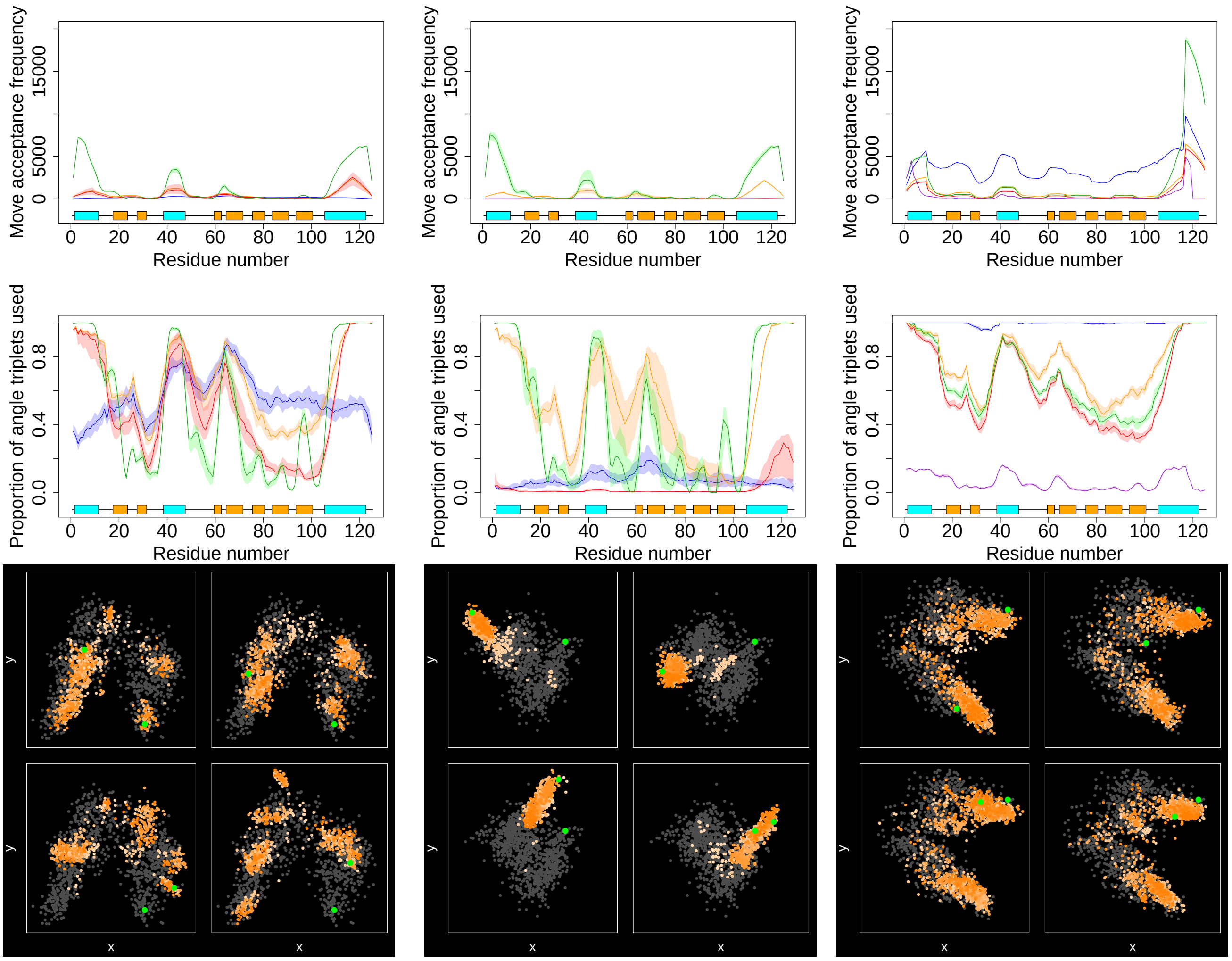


$f = 7.7629$ | $f = 13.418$ | $f = 11.64$

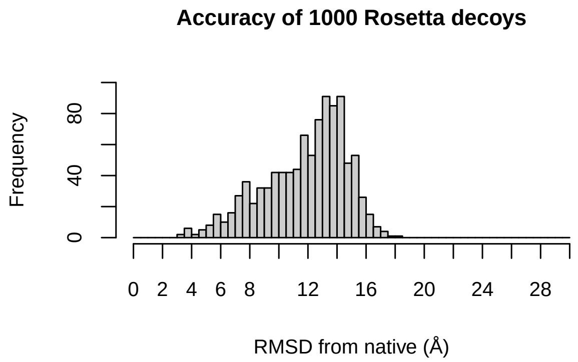


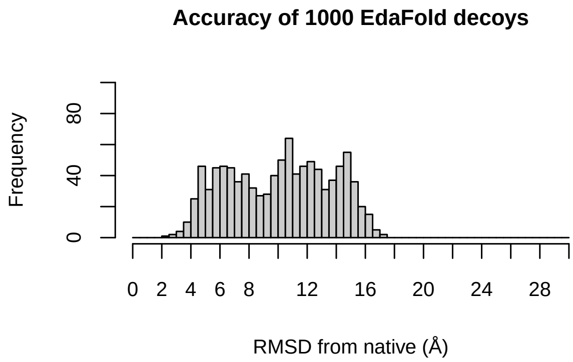

## 1bq9A ( all-$\beta$, 51 residues )

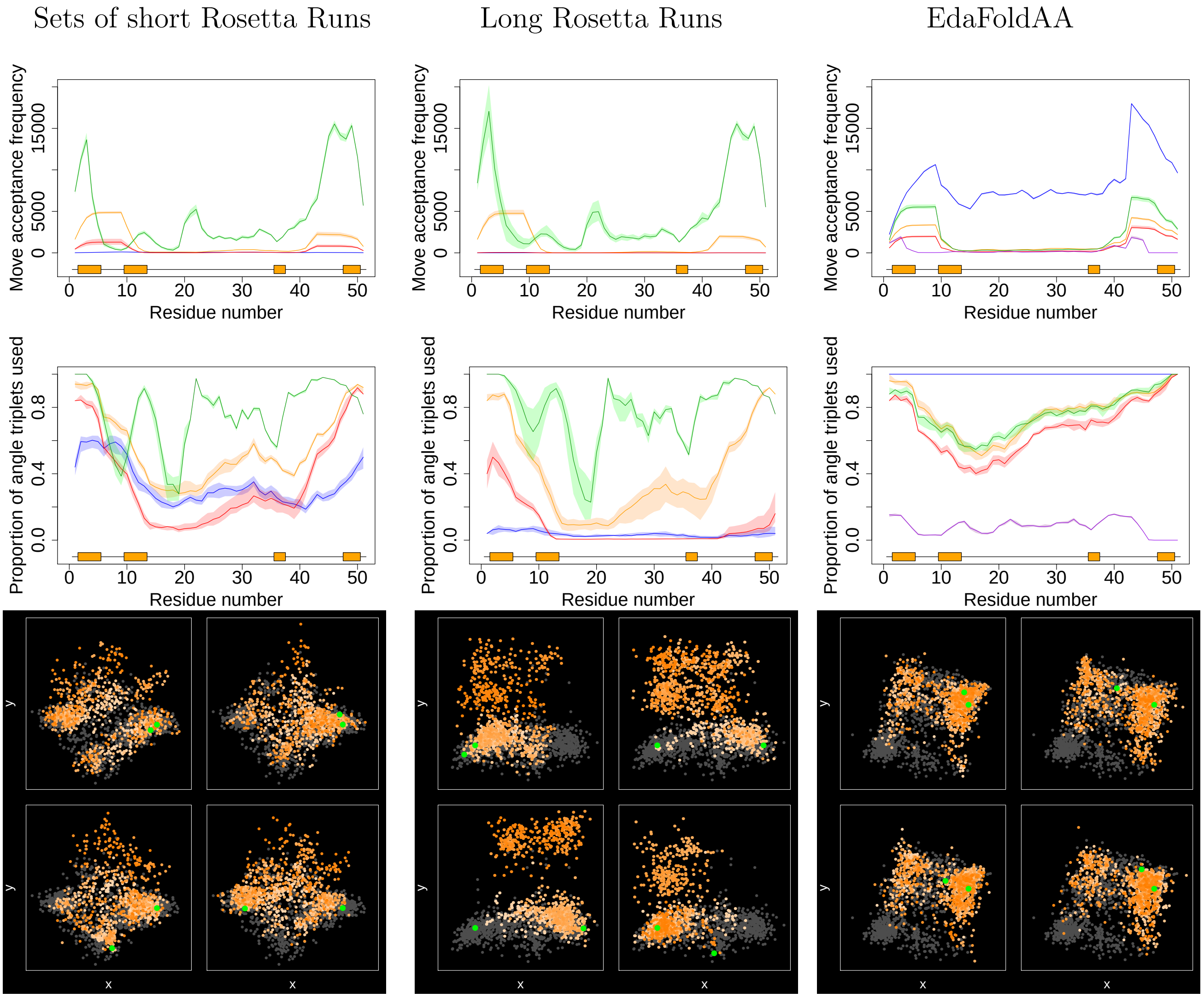


$f$ = 12.265 | $f$ = 13.664 | $f$ = 13.449

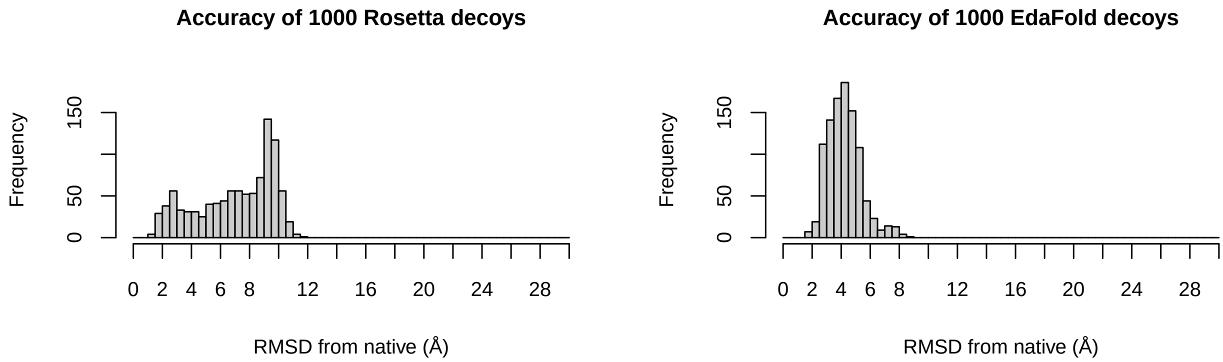

# 1vie ( all-$\beta$, 56 residues )

Sets of short Rosetta Runs | Long Rosetta Runs | EdaFoldAA

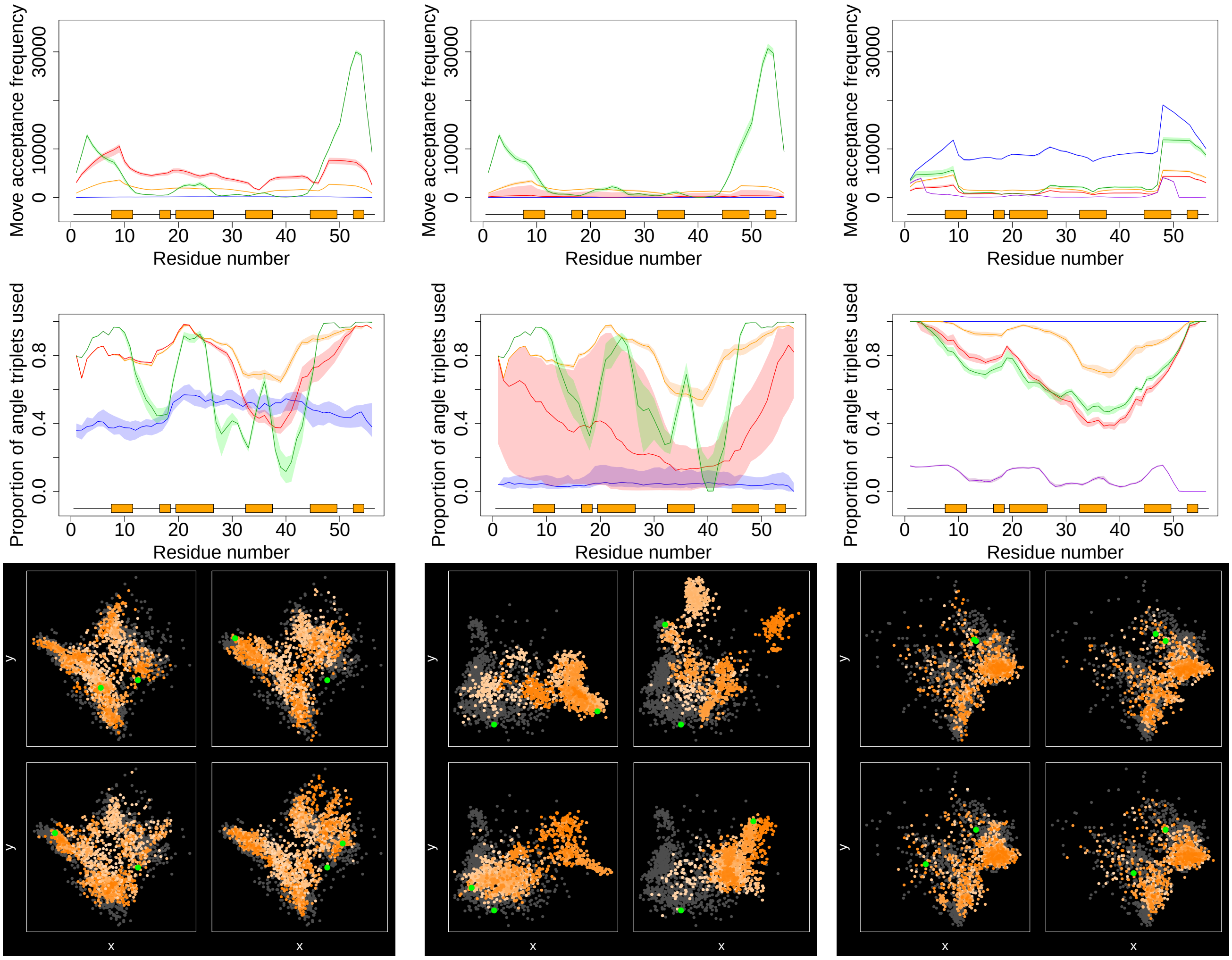


$f$ = 12.51 | $f$ = 12.227 | $f$ = 17.762

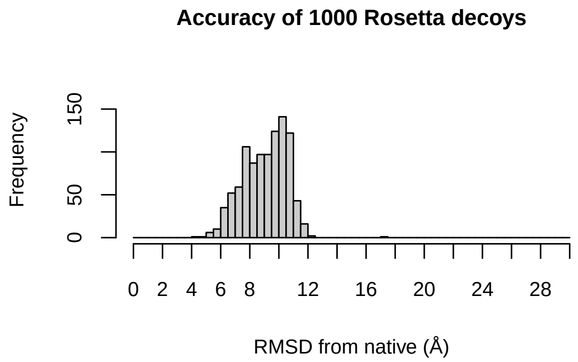


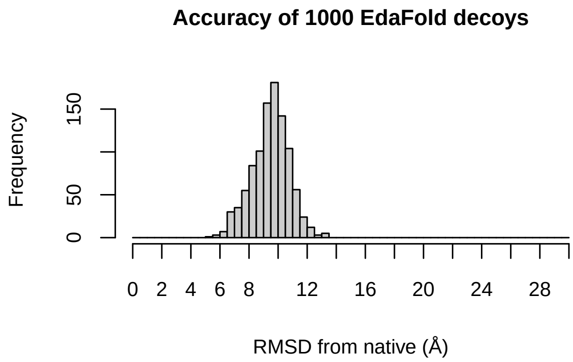

# 1bk2 ( all-$\beta$, 57 residues )

Sets of short Rosetta Runs | Long Rosetta Runs | EdaFoldAA

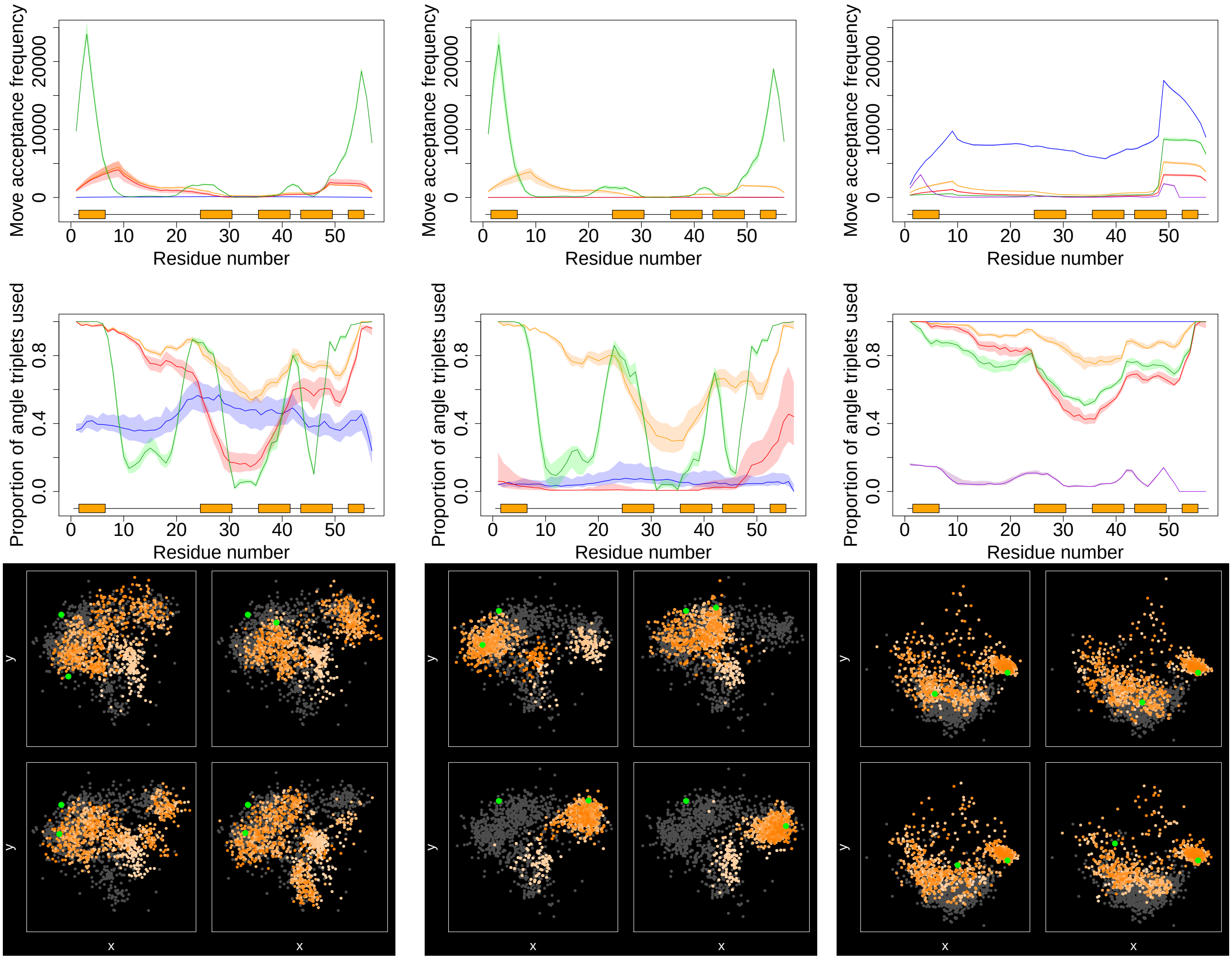


$f$ = 15.116 | $f$ = 19.133 | $f$ = 31.796

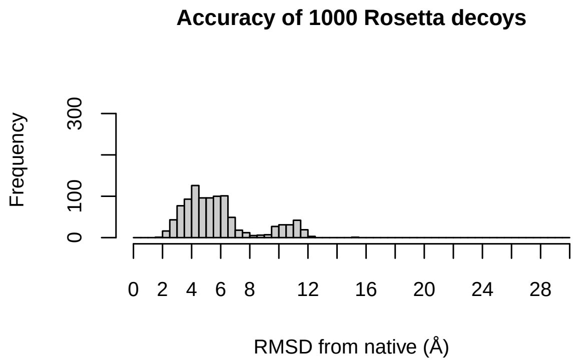


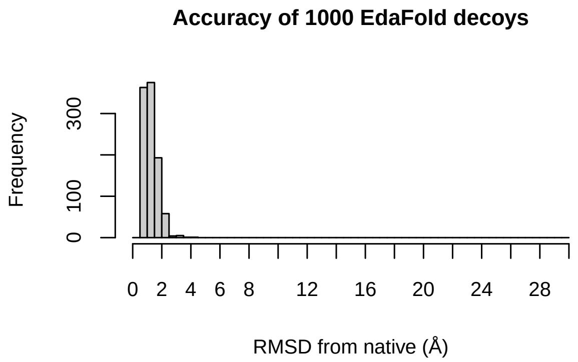

# 1shfA ( all-$\beta$, 59 residues )

Sets of short Rosetta Runs | Long Rosetta Runs | EdaFoldAA

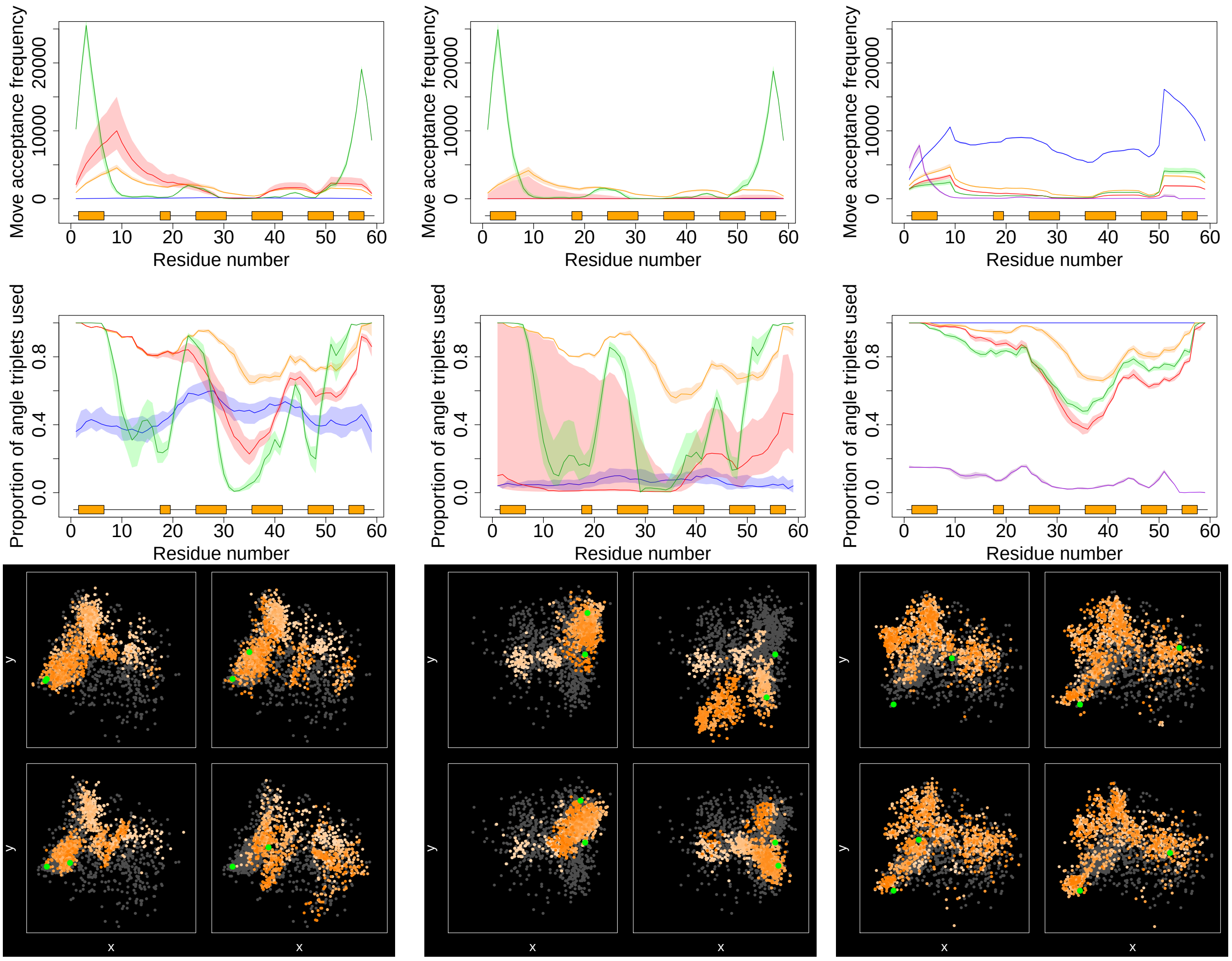


$f$ = 12.619 | $f$ = 16.34 | $f$ = 20.7

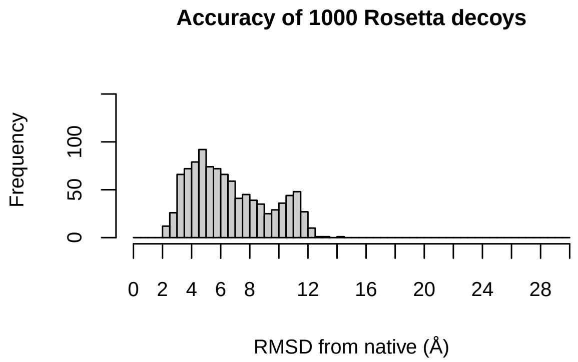


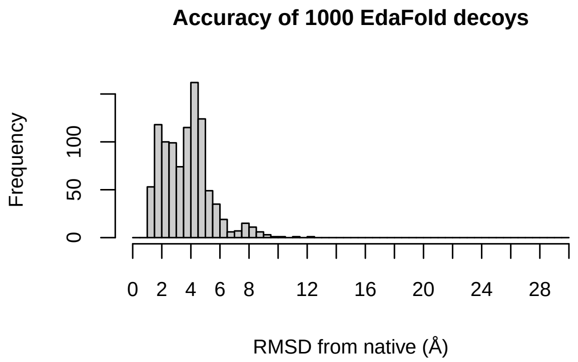

# 1c8cA ( all-$\beta$, 62 residues )

Sets of short Rosetta Runs | Long Rosetta Runs | EdaFoldAA

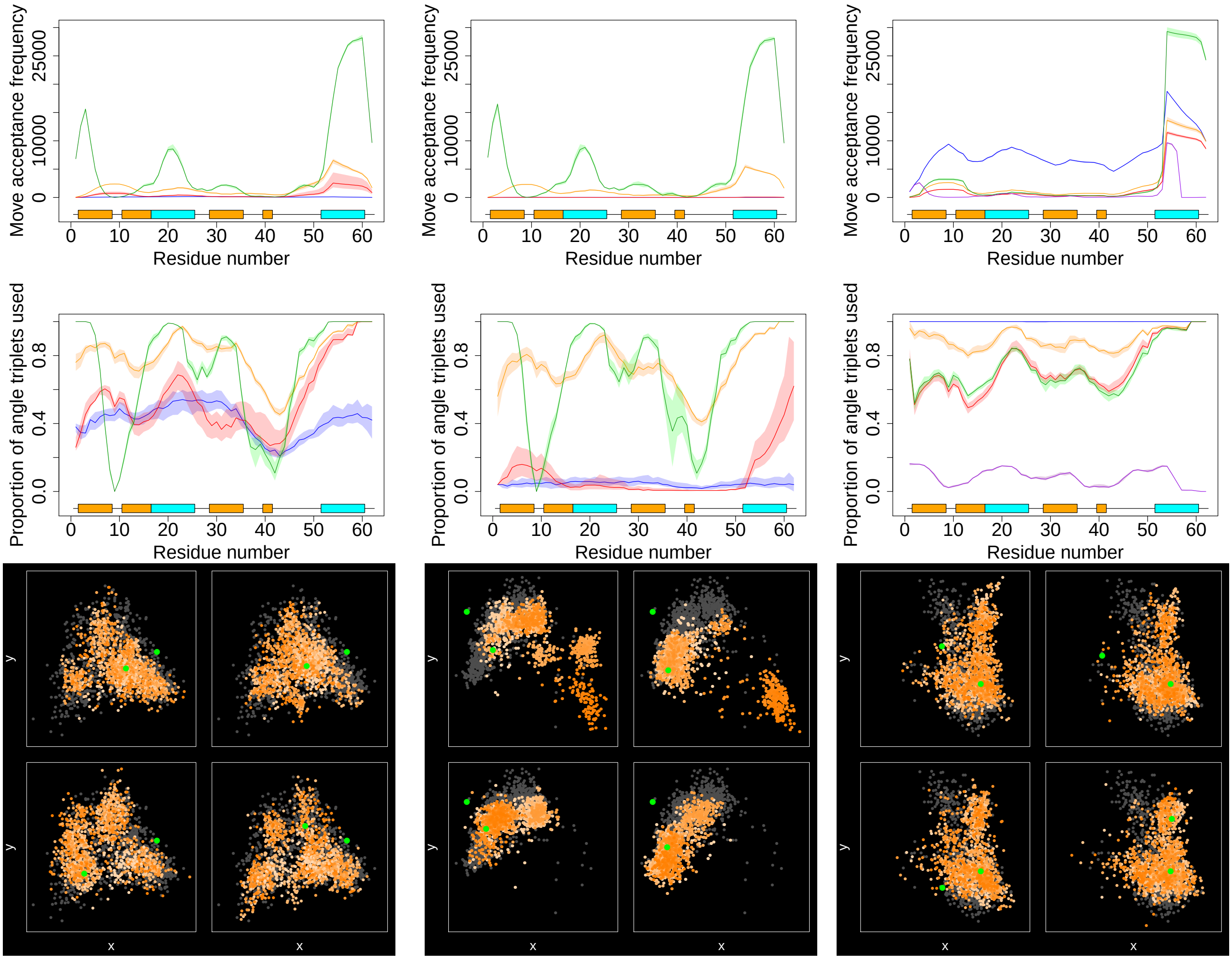


$f$ = 10.018 | $f$ = 14.788 | $f$ = 10.502

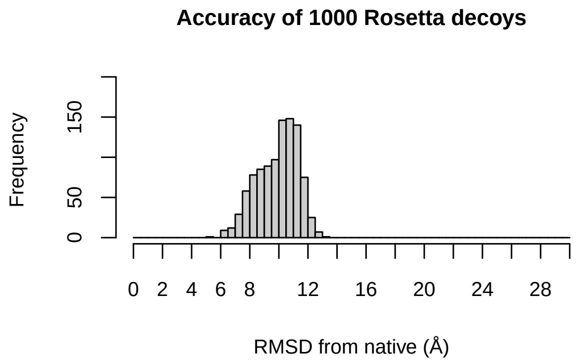


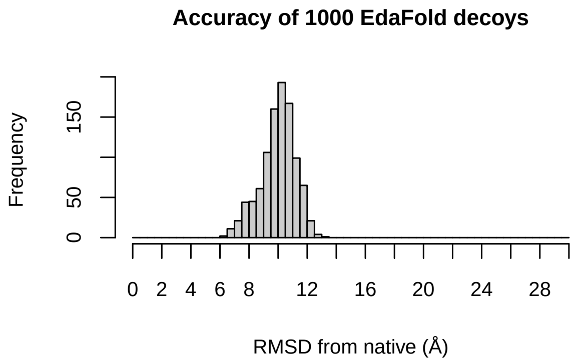

# 1c9oA ( all-$\beta$, 66 residues )

Sets of short Rosetta Runs | Long Rosetta Runs | EdaFoldAA

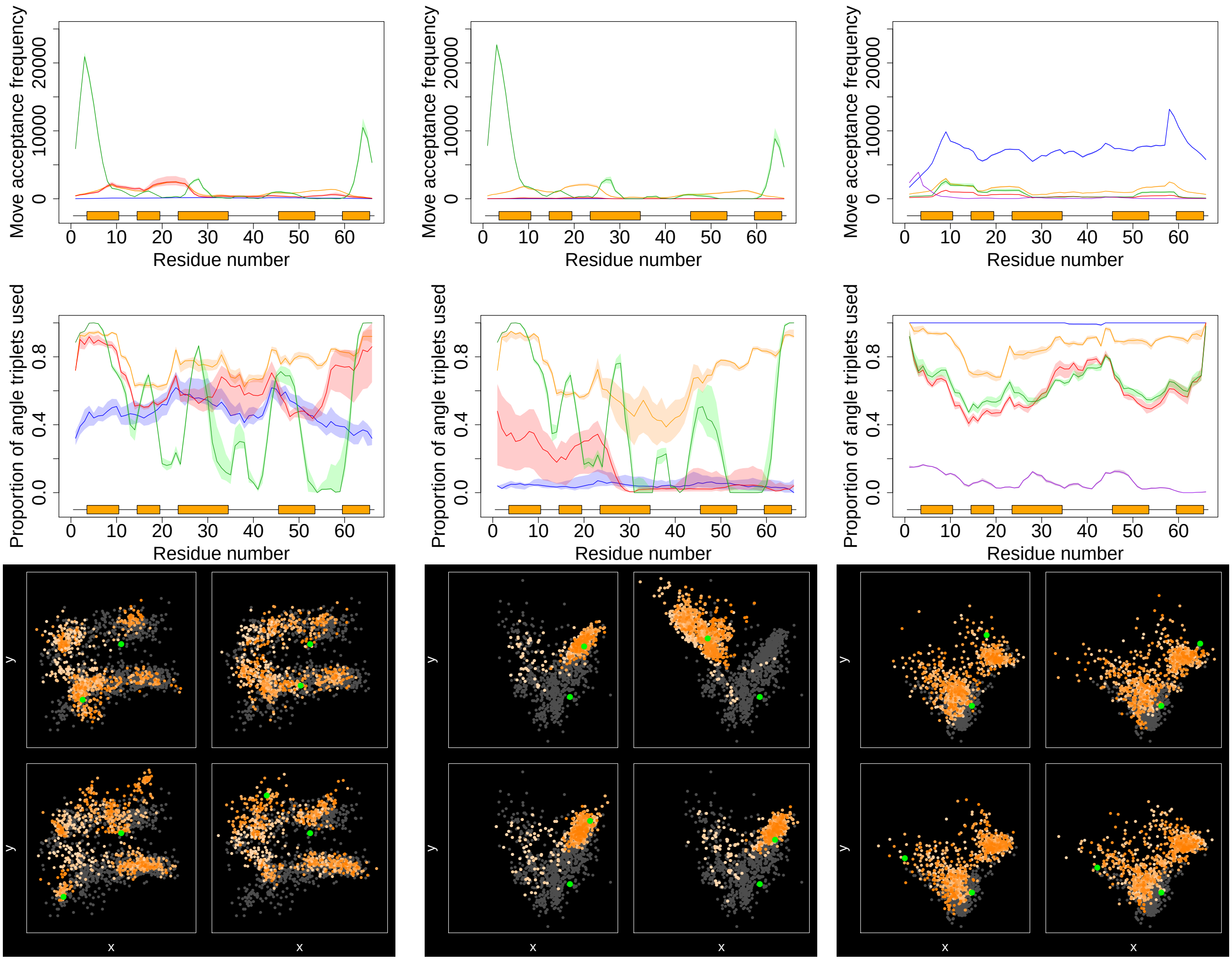


$f = 11.186$ | $f = 17.281$ | $f = 17.697$

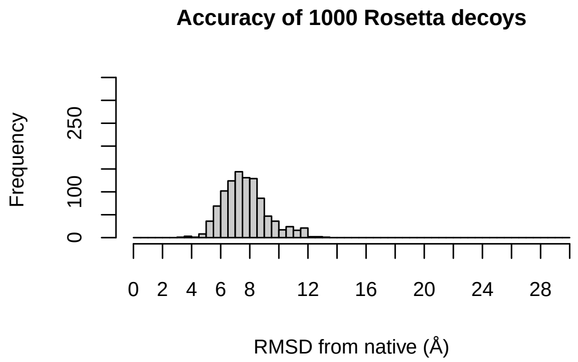


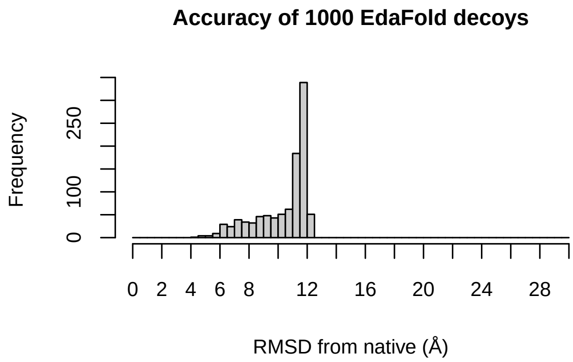

# 1gvp ( all-$\beta$, 87 residues )

Sets of short Rosetta Runs | Long Rosetta Runs | EdaFoldAA

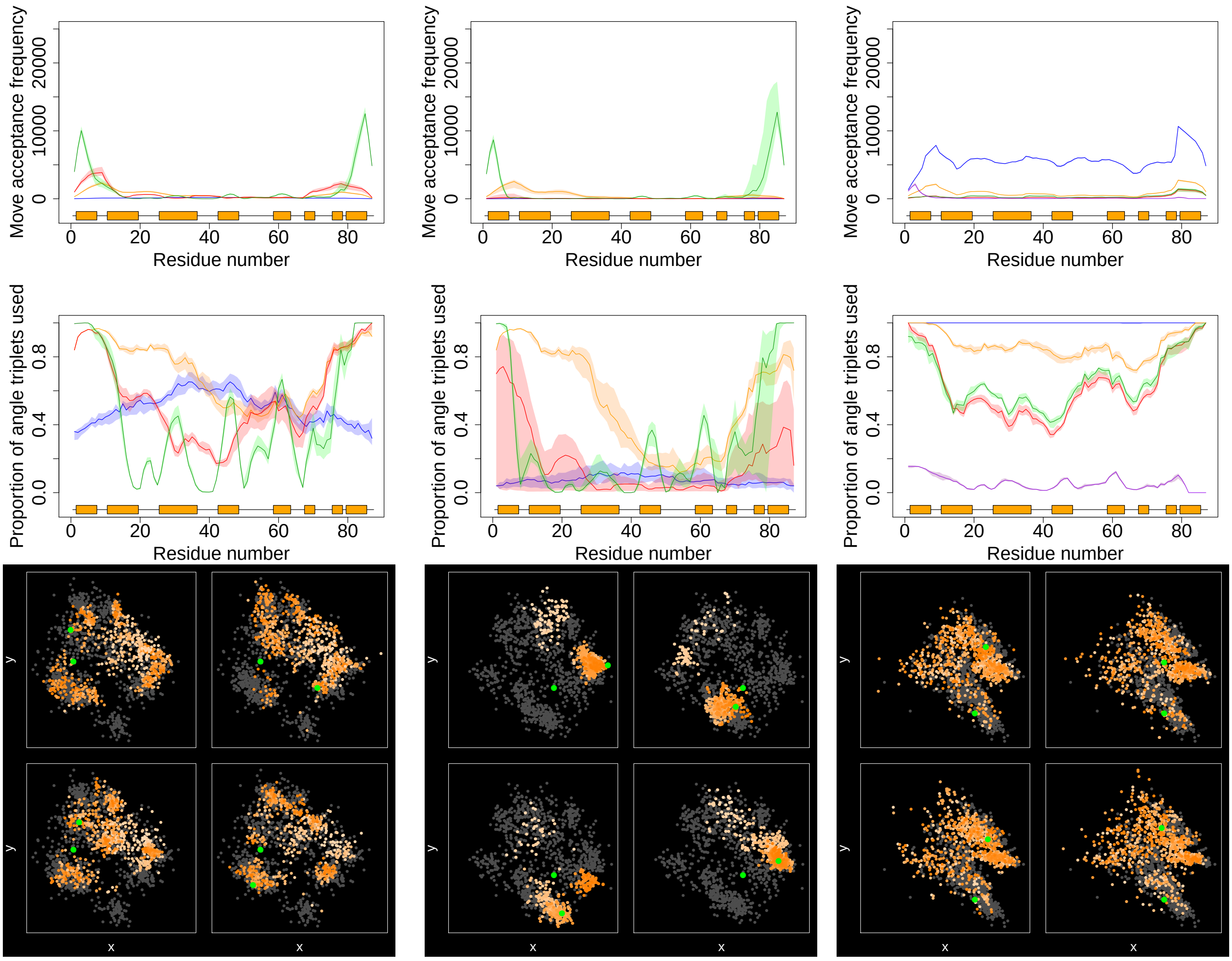


$f = 14.666$ | $f = 24.004$ | $f = 17.393$

**Accuracy of 1000 Rosetta decoys**

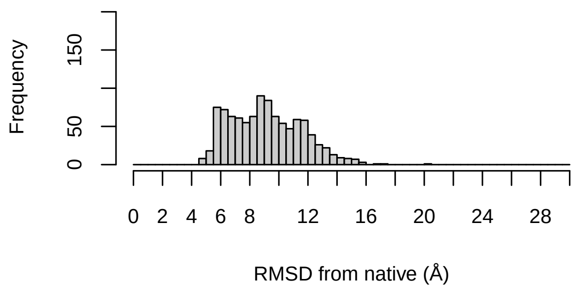


**Accuracy of 1000 EdaFold decoys**

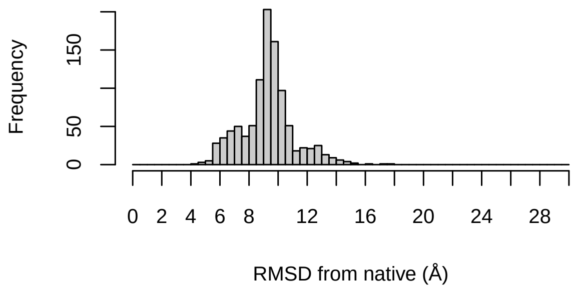

# 1npsA ( all-$\beta$, 88 residues )

Sets of short Rosetta Runs | Long Rosetta Runs | EdaFoldAA

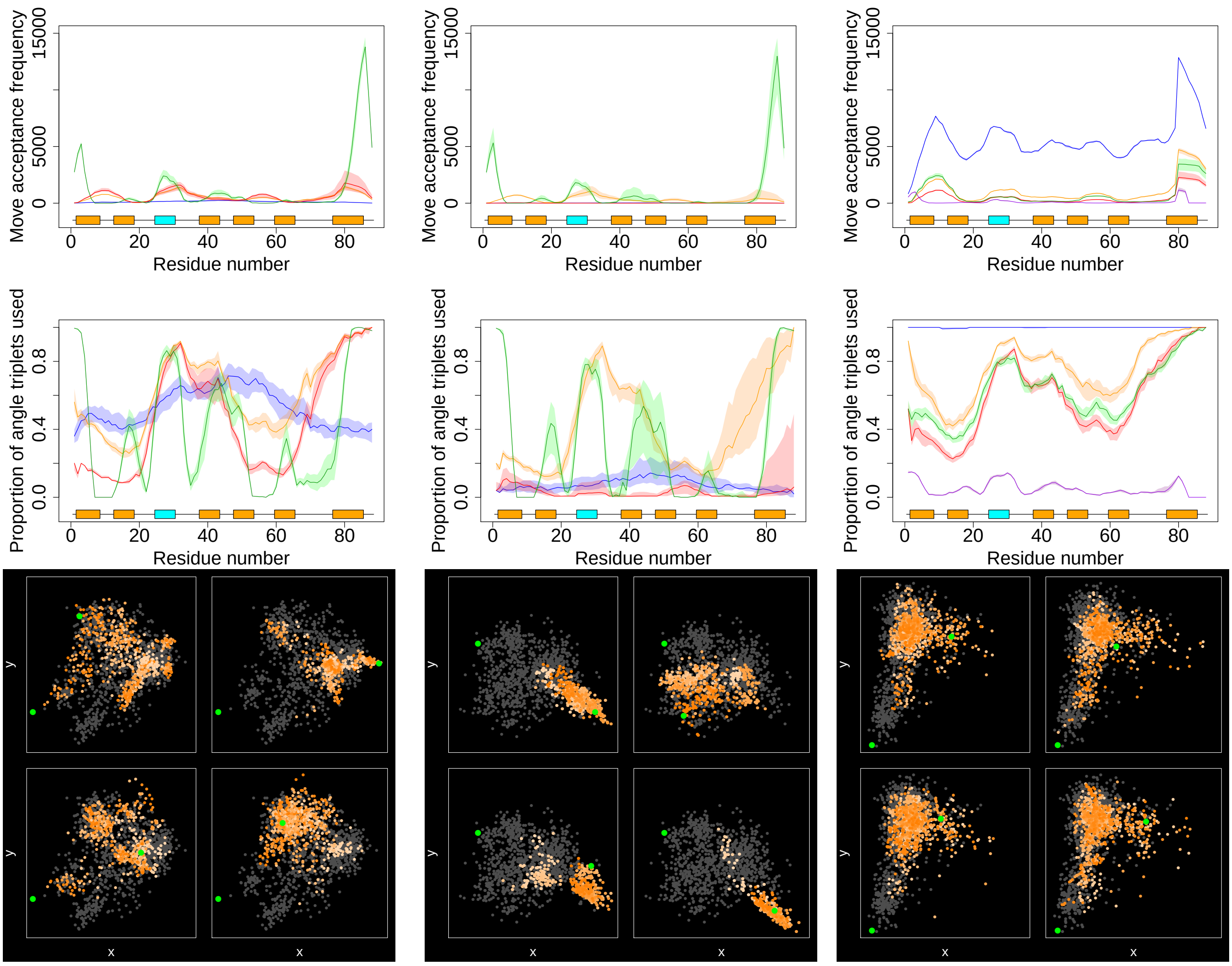


$f$ = 8.9796 | $f$ = 13.345 | $f$ = 9.6742

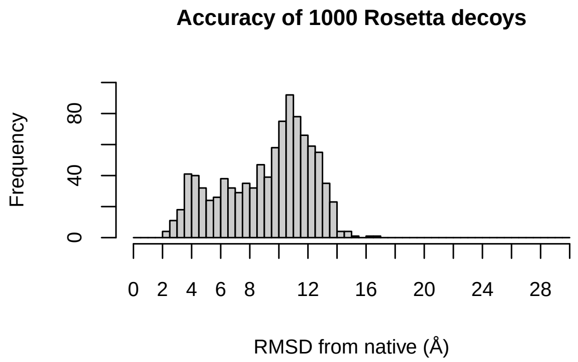


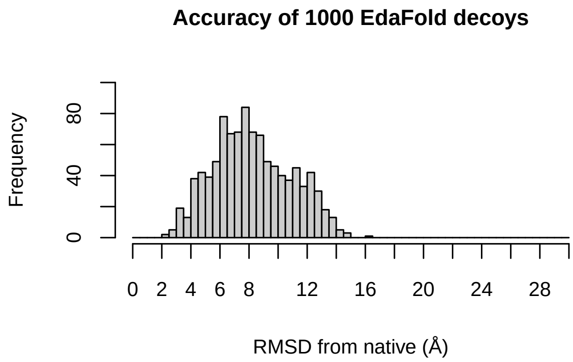

# 1ten ( all-$\beta$, 89 residues )

Sets of short Rosetta Runs | Long Rosetta Runs | EdaFoldAA

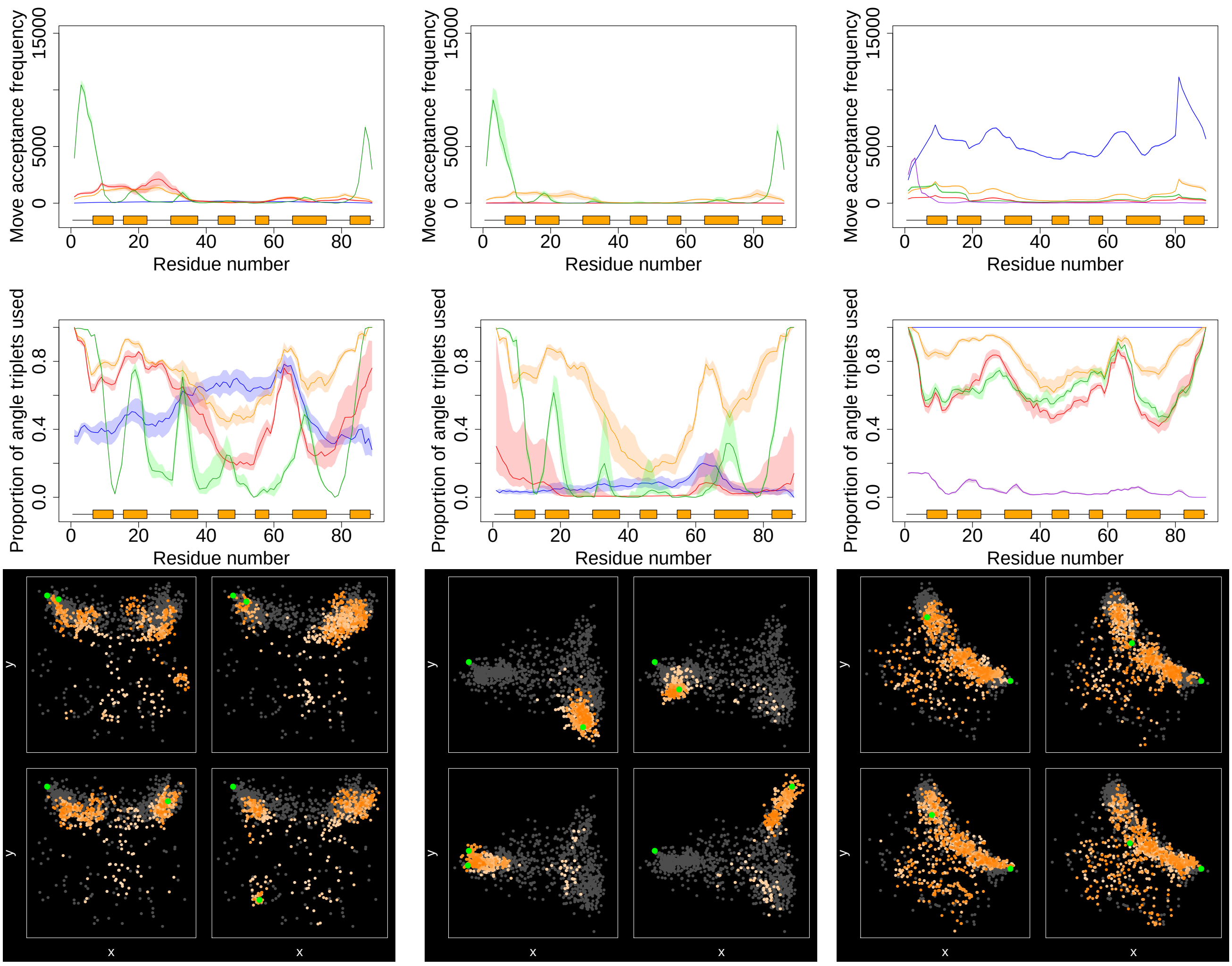


$f = 20.188$ | $f = 28.557$ | $f = 19.697$

**Accuracy of 1000 Rosetta decoys**

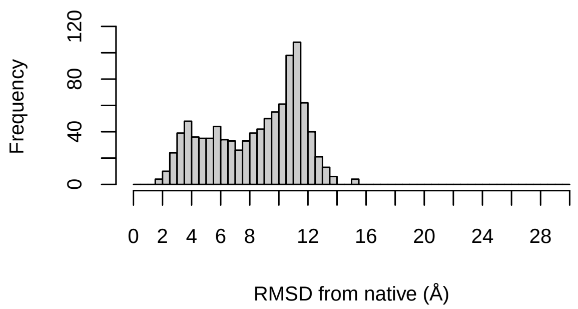


**Accuracy of 1000 EdaFold decoys**

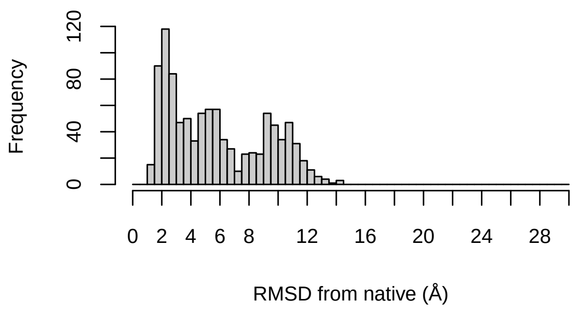

# 1tit ( all-$\beta$, 89 residues )

Sets of short Rosetta Runs | Long Rosetta Runs | EdaFoldAA

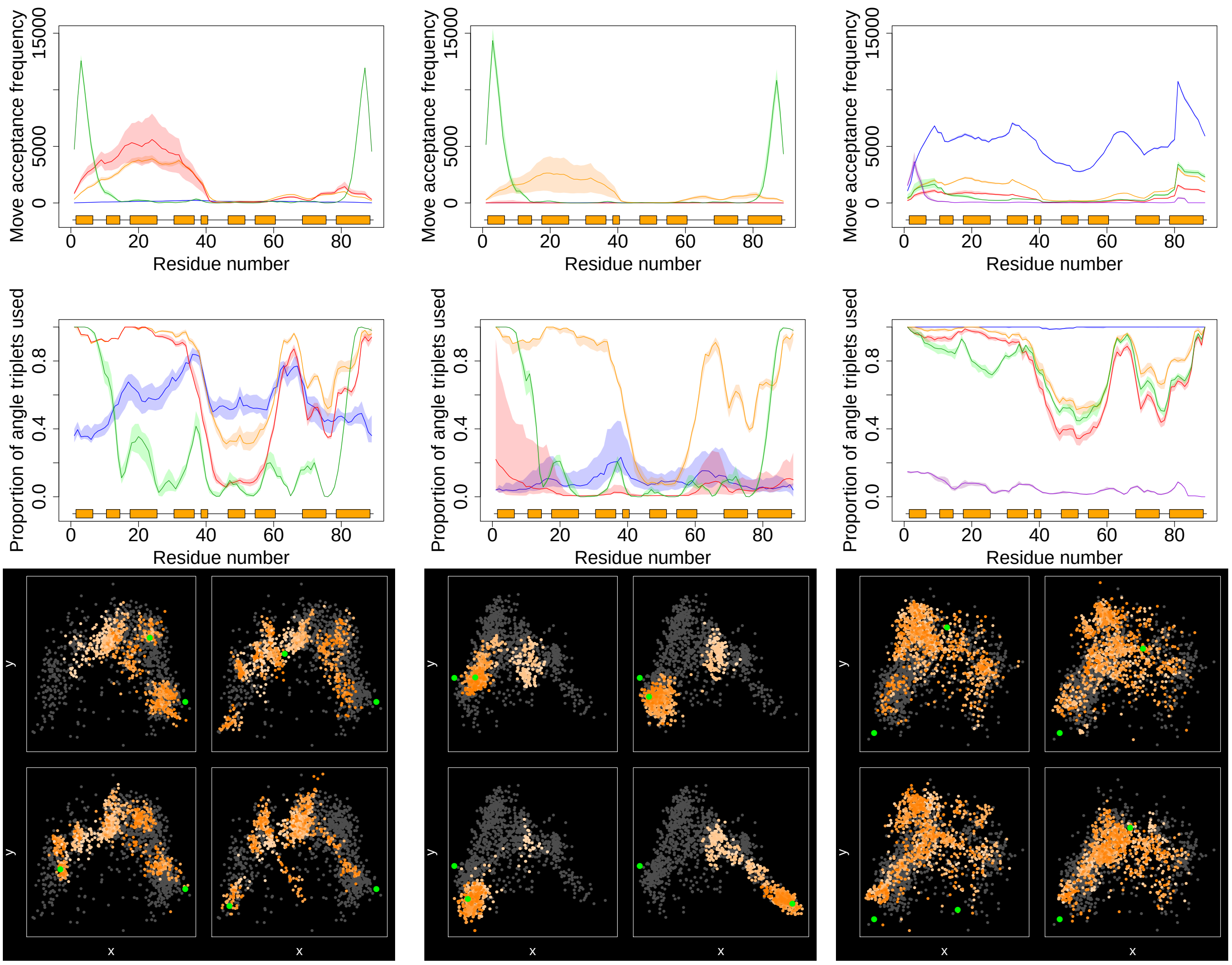


$f = 14.947$ | $f = 23.889$ | $f = 19.248$

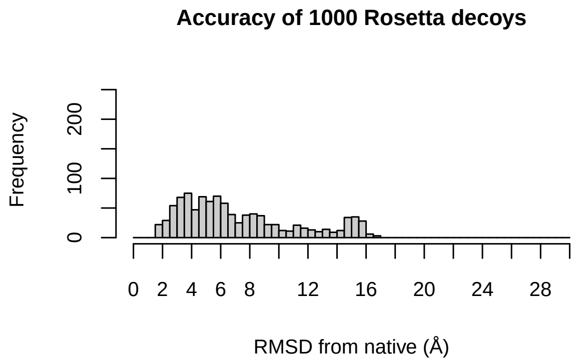


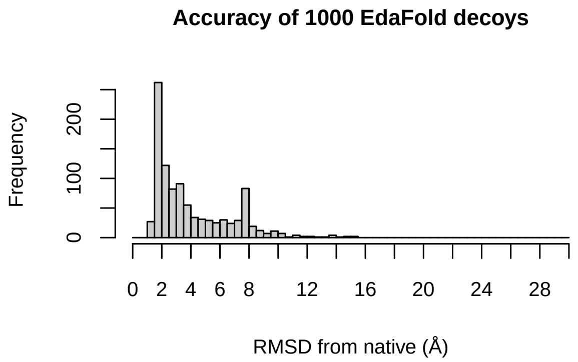

# 1fna ( all-$\beta$, 91 residues )

Sets of short Rosetta Runs | Long Rosetta Runs | EdaFoldAA

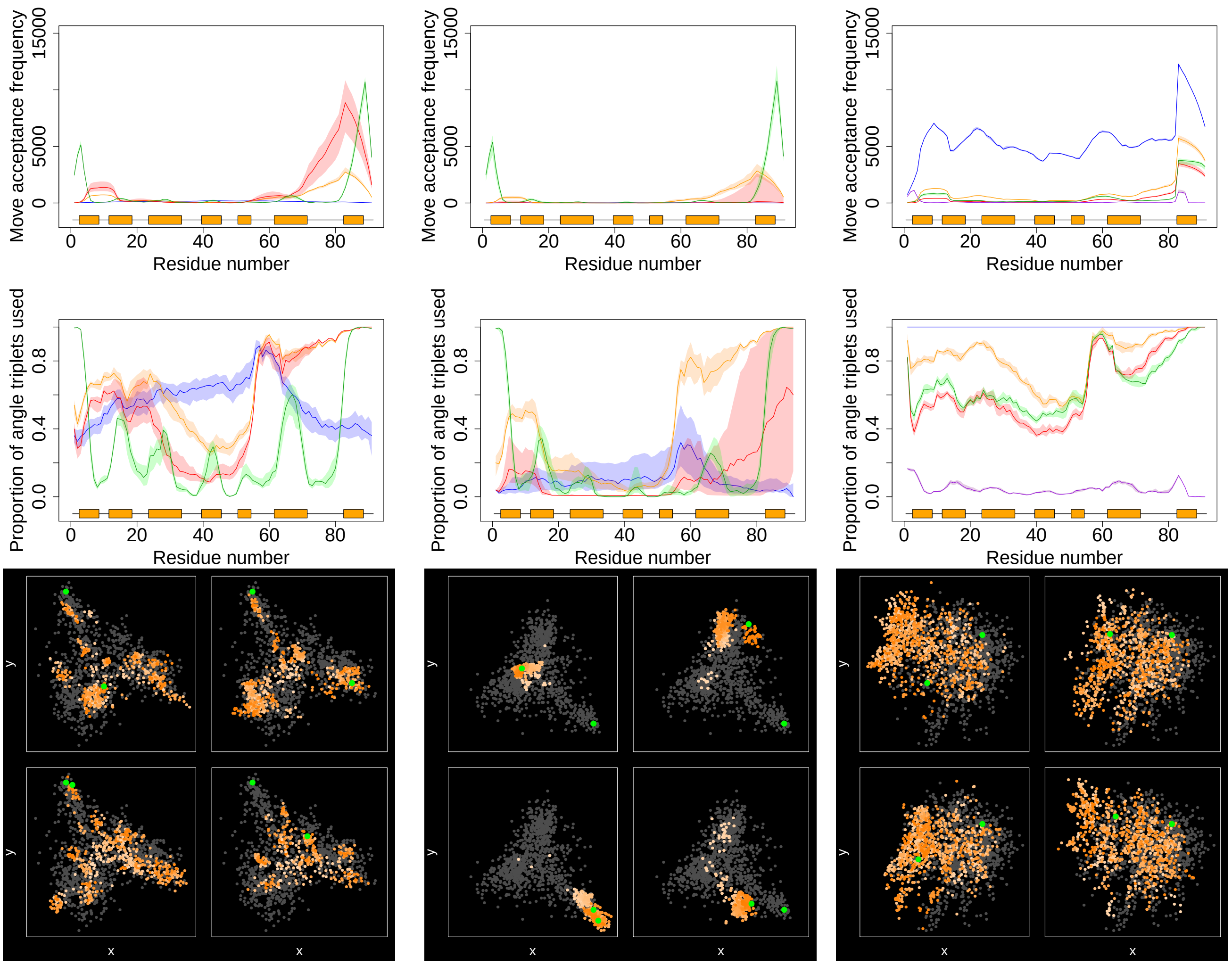


$f$ = 10.478 $\quad$ $f$ = 18.837 $\quad$ $f$ = 9.8416

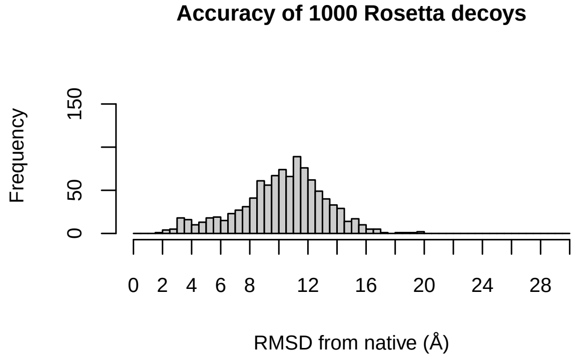


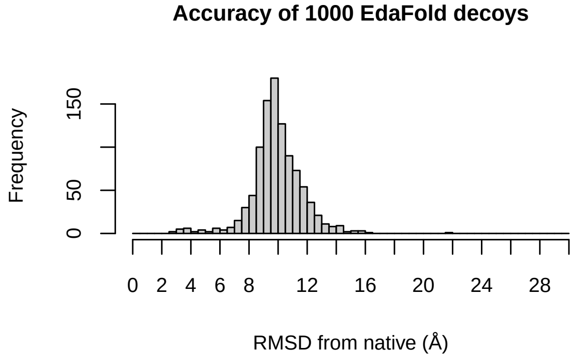

# 1wit ( all-$\beta$, 93 residues )

Sets of short Rosetta Runs | Long Rosetta Runs | EdaFoldAA

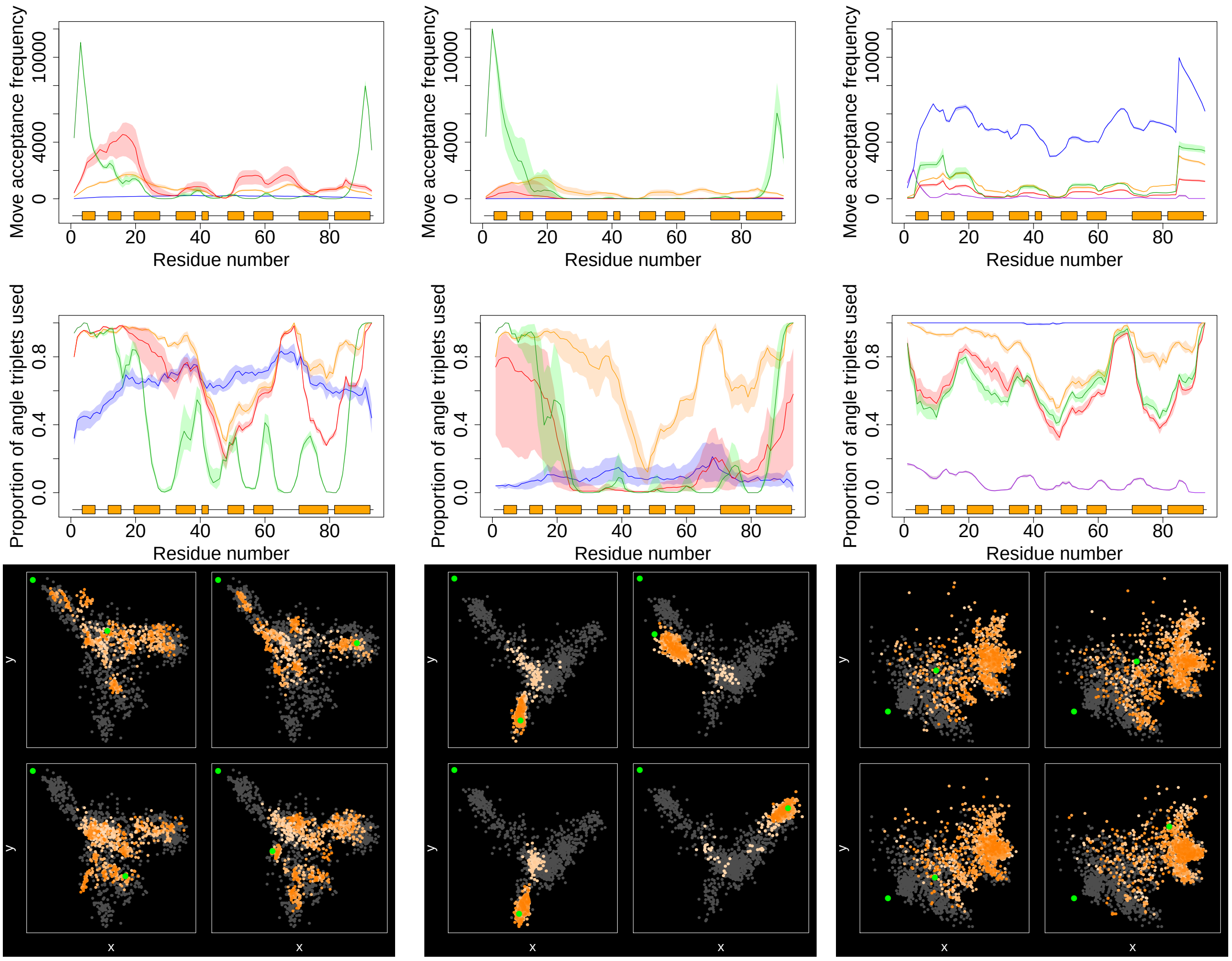


$f$ = 11.832 | $f$ = 23.05 | $f$ = 16.614

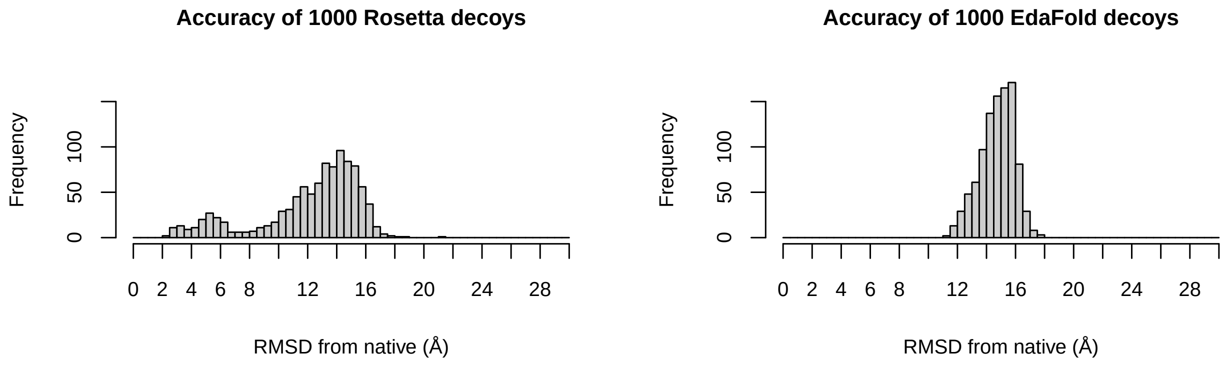

# 1who ( all-$\beta$, 94 residues )

Sets of short Rosetta Runs | Long Rosetta Runs | EdaFoldAA

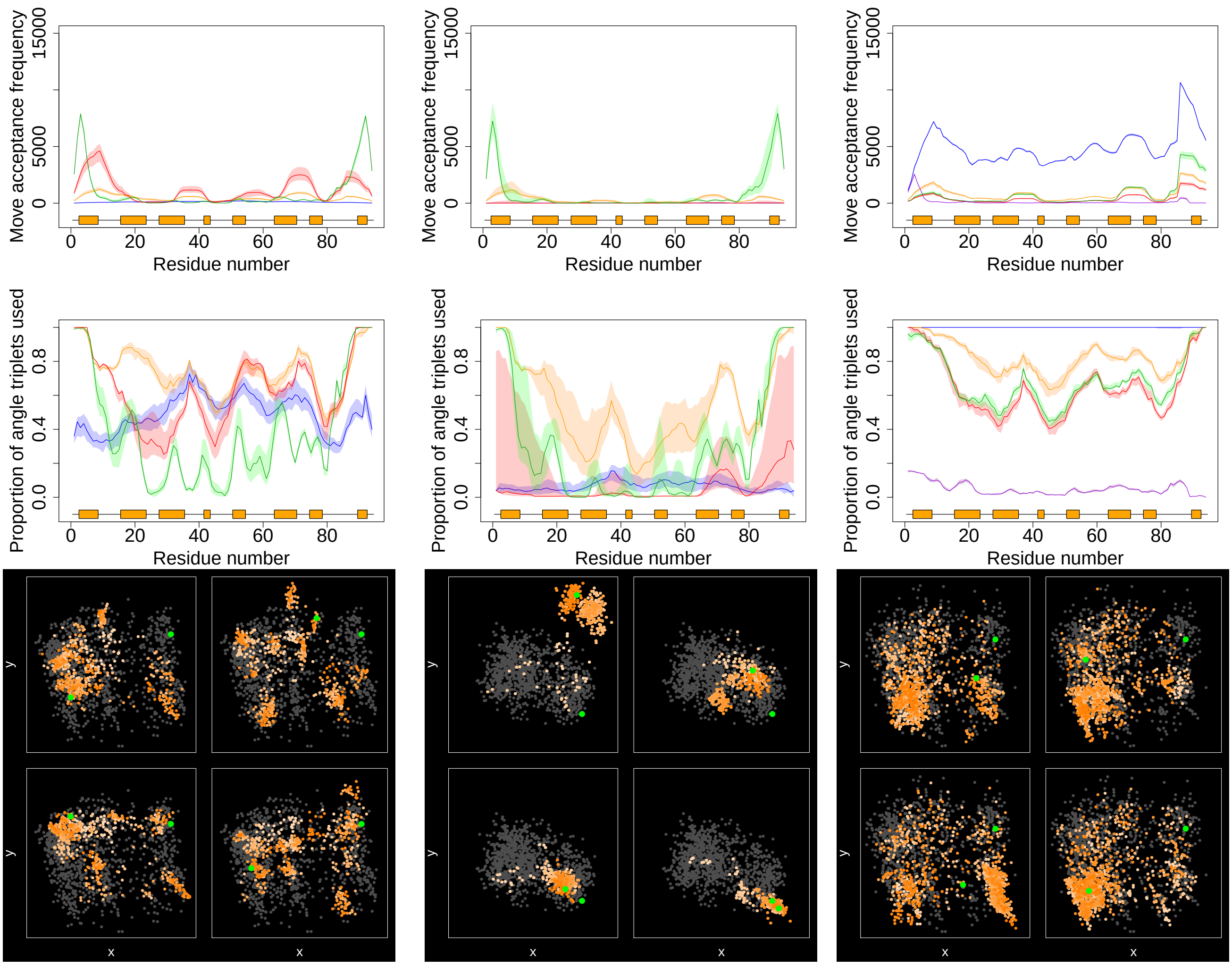


$f$ = 10.249 | $f$ = 15.586 | $f$ = 13.552

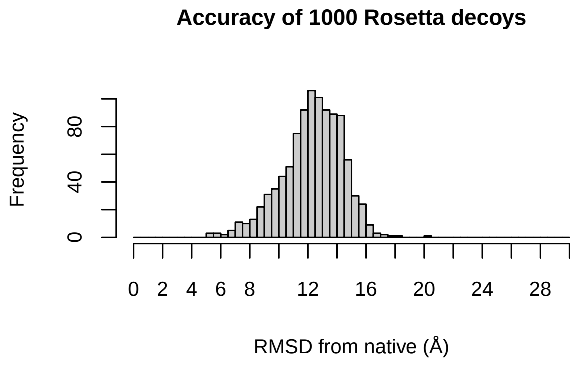


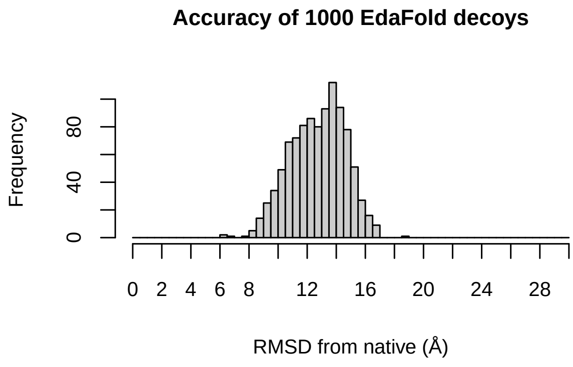

# 1tul ( all-$\beta$, 102 residues )

Sets of short Rosetta Runs | Long Rosetta Runs | EdaFoldAA

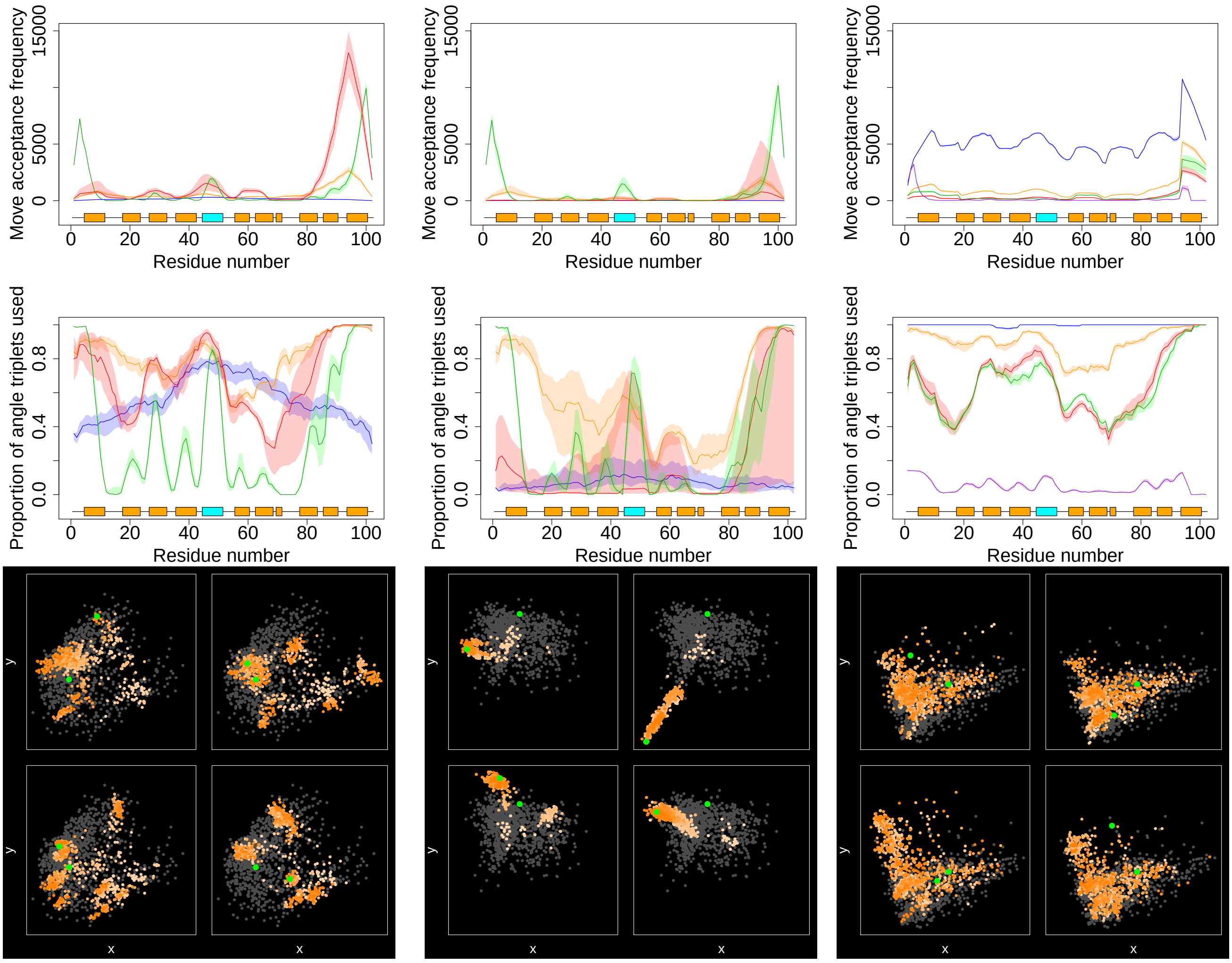


$f$ = 10.025 | $f$ = 15.081 | $f$ = 10.313

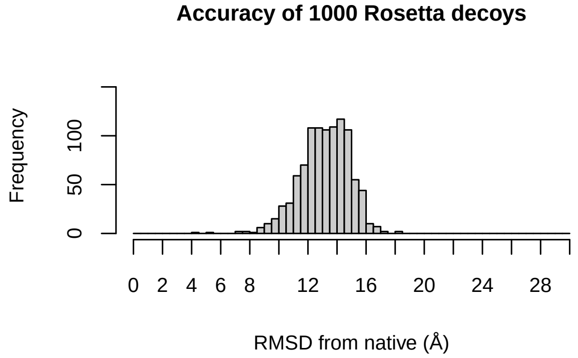


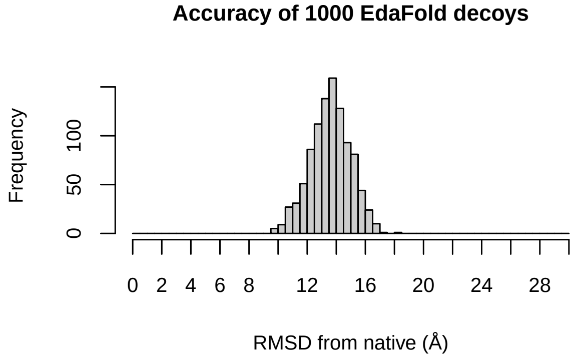

## 1ail ( all-$\alpha$, 70 residues )

Note: MDS analysis could not be carried out on 1ail for sets of short Rosetta runs despite reducing the size of the input dataset to 8 sets of runs; the dataset was too large due to very high move acceptance frequencies.

Sets of short Rosetta Runs | Long Rosetta Runs | EdaFoldAA

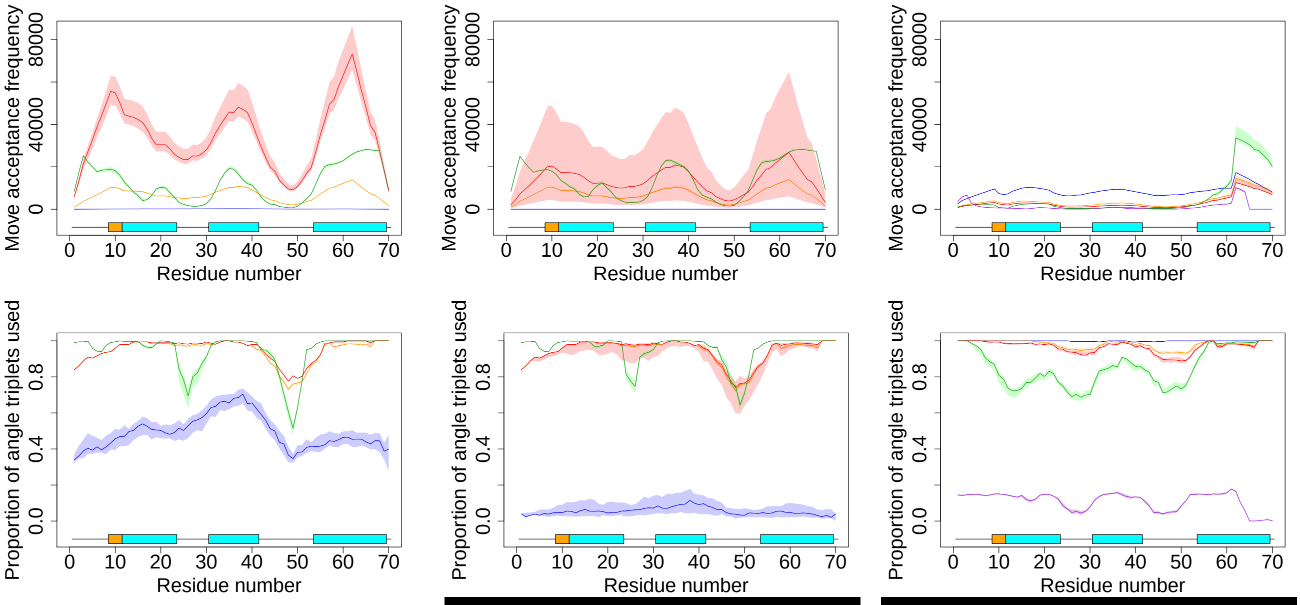


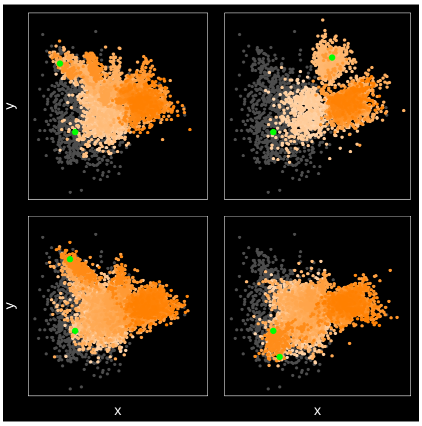

$f = 9.4914$

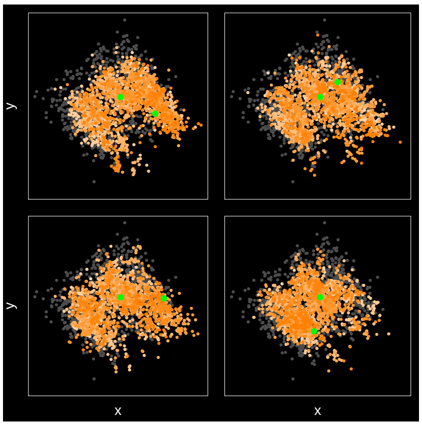

$f = 8.015$

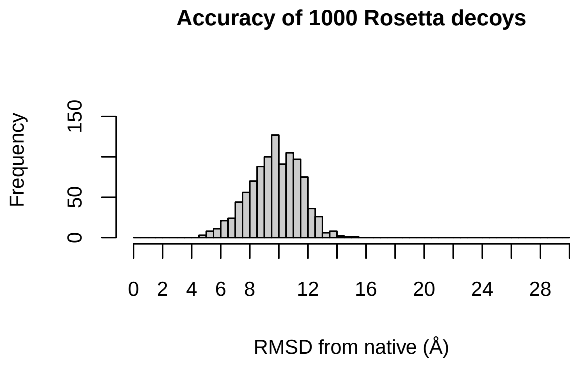


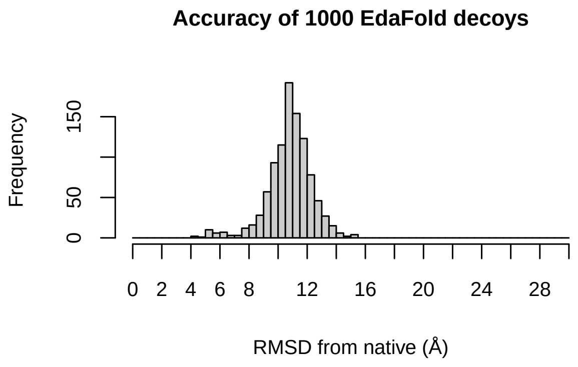

# References

[1] Murzin AG, Brenner SE, Hubbard T, Chothia C. SCOP: A structural classification of proteins database for the investigation of sequences and structures. Journal of Molecular Biology. 1995;247(4):536 – 540. Available from: http://www.sciencedirect.com/science/article/pii/S0022283605801342.

[2] Andreeva A, Howorth D, Chandonia JM, Brenner SE, Hubbard TJP, Chothia C, et al. Data growth and its impact on the SCOP database: new developments. Nucleic Acids Research. 2008;36(suppl 1):D419–D425. Available from: http://nar.oxfordjournals.org/content/36/suppl_1/D419.abstract.

[3] Jones DT. Protein secondary structure prediction based on position-specific scoring matrices. Journal of Molecular Biology. 1999;292(2):195 – 202. Available from: http://www.sciencedirect.com/science/article/pii/S0022283699930917.
