# Supplementary material for: Toward a detailed understanding of search trajectories in fragment assembly approaches to protein structure prediction
Source: Proteins. 2016 Feb 23;84(4):411–26. doi: 10.1002/prot.24987 (PMC4982100; doi:10.1002/prot.24987)
Supplement: Supplementary file 3 — Supporting Information [file PROT-84-411-s003.pdf]

Supplementary Information

1 Weighted entropy measure for an n-state Markov model

The Shannon entropy [1] E for an n-state system is given by

$$E = \sum_{i=1}^{n} -p_i \ln p_i \ ,$$

where each p_i is the probability associated with the system being in state i.

For a Markov state model of n_{clust} states (or clusters), we can define a transition count or frequency matrix $\mathbf{T}$. The element in row i and column j in this matrix (t_{ij}) corresponds to the observed frequency of transitions from state (or cluster) i to state j. This information is obtained by looking at the clustering assignments for each data point in a trajectory.

Next, we estimate the probability that the system is undergoing a transition between any given pair of clusters by constructing a matrix of probabilities, $\mathbf{P}$, with each element given by:

$$p_{ij} = \frac{t_{ij}}{N_{transitions}}, \quad \text{where}$$

$$N_{transitions} = \sum_{i=1}^{n_{clust}} \sum_{j=1}^{n_{clust}} t_{ij}, \quad \text{and}$$

$$\sum_{i=1}^{n_{clust}} \sum_{j=1}^{n_{clust}} p_{ij} = 1.$$

Thus, we can calculate the total entropy E across all possible transitions, by summing the Shannon entropy for each individual transition, as follows:

$$E \ = \ \sum_{i=1}^{n_{clust}} \sum_{j=1}^{n_{clust}} e_{ij} \ = \ \sum_{i=1}^{n_{clust}} \sum_{j=1}^{n_{clust}} -p_{ij} \ln p_{ij}$$

Shannon [35] showed that this quantity is maximised when every p_{ij} is equal (i.e. every possible transition is equally likely). This implies that

$$\text{each} \quad p_{ij} = \frac{1}{(n_{clust})^2} \ .$$

In our case, the clusters correspond to structural states identified during the PAM clustering procedure. Since cluster medoids differ from each other by varying amounts, we wish to weight the entropy values for transitions between a given pair of clusters according to the amount of structural change introduced by that particular transition. Thus, a transition corresponding to a large structural change should be upweighted compared to another transition that does not

correspond to much structural change. With the weights included, the entropy corresponding to a transition between clusters i and j is

$$e_{ij} = -w_{ij} \cdot p_{ij} \ln p_{ij}$$

Guiaşu [56] gives an account of some properties of such a weighted Shannon entropy measure. In particular, the author provides expressions for finding the maximum entropy for a given set of weights, as well as an expression for the value of each probability value that maximises the total entropy. This is important, since the maximum attainable entropy depends on the values of the weights. For this reason, we normalise the weighted entropy values by the theoretical maximum entropy possible for a given set of weights. By Theorem 2 in [56], we have

$$E_{max} = \alpha + \sum_{i=1}^{n_{clust}} \sum_{j=1}^{n_{clust}} w_{ij}\, p_{ij}, \quad \text{where}$$

$$p_{ij} = e^{-(\alpha/w_{ij})-1},$$

and α is a Lagrange multiplier introduced while deriving these expressions. Since the sum of all the transition probabilities p_{ij} is 1, we use this to solve for α:

$$\sum_{i=1}^{n_{clust}} \sum_{j=1}^{n_{clust}} p_{ij} \;=\; \sum_{i=1}^{n_{clust}} \sum_{j=1}^{n_{clust}} e^{-(\alpha/w_{ij})-1} = 1.$$

We use Brent's algorithm [57,58], as implemented in the `uniroot` function in R version 3.1.0, to find the value of α, and thus, the value of the maximum entropy possible for a given set of weights.

We define each weight w_{ij} as the Hamming distance between the corresponding pair of medoids identified during the clustering process. Since we cannot supply zero weights in the equations above, we set self-dissimilarities to a small positive value (10^{-5}).

2 Hamming distance between contact maps

We require a measure of dissimilarity between conformational states that captures differences at the fold level. We explored a few different ways of describing the dissimilarity between conformations, for use in MDS plot construction and clustering for MSM construction. These included the backbone RMSD values between states, and the Hamming distance between binarised contact maps. Below are examples of MDS plots constructed using these measures, for one Rosetta trajectory for the target 1pgx.

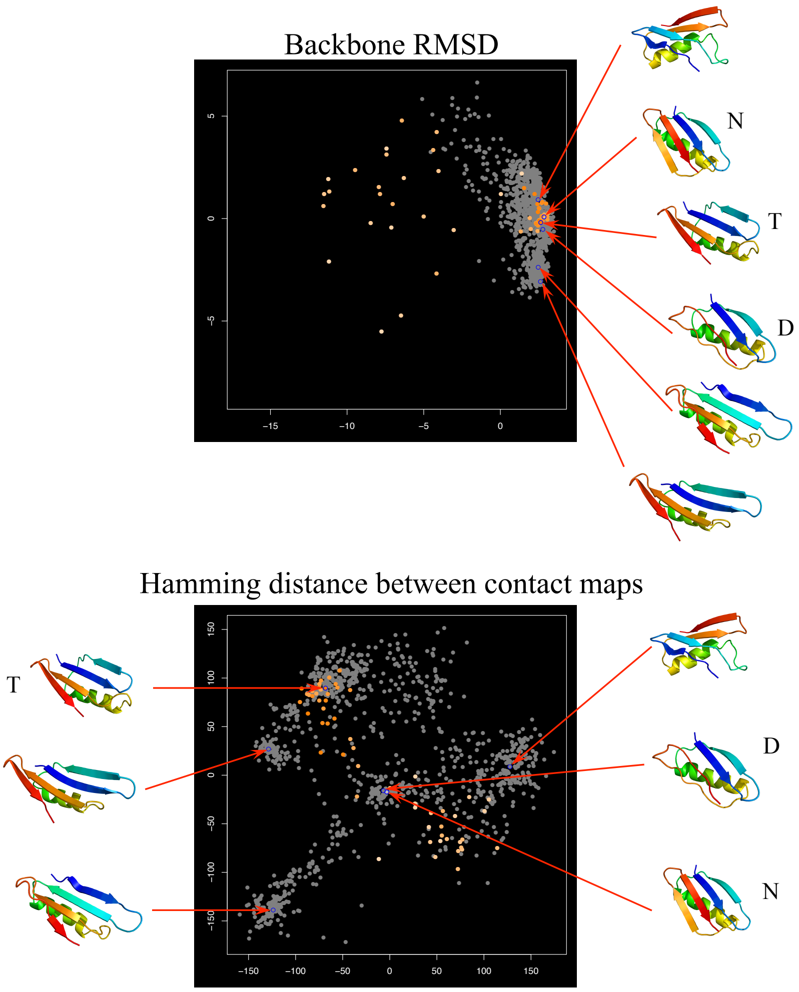

Figure S1: Comparison between dissimilarity measures for MDS plot generation, using a single Rosetta trajectory for 1pgx. The endpoint of the trajectory (T) has an incorrect arrangement of beta strands as compared to the native (N), while another low-resolution decoy (D) has the correct arrangement. The relative locations of these structures on the plots generated using the RMSD measure suggest that they are all highly similar, whereas the plot using the Hamming distance metric correctly captures the fact that the structures N and D are similar, while T is not. Thus, the Hamming distance is a good measure of fold-level dissimilarity for this target. The positions of another three structures with distinct strand arrangements are also shown.

3 Choice of cluster partitioning

The partitioning of the pool of structures generated for different targets influences how much information relating to transitions is captured by the Markov state model. Too coarse a partitioning (too few clusters) risks missing transitions between smaller "sub-clusters". On the other hand, too fine a partitioning (too many clusters) risks incorrectly classifying transitions between otherwise similar states as transitions between distinct clusters, rather than a self-transition within a single cluster.

On average, our entropy measure is seen to decrease with the number of clusters used (Fig. S2), but starts to tail off for higher numbers. To avoid the possibility of incorrect classification at higher cluster numbers, we used a fixed number of 20 clusters for all targets.

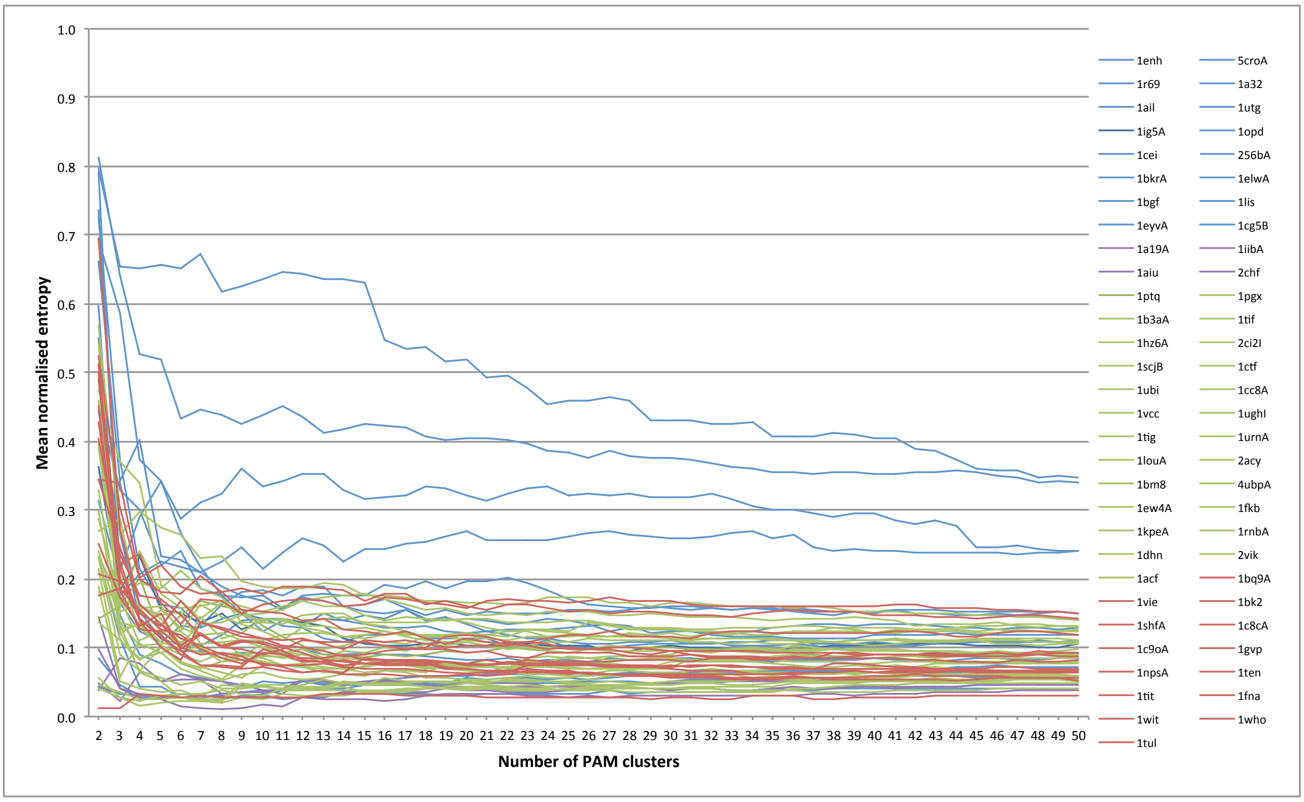

Figure S2: Mean entropy (8 short Rosetta trajectories) versus number of PAM clusters for all targets. All-α targets are in blue, $\alpha + \beta$ targets are in green, α/β targets are in purple and all-β targets are in red.

While it is possible that we may lose some detail by classifying some transitions incorrectly, using a fixed number of clusters for all targets allows us to compare their entropy values. This is important when analysing the properties of the search algorithm for targets of different sizes and structural types. Further, the problem of misclassification is somewhat mitigated by our weighting of entropic contributions according to the corresponding structural change realised (see section 1 of this document).

4 Entropy data for all targets

Table S1: Entropy data for all targets in our dataset. Targets are sorted by secondary structural class, and then by length. Values are given as median and median absolute deviation (MAD) over 8 replicates. Entropy values could not be calculated for sets of short Rosetta runs on the target 1ail, since the dataset was too large for the PAM implementation in R.

SS class	Length	PDB ID	Sets of short Rosetta runs		Single long Rosetta runs		EdaFoldAA (all iterations)		EdaFoldAA (iteration 1)		EdaFoldAA (iteration 2)		EdaFoldAA (iteration 3)		EdaFoldAA (iteration 4)	
			Median	MAD	Median	MAD	Median	MAD	Median	MAD	Median	MAD	Median	MAD	Median	MAD
A	54	1enh	0.443	0.023	0.412	0.013	0.508	0.020	0.446	0.006	0.474	0.015	0.477	0.031	0.456	0.043
A	63	1r69	0.589	0.010	0.516	0.027	0.646	0.003	0.504	0.014	0.616	0.023	0.614	0.015	0.609	0.016
A	65	1a32	0.340	0.019	0.348	0.020	0.470	0.009	0.461	0.023	0.471	0.024	0.416	0.013	0.388	0.029
A	70	1ail	NA	NA	0.188	0.057	0.340	0.006	0.258	0.013	0.296	0.027	0.311	0.029	0.327	0.024
A	70	1utg	0.175	0.036	0.185	0.098	0.301	0.015	0.226	0.018	0.273	0.020	0.290	0.015	0.280	0.036
A	75	1ig5A	0.351	0.010	0.422	0.010	0.426	0.010	0.382	0.011	0.395	0.007	0.386	0.007	0.387	0.015
A	85	1cei	0.165	0.022	0.157	0.043	0.418	0.017	0.303	0.038	0.383	0.019	0.370	0.041	0.383	0.032
A	106	256bA	0.399	0.010	0.443	0.036	0.436	0.028	0.285	0.018	0.402	0.019	0.437	0.020	0.405	0.024
A	108	1bkrA	0.113	0.031	0.057	0.008	0.367	0.003	0.251	0.017	0.298	0.030	0.361	0.041	0.365	0.036
A	117	1elwA	0.479	0.015	0.408	0.007	0.492	0.010	0.559	0.026	0.490	0.013	0.424	0.010	0.367	0.008
A	118	1bgf	0.135	0.010	0.142	0.027	0.380	0.010	0.288	0.026	0.335	0.042	0.355	0.031	0.345	0.031
A	125	1lis	0.192	0.024	0.202	0.033	0.302	0.008	0.263	0.012	0.272	0.006	0.266	0.016	0.267	0.023
A	131	1eyvA	0.135	0.019	0.119	0.044	0.283	0.027	0.217	0.007	0.243	0.012	0.274	0.042	0.258	0.018
A	141	1cg5B	0.104	0.008	0.094	0.027	0.392	0.026	0.335	0.043	0.282	0.026	0.366	0.019	0.383	0.047
A/B	89	1a19A	0.100	0.026	0.056	0.010	0.434	0.020	0.319	0.021	0.405	0.043	0.407	0.013	0.382	0.028
A/B	103	1iibA	0.072	0.016	0.040	0.007	0.226	0.009	0.142	0.017	0.199	0.015	0.233	0.021	0.214	0.017
A/B	105	1aiu	0.155	0.010	0.096	0.014	0.388	0.015	0.313	0.037	0.351	0.022	0.350	0.020	0.339	0.015
A/B	128	2chf	0.091	0.004	0.055	0.024	0.359	0.012	0.248	0.015	0.312	0.017	0.329	0.014	0.336	0.019
A+B	50	1ptq	0.343	0.013	0.221	0.014	0.396	0.006	0.342	0.032	0.345	0.019	0.350	0.012	0.354	0.016
A+B	55	1pgx	0.232	0.016	0.204	0.027	0.414	0.008	0.326	0.010	0.391	0.021	0.384	0.009	0.347	0.019
A+B	55	1b3aA	0.195	0.011	0.243	0.033	0.338	0.009	0.274	0.030	0.313	0.022	0.316	0.021	0.305	0.036
A+B	55	5croA	0.353	0.008	0.351	0.008	0.446	0.015	0.426	0.015	0.426	0.022	0.413	0.027	0.377	0.029
A+B	59	1tif	0.274	0.023	0.213	0.010	0.289	0.034	0.189	0.021	0.341	0.039	0.243	0.065	0.218	0.048
A+B	61	1hz6A	0.145	0.023	0.100	0.016	0.384	0.031	0.375	0.027	0.388	0.012	0.306	0.035	0.283	0.031
A+B	62	2ci2I	0.207	0.017	0.167	0.024	0.419	0.005	0.346	0.026	0.388	0.020	0.370	0.020	0.350	0.019
A+B	66	1scjB	0.190	0.009	0.185	0.012	0.339	0.011	0.273	0.025	0.344	0.002	0.300	0.026	0.272	0.010
A+B	68	1ctf	0.134	0.014	0.084	0.016	0.349	0.021	0.299	0.023	0.350	0.028	0.317	0.032	0.278	0.020
A+B	71	1ubi	0.223	0.010	0.234	0.036	0.477	0.017	0.455	0.015	0.445	0.021	0.407	0.048	0.374	0.038
A+B	72	1cc8A	0.181	0.017	0.124	0.019	0.243	0.007	0.234	0.015	0.263	0.007	0.218	0.015	0.195	0.005
A+B	77	1vcc	0.160	0.008	0.146	0.025	0.398	0.015	0.250	0.029	0.363	0.039	0.369	0.013	0.350	0.033
A+B	82	1ughI	0.136	0.013	0.096	0.045	0.387	0.009	0.364	0.029	0.369	0.022	0.323	0.015	0.286	0.031
A+B	85	1opd	0.101	0.007	0.088	0.024	0.355	0.015	0.298	0.028	0.310	0.026	0.317	0.033	0.337	0.019
A+B	88	1tig	0.087	0.025	0.042	0.007	0.345	0.009	0.221	0.043	0.295	0.040	0.325	0.021	0.322	0.025
A+B	90	1urnA	0.105	0.008	0.164	0.050	0.390	0.031	0.330	0.045	0.358	0.024	0.364	0.019	0.318	0.029
A+B	92	1louA	0.092	0.010	0.049	0.017	0.364	0.017	0.300	0.020	0.322	0.060	0.332	0.015	0.301	0.015
A+B	98	2acy	0.157	0.015	0.088	0.024	0.402	0.015	0.328	0.012	0.360	0.017	0.373	0.006	0.348	0.011
A+B	99	1bm8	0.137	0.013	0.071	0.004	0.330	0.023	0.317	0.026	0.305	0.028	0.270	0.023	0.254	0.033
A+B	100	4ubpA	0.161	0.022	0.128	0.029	0.355	0.021	0.308	0.029	0.329	0.029	0.325	0.049	0.290	0.022
A+B	106	1ew4A	0.130	0.024	0.084	0.016	0.344	0.028	0.342	0.014	0.324	0.029	0.290	0.028	0.265	0.027
A+B	107	1fkb	0.084	0.014	0.026	0.010	0.430	0.023	0.346	0.025	0.387	0.028	0.416	0.041	0.368	0.018
A+B	108	1kpeA	0.072	0.011	0.027	0.011	0.311	0.012	0.250	0.017	0.257	0.025	0.296	0.035	0.276	0.020
A+B	109	1rnbA	0.263	0.009	0.099	0.012	0.336	0.014	0.279	0.023	0.322	0.005	0.291	0.030	0.251	0.017
A+B	121	1dhn	0.063	0.007	0.028	0.012	0.424	0.007	0.386	0.009	0.390	0.031	0.349	0.033	0.348	0.031
A+B	122	2vik	0.114	0.019	0.038	0.008	0.410	0.020	0.295	0.045	0.371	0.032	0.382	0.044	0.353	0.021
A+B	125	1acf	0.074	0.012	0.047	0.007	0.280	0.019	0.277	0.022	0.269	0.043	0.243	0.017	0.207	0.053
B	51	1bq9A	0.241	0.026	0.155	0.028	0.311	0.008	0.288	0.008	0.319	0.011	0.278	0.010	0.235	0.010
B	56	1vie	0.154	0.014	0.081	0.018	0.304	0.011	0.322	0.012	0.288	0.013	0.250	0.022	0.234	0.026
B	57	1bk2	0.132	0.015	0.142	0.033	0.207	0.014	0.201	0.037	0.302	0.026	0.174	0.007	0.134	0.008
B	59	1shfA	0.135	0.023	0.102	0.044	0.291	0.013	0.200	0.023	0.262	0.017	0.268	0.009	0.251	0.018
B	62	1c8cA	0.174	0.025	0.108	0.018	0.372	0.020	0.321	0.017	0.313	0.015	0.334	0.038	0.316	0.038
B	66	1c9oA	0.118	0.011	0.132	0.013	0.296	0.026	0.341	0.020	0.322	0.022	0.244	0.033	0.192	0.008
B	87	1gvp	0.149	0.021	0.059	0.024	0.324	0.007	0.315	0.020	0.307	0.023	0.269	0.007	0.254	0.014
B	88	1npsA	0.122	0.014	0.040	0.025	0.255	0.012	0.244	0.026	0.224	0.014	0.216	0.018	0.226	0.031
B	89	1ten	0.114	0.024	0.049	0.014	0.313	0.012	0.315	0.044	0.321	0.023	0.280	0.028	0.223	0.017
B	89	1tit	0.070	0.001	0.047	0.009	0.302	0.017	0.252	0.029	0.285	0.033	0.245	0.053	0.244	0.033
B	91	1fna	0.059	0.008	0.024	0.016	0.308	0.019	0.248	0.010	0.267	0.055	0.275	0.030	0.235	0.012
B	93	1wit	0.066	0.006	0.041	0.004	0.213	0.018	0.251	0.023	0.226	0.016	0.170	0.021	0.152	0.019
B	94	1who	0.066	0.015	0.029	0.010	0.283	0.003	0.282	0.024	0.266	0.032	0.234	0.014	0.221	0.012
B	102	1tul	0.044	0.011	0.020	0.013	0.273	0.017	0.235	0.025	0.240	0.026	0.239	0.034	0.234	0.014

5 Statistical analysis of entropy data

We performed statistical analysis on two entropy datasets: one comparing all three running protocols over complete runs, and one comparing all iterations of EdaFold runs. For each protein, there were 8 replicate runs. We analysed the data using the Mack-Skillings test [40], as implemented in the `pMacSkil` function in the `NSM3` package [59] in R version 3.2.0 [30]. This test is able to test for general alternatives in a replicated complete block design, and reduces to the Friedman test [60,61] when the number of replicates in each treatment-block combination is 1. For the post-hoc test, an R program was written to perform the analysis in section 7.10 in [41]. We used the "conservative" version of the multiple-comparisons procedure (Comment 82 in [41]), which guarantees an upper bound on the experimentwise error rate $\alpha = 0.05$.

The post-hoc procedure calculates a critical value of the difference in a measure S between treatments, taking into account the number of blocks, treatments and replicates (and thus the number of comparisons needed). If the magnitude of this difference is greater than the critical value, we reject the null hypothesis that the two treatment effects being compared are equal. The values of S are calculated for each treatment while deriving the Mack-Skillings test statistic, and reflect the consistency with which a given treatment accumulates higher-ranked observations. Since the ranks are calculated within blocks, the values of S will be similar if the ranks falling in all treatments show little or no difference.

Below, we show the output of our statistical procedures for the two datasets. First, we show the results comparing sets of short Rosetta runs, single long Rosetta runs, and complete EdaFold runs:

```
Comparing Rosetta_short, Rosetta_long and EdaFold
Number of blocks: n= 58
Number of treatments: k= 3
Mack-Skillings MS  Statistic:  952.6938
Monte Carlo  (Using  50000 Iterations) upper-tail probability:  0

Mack-Skillings Post-hoc test
Hollander and Wolfe (1999), 7.10
------------------------------------
degrees of freedom: 2

Critical value of absolute difference between
treatment sums of cellwise averages of within-blocks ranks: 65.8354843530447

Observed differences:
                             difference is.significant
Rosetta_short - Rosetta_long      274.0           TRUE
Rosetta_short - EdaFold          -542.5           TRUE
Rosetta_long - EdaFold           -816.5           TRUE
```

It can be seen that all three comparisons indicate significant differences, with EdaFold showing a significantly higher-ranked entropy on average followed by short Rosetta runs and long Rosetta runs (in that order). Note that only 58 out of the 59 proteins were compared, since entropy values could not be calculated for the target 1ail with short Rosetta runs.

Next, we show the results comparing the 4 iterations within EdaFold runs:

```
Comparing Iteration_1, Iteration_2, Iteration_3 and Iteration_4 in EdaFold
Number of blocks: n= 59
Number of treatments: k= 4
Mack-Skillings MS  Statistic:  176.4797
Monte Carlo  (Using  50000 Iterations) upper-tail probability:  0

Mack-Skillings Post-hoc test
Hollander and Wolfe (1999), section 7.10
------------------------------------
degrees of freedom: 3

Critical value of absolute difference between
treatment sums of cellwise averages of within-blocks ranks: 100.581434569209

Observed differences:
                          difference is.significant
Iteration_1 - Iteration_2   -367.000           TRUE
Iteration_1 - Iteration_3   -212.625           TRUE
Iteration_1 - Iteration_4     56.125          FALSE
Iteration_2 - Iteration_3    154.375           TRUE
Iteration_2 - Iteration_4    423.125           TRUE
Iteration_3 - Iteration_4    268.750           TRUE
```

We see that iteration 2 shows a significantly higher-ranked value of entropy than iteration 1, and subsequent iterations show significant decreases as compared to prior iterations. Iteration 4 is not significantly different from iteration 1, suggesting that entropy values typically rise and fall between iterations 1 and 4.

References

[1] Shannon CE. A Mathematical Theory of Communication. Bell System Technical Journal. 1948 July;27(3):379–423.
